# Development of consensus-driven SPIRIT and CONSORT extensions for early phase dose-finding trials: the DEFINE study

**DOI:** 10.1186/s12916-023-02937-0

**Published:** 2023-07-05

**Authors:** Olga Solovyeva, Munyaradzi Dimairo, Christopher J. Weir, Siew Wan Hee, Aude Espinasse, Moreno Ursino, Dhrusti Patel, Andrew Kightley, Sarah Hughes, Thomas Jaki, Adrian Mander, Thomas R. Jeffry Evans, Shing Lee, Sally Hopewell, Khadija Rerhou Rantell, An-Wen Chan, Alun Bedding, Richard Stephens, Dawn Richards, Lesley Roberts, John Kirkpatrick, Johann de Bono, Christina Yap

**Affiliations:** 1 The Institute of Cancer Research, London, UK; 2 Clinical Trials Research Unit, School of Health and Related Research, University of Sheffield, Sheffield, UK; 3 Edinburgh Clinical Trials Unit, Usher Institute, University of Edinburgh, Edinburgh, UK; 4 University Hospitals Coventry & Warwickshire NHS Trust, Coventry, UK; 5 University of Warwick, Coventry, UK; 6 Inserm, Centre de Recherche Des Cordeliers, Sorbonne UniversitéUniversité Paris Cité, 75006 Paris, France; 7 HeKA, Inria Paris, 75015 Paris, France; 8 Unit of Clinical Epidemiology, AP-HP, CHU Robert Debré, CIC-EC 1426 Paris, France; 9 RECaP/F-CRIN, Inserm, 5400 Nancy, France; 10 Patient and Public Involvement and Engagement (PPIE) Lead, Lichfield, UK; 11 University of Birmingham, Birmingham, UK; 12 MRC Biostatistics Unit, University of Cambridge, Cambridge, UK; 13 University of Regensburg, Regensburg, Germany; 14 GSK, Brentford, UK; 15 University of Glasgow, Glasgow, UK; 16 Columbia University, Mailman School of Public Health, New York, USA; 17 Oxford Clinical Trials Research Unit, University of Oxford, Oxford, UK; 18 Medicines and Healthcare Products Regulatory Agency, London, UK; 19 Department of Medicine, Women’s College Research Institute, University of Toronto, Toronto, Canada; 20 Roche Products Ltd, Welwyn Garden City, UK; 21 NCRI, London, UK; 22 Clinical Trials Ontario, Toronto, Canada; 23 The Royal Marsden NHS Foundation Trust, London, UK

**Keywords:** early phase, clinical trials, SPIRIT guideline, CONSORT guideline, dose finding

## Abstract

**Background:**

Early phase dose-finding (EPDF) trials are crucial for the development of a new intervention and influence whether it should be investigated in further trials. Guidance exists for clinical trial protocols and completed trial reports in the SPIRIT and CONSORT guidelines, respectively. However, both guidelines and their extensions do not adequately address the characteristics of EPDF trials. Building on the SPIRIT and CONSORT checklists, the DEFINE study aims to develop international consensus-driven guidelines for EPDF trial protocols (SPIRIT-DEFINE) and reports (CONSORT-DEFINE).

**Methods:**

The initial generation of candidate items was informed by reviewing published EPDF trial reports. The early draft items were refined further through a review of the published and grey literature, analysis of real-world examples, citation and reference searches, and expert recommendations, followed by a two-round modified Delphi process. Patient and public involvement and engagement (PPIE) was pursued concurrently with the quantitative and thematic analysis of Delphi participants’ feedback.

**Results:**

The Delphi survey included 79 new or modified SPIRIT-DEFINE (*n* = 36) and CONSORT-DEFINE (*n* = 43) extension candidate items. In Round One, 206 interdisciplinary stakeholders from 24 countries voted and 151 stakeholders voted in Round Two. Following Round One feedback, one item for CONSORT-DEFINE was added in Round Two. Of the 80 items, 60 met the threshold for inclusion (≥ 70% of respondents voted critical: 26 SPIRIT-DEFINE, 34 CONSORT-DEFINE), with the remaining 20 items to be further discussed at the consensus meeting. The parallel PPIE work resulted in the development of an EPDF lay summary toolkit consisting of a template with guidance notes and an exemplar.

**Conclusions:**

By detailing the development journey of the DEFINE study and the decisions undertaken, we envision that this will enhance understanding and help researchers in the development of future guidelines. The SPIRIT-DEFINE and CONSORT-DEFINE guidelines will allow investigators to effectively address essential items that should be present in EPDF trial protocols and reports, thereby promoting transparency, comprehensiveness, and reproducibility.

**Trial registration:**

SPIRIT-DEFINE and CONSORT-DEFINE are registered with the EQUATOR Network (https://www.equator-network.org/).

**Supplementary Information:**

The online version contains supplementary material available at 10.1186/s12916-023-02937-0.

## Background

Early phase dose-finding (EPDF) trials, also typically referred to as phase I, I/II, or dose-escalation trials, are critical in clinical therapy development for a range of interventions that can be given in different doses and be pharmacological (chemical or biological, e.g. drugs, vaccines, cell therapies, gene therapies), non-pharmacological (e.g. radiotherapy, rehabilitation, digital therapies), or a combination thereof. Conducted in healthy volunteers or in participants with a condition or disease, these trials involve interim dosing adaptations (e.g. escalate/de-escalate) and generate data on safety and other information, such as pharmacokinetics, pharmacodynamics, and clinical activity, to enable developers to choose a suitable dosage(s) (dose and schedule) for further clinical testing.

Study protocols can vary greatly in content and quality despite their importance. Incomplete or unclear information in study protocols and final reports hinders the interpretability and replicability of EPDF trials and may impact the overall clinical development timeline as well as lead to erroneous conclusions on safety and efficacy and compromise the safety of trial participants [1, 2].

Trial designs of EPDF trials are evolving. There has been a rise in the use of seamless designs (integrated protocols with several components or phases within a trial) [3], as well as advanced model-assisted or model-based designs (1.6% of published phase I oncology trials in 1991–2006 [4] compared to 8.6% by 2014–2019 [5]). Such trials come with increased complexity and require further transparency in their protocols and trial reports to help readers understand the design, reproduce methods and interpret findings [1, 6, 7].

The Standard Protocol Items: Recommendations for Interventional Trials (SPIRIT) 2013 [8] statement and the CONsolidated Standards Of Reporting Randomised Trials (CONSORT) 2010 [4] statement have been instrumental in promoting complete and transparent reporting of the minimum essential content in trial protocols and trial reports. Neither the original guidance nor their extensions adequately cover the features of EPDF trials [1, 9]. The DosE-FIndiNg Extensions (DEFINE) study aims to build on the existing SPIRIT 2013 and CONSORT 2010 statements to meet this need and enhance the quality of EPDF trial protocols and their reporting of results across all disease areas [10].

This paper describes the processes, methods and results for the development stages of the DEFINE study. The subsequent consensus meeting and the final checklists will be covered in the forthcoming DEFINE-SPIRIT and DEFINE-CONSORT statement papers.

### Overall project aim

The overall aim of this research is to develop evidence-based and consensus-driven guidelines for trial protocols (SPIRIT-DEFINE) and trial reports (CONSORT-DEFINE) for EPDF trials across all disease areas and disseminate them to stakeholders [1].

## Methods

### Timeline and key parts of the study

To drive delivery of the project, an international Executive Committee (EC) of multidisciplinary experts was formed, and an Independent Expert Panel (IEP) provided oversight and quality control assurances (see Additional file A1). Development of CONSORT-DEFINE commenced in March 2021, followed by SPIRIT-DEFINE in January 2022. The CONSORT-DEFINE protocol was deposited on the EQUATOR network in November 2021, followed by the SPIRIT-DEFINE Protocol in May 2022 [11, 12]. The consensus meeting took place in October 2022.

### Literature review and draft checklist generation

The initial generated list of CONSORT-DEFINE candidate items was informed by a methodological review of EPDF trial reports published between 2011 and 2020 to assess their reporting quality [9, 13], based broadly on existing reporting guidelines or recommendations, including CONSORT 2010 [4], SPIRIT 2013 [8], adaptive designs CONSORT extension (ACE) [14], and a proposal for a checklist for phase I dose-finding cancer trials [15], as well as consultation with experts, as described [9, 13].

The draft CONSORT-DEFINE list was further enriched via a review of the published and grey literature, real-world examples analysis, citation tracking, and experts’ recommendations as follows [10]. We conducted two searches on the MEDLINE and EMBASE databases on June 18 and September 7, 2021, respectively, to identify published EPDF literature for CONSORT-DEFINE (see Additional file A2). The following information was extracted from the included articles: (1) potential new or modified candidate items, (2) suggested content for the explanation and elaboration of candidate items, (3) confirmation of already identified items, and (4) general comments.

The resulting draft checklist was externally reviewed by 11 multidisciplinary stakeholders, covering key categories under-represented in the DEFINE Executive Committee (trial management staff, non-oncology clinicians, research ethics committees, journal editors, funders, and patient and public involvement (PPIE) representatives). The trial management staff category was covered via a call for volunteers circulated through the UK Clinical Research Collaboration Trial Managers Network (UKCRC TMN). The reviewers were asked to provide a high-level review of the draft checklist content to include any modifications or suggested additions.

An initial draft of the SPIRIT-DEFINE checklist was prepared, building on SPIRIT 2013 [8] and the draft candidate items identified from the CONSORT-DEFINE development work. Two independent searches were conducted in PubMed for relevant published literature on January 17 and March 17, 2022 (see Additional file 3). We also contacted funding bodies, regulatory agencies, research ethics committee, pharmaceutical companies, contract research organisations (CROs), research institutes/hospitals, Medicines and Healthcare products Regulatory Agency (MHRA)-accredited phase I units, and professional association/consortium to see if they adopted or used a protocol template, guideline, or checklist to write or review EPDF protocols that they were willing to share (see Additional file 3). EPDF trial protocol templates that were freely available on the internet were also examined.

### The Delphi process

#### General principles and scoring system

**Fig. 1:**
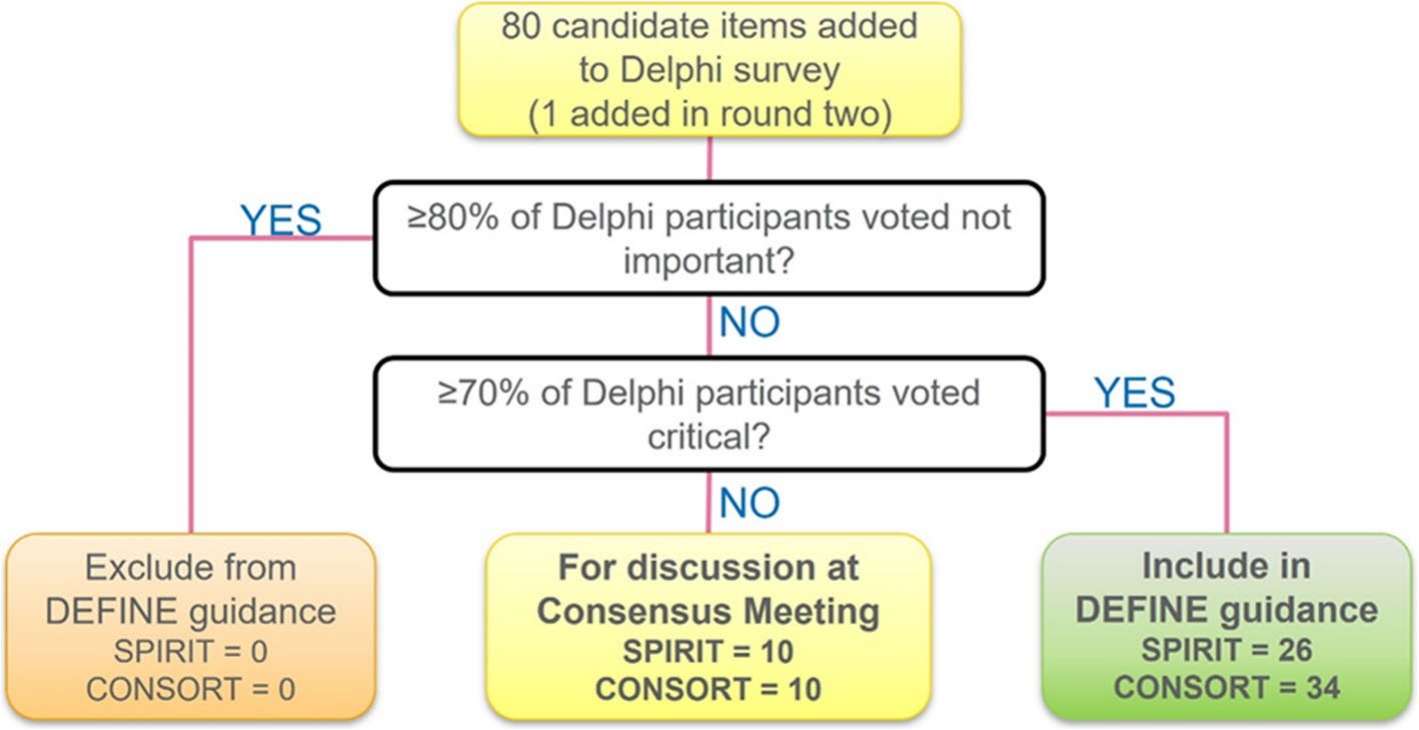
Criteria for dropping items between Delphi survey rounds as well as automatic inclusion in checklists

The draft candidate items for SPIRIT-DEFINE and CONSORT-DEFINE checklists were submitted for consultation and feedback to a wide stakeholder group through a Delphi survey. The Delphi process was conducted according to existing methodological guidance [16–18] and involved inviting participants to score the importance of the candidate items from the draft checklists through two iterative rounds of a web-based survey using DelphiManager, hosted by the University of Liverpool [19]. An importance rating scale of 1 to 9 was used: “not important” (score 1–3), “important but not critical” (score 4–6), “critically important” (score 7–9), and “unable to rate.” The thresholds for dropping items between rounds as well as automatic inclusion in the checklists were pre-specified (Fig. 1).

Prior to the launch of the Delphi survey, pilot rounds were conducted by the Executive Committee to fine-tune the draft checklists, troubleshoot any issues, and confirm the survey platform’s flow and functionality. Additionally, as part of the pilot run, we sent SPIRIT-DEFINE and CONSORT-DEFINE draft checklists to three experienced PPIE representatives to assess if they felt that patient representatives would be able to participate in the rating of the candidate items.

To ensure that the Delphi survey participation reflected the landscape of EPDF trials, key multidisciplinary stakeholder categories, including clinical trial researchers, regulators, ethics committees, journal editors, funders and patient and the public, were defined (see Additional file 4) [10]. Potential participants in EPDF trials were provided with participant information sheets and invited via email to participate in the Delphi survey. They were identified through a wide range of platforms, including targeted mailing lists of professional groups (including UK Experimental Cancer Medicine Centres, UKCRC Registered CTU Network, Adaptive Design Working Group and Statistical Analysis Working Group of MRC-NIHR Trial Methodology Research Partnership, NIHR Statistics Group and International Early Phase Adaptive Trials Workshop participants) and social media (Twitter and LinkedIn). We also invited stakeholders from academia and industry, either found online or recommended by the DEFINE Executive Committee and IEP. Invites were also sent to journal editors and corresponding authors collated from our reviews in Literature review and draft checklist generation section. Moreover, members of the MHRA-accredited phase I units were identified and invited. Individuals recommended by experts, including pharmaceutical company employees and PPI representatives, were also invited. Participants provided informed consent, and demographics, professional characteristics, and country information were collected. Registration rates were monitored continually, with the aim for at least 15 participants in each stakeholder category. Round One took place from March 28 to May 5, 2022, and Round Two from May 30 to June 27, 2022. Registration for Round One was necessary to take part in Round Two.

#### Analysis

##### Quantitative analysis

The number of invitation emails sent to potential participants who registered and responded to the survey in each round were presented in a flow diagram. Categorical baseline characteristics were summarised using frequency and percentage and continuous data using median and interquartile range (IQR) for all those who registered and the subsets who responded. The level of agreement between rounds was measured using percentage agreement (the percentage of participants with the same rating between rounds relative to the total responders to all rounds) and weighted Cohen’s kappa coefficient using absolute error weights [20].

Further details of the statistical methods for quantitative analyses are provided in Additional file 5 [20–25].

##### Qualitative analysis

An inductive thematic analysis using a semantic approach was performed on all free-text comments [26]. Open-ended feedback was extracted from the survey, collated, and uploaded for analysis using NVivo v1.6.2 software; the analysis was conducted by one assessor (SH) and reviewed by the DEFINE research team. After data familiarisation, initial codes were generated for each comment. More than one code could be assigned to a single comment. Initial codes were subsequently grouped into higher-level themes.

Comments received per item were grouped based on whether the item was for CONSORT-DEFINE or SPIRIT-DEFINE. Comments were manually summarised by two assessors (DP and OS) according to the number of comments received, the number of participants commenting on the item, the content (the theme) of the comment, and the number of times the theme was repeated for that item. The analysis was conducted independently but was confirmed by all assessors.

### Consensus meeting

Following the Delphi survey, a consensus meeting was convened on October 11 and 12, 2022, to finalise the full list of items to be included in the guideline, guided by the information on item importance and level of agreement from the Delphi survey, as well as examples of their use in trial protocols or trial reports. The consensus meeting followed the recommended methodology for such an exercise [27]. International experts in each of the relevant stakeholder categories were invited, ensuring a balance of representation across the categories (see Additional file 4). Results from the consensus meeting and the final checklists will be covered in subsequent DEFINE-SPIRIT and DEFINE-CONSORT statement papers.

## Results

### Literature review and draft checklist generation

Data were extracted from 476 randomly selected dose-finding trials published between 2011 and 2020 using the bibliographic database MEDLINE (via PubMed), stratified by oncology (*n* = 238) and non-oncology (*n* = 238) settings [13]. The findings of the review revealed inconsistent and inadequate reporting of EPDF trials were reported previously. Several items related to EPDF trial aspects were poorly reported, with notable differences between oncology and non-oncology settings. Notably, very few trials provided an accessible protocol (6.3%), statistical analysis plan (3.8%), or lay summary (1.5%). Examples of key items that were poorly reported include specification of planned/maximum sample size, and with justification; definition of the analysis population; and rationale for starting dose [9]. Improvement in trial reporting over time was evident in only a few items, including a marked rise in the use of participant flow diagram (from 15.4% in 2011 to 60.7% in 2020) and a modest increase in sample size justification (from 17.9% in 2011 to 25.0% in 2020).

To identify literature with guidance on writing protocols and reporting of early phase dose-finding trials, we conducted separate searches, as detailed in Additional file 2. Starting with an initial 5291 hits for CONSORT-DEFINE, the articles were screened for duplicates and relevance before being assessed by 11 experts, yielding 47 included articles plus an additional 12 based on the experts’ recommendations. The first search for SPIRIT-DEFINE literature had 265 article hits, and the second search with broader search terms gained 6741 articles, which after screening and assessment yielded 57 included articles, 9 of which were also included in the CONSORT-DEFINE literature search result. Additional file 6 [2–4, 7, 8, 28–124] lists the relevant articles and their allocation to potential candidate items.

When contacted for protocol templates or guidance for EPDF trials, 29 out of 49 (59.2%) organisations responded (see Additional file 3). Amongst those who responded, none of the 8 funding bodies adopted or recommended any EPDF protocol template, while 2 out of 4 pharmaceutical companies and CROs that responded had EPDF templates but could not share them. Thirteen professional organisations, research institutes/hospitals, and MHRA-accredited phase I units responded, with three sharing their protocol template (one EPDF template and two non-EPDF templates). The protocol templates were informative in structuring potential checklist items in an EPDF protocol and in developing some of the wording for Delphi survey items.

For CONSORT-DEFINE, 22 new items were identified as being relevant to the reporting of the EPDF trials (21 for main report and 1 for abstract) and 21 items (20 for main report and 1 for abstract) were modified and expanded to reflect their unique features. Thus, 43 CONSORT-DEFINE candidate items were included in the Delphi survey and sent for a pre-survey review. Six external reviewers provided feedback to help refine the wording and explanation of some candidate items, such as the dose allocation method, which was detailed by specifying the sequence and interval between dosing of participants, e.g. sentinel or staggered dosing; it was also highlighted that there might be some overlap with the CONSORT extension for randomised pilot and feasibility trials [125].

For SPIRIT-DEFINE, 20 new candidate items were identified from all the included literature and 16 of the original SPIRIT items were modified and expanded to encompass the features of EPDF trials. As a result, a total of 36 SPIRIT-DEFINE candidate items were included in the Delphi survey.

### Delphi survey

As part of the draft checklists’ pilot testing (General principles and scoring system section), the PPIE representatives advised that the candidate items were too complex and technical for patients or the general public to fully participate in the Delphi survey. To address this, the Executive Committee decided that instead of circulating the DEFINE Delphi survey directly to PPIE platforms, we would instead use a snowballing approach targeting experienced patient partners in clinical trials and asking them to circulate to their experienced PPIE contacts. This feedback also triggered the formation of the protocol-specified PPIE working group to discuss how best to embed the perspectives of patients as key stakeholders to encourage better reporting of EPDF trials and to facilitate the dissemination of the resulting SPIRIT-DEFINE and CONSORT-DEFINE guidelines (further details in Lay summary toolkit secion and Additional file 7) [1, 126–138].

#### Response rates across rounds

**Fig. 2:**
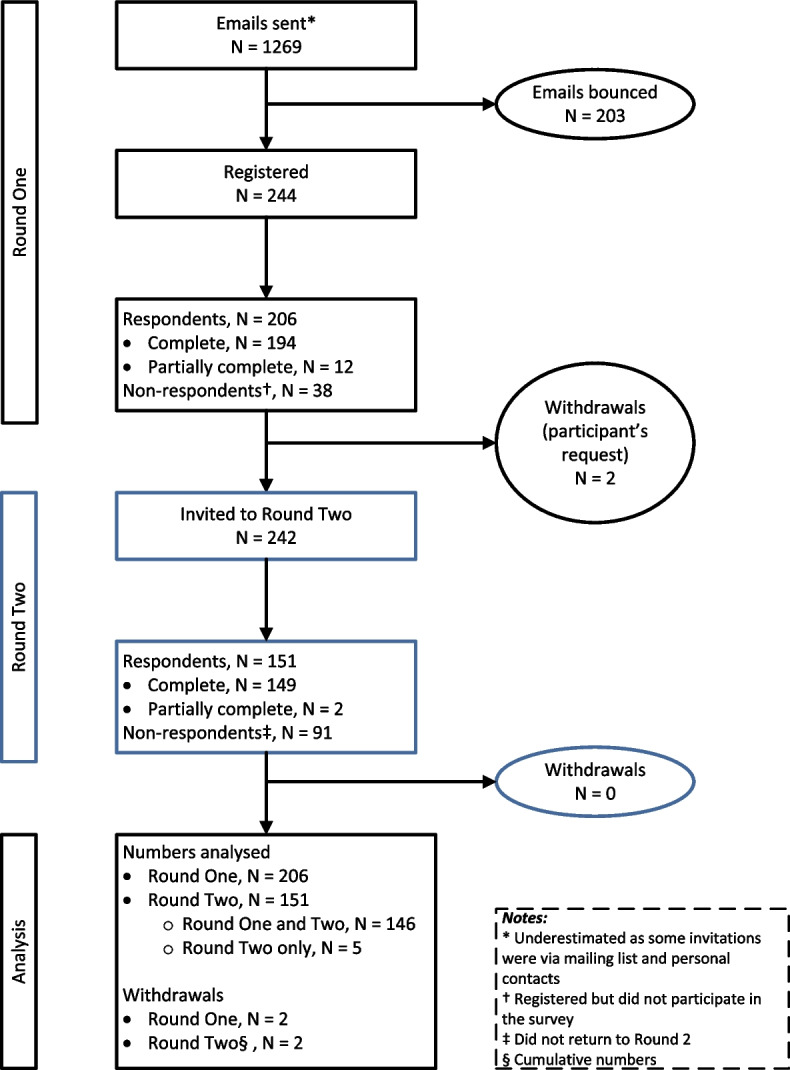
Flow chart of the DEFINE Delphi survey

Figure 2 shows the flow of participants who were approached, registered, and responded to the Delphi surveys. During Round One of the Delphi survey, we reviewed the enrolment progress regularly, and we discovered that a large proportion of registered participants were based in Western Europe and North America. A targeted continental search for early phase trials on www.clinicaltrials.gov was conducted to ensure we captured perspectives of stakeholders from wide geographical regions. Additional designated trial contacts located in Eastern Europe, Asia, Africa, and South America were obtained via ClinicalTrials.gov. Out of the 73 additional stakeholders who were invited to participate in the Delphi survey, only 2 registered and 1 participated. A total of 206 participants responded to Round One of the Delphi survey and 151 to Round Two.

As part of our pre-planned sensitivity analysis, we carried over the ratings of 59 respondents from Round One to Round Two for the Round One participants who did not take part in Round Two.

#### Characteristics of registered participants and respondents

The Delphi survey included a wide range of stakeholders, with the majority identifying as statisticians, trial methodologists, data scientists, or quantitative analysts (47.1%) and clinicians or clinical pharmacologists (32.4%) (see Additional file 8, Table A8-1). Most respondents were from academic organisations (77% in Round One). Based on the free-text field provided, participants who responded with “Other” to their experience in early phase trial roles were reclassified as one of the roles/types in the prespecified list (see Additional file 8, Table A8-2). Respondent demographics were relatively consistent across both rounds, with participants from five continents and 24 countries in Round One; most participants were from Europe (62.1%) followed by North America (25.7%) (see Additional file 8, Table A8-1).

#### Perceptions of proposed candidate items

In Round One, 52 of the 79 candidate items (SPIRIT-DEFINE, *n* = 25, and CONSORT-DEFINE, *n* = 27) had at least 70% of respondents rating them as “critically important” (scores 7 to 9). There was no item with 80% or more respondents scoring it as “not important” (scores 1 to 3). Therefore, none was removed after Round One.

Suggestions for new items from participants in Round One were considered by the Executive Committee, and one suggested item, “Access (or link) to code/functions used for simulation studies,” was added to CONSORT-DEFINE. Other suggested items were either already covered by existing items or were not specific to EPDF trials.

**Fig. 3:**
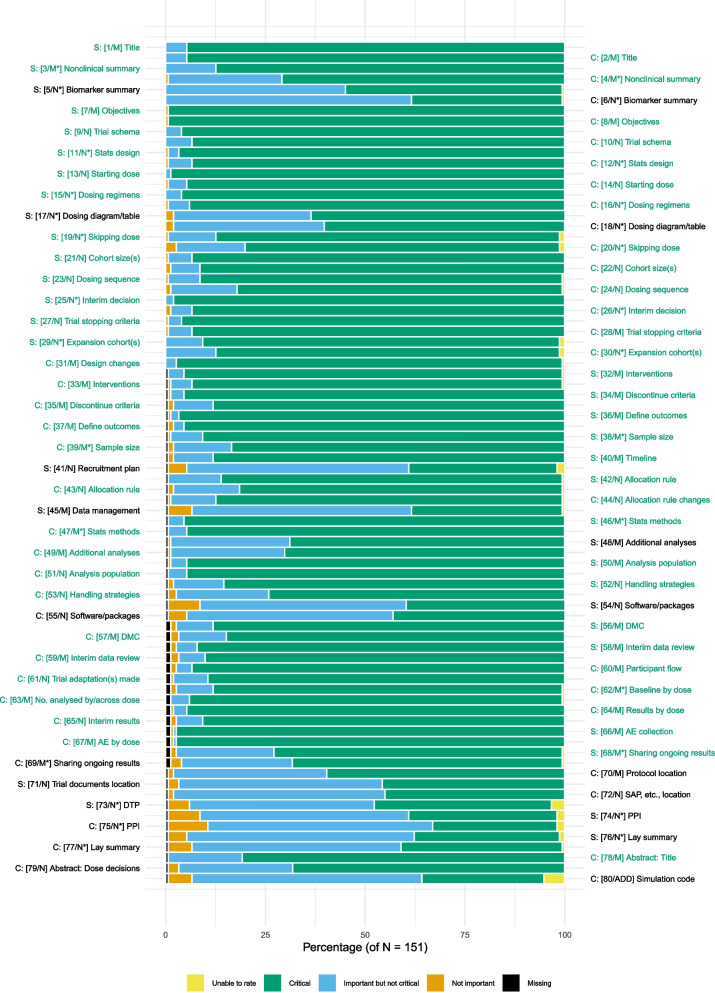
Bar plot of the percentage of respondents scoring each item in Round Two. Items in green text had at least 70% of respondents scoring them as “critically important” (scores 7 to 9)

In Round Two, 60 of 80 candidate items had at least 70% of respondents rating them as “critically important” (scores 7 to 9) (Fig. 3). All 52 items in Round One that met the inclusion threshold remained highly scored in Round Two.

In our assessment of the stability and consistency of individual ratings of item importance across rounds, we found a reasonably high level of individual agreement. Twenty items had moderate agreement (0.41 to 0.6) and 59 had substantial agreement (0.61 to 0.8). This suggested that respondents had “converged” in their responses (see Additional file 9, Table A9-3).

Visual inspection of the item rating by early phase roles showed that there was no difference for most items except for five candidate items (SPIRIT-DEFINE: planned dosing regimens, additional statistical analyses, location of the protocol, and support for the planned biomarker sub-study; CONSORT-DEFINE: planned dosing regimens, presentation of interim results (see Additional file 9, Figures A9-3 to A9-7)). All these items were also rated as critical by more respondents from Asia than from Europe and North America (see Additional file 9, Figures A9-8 to A9-12).

Detailed analysis of the respondents’ perceptions of the importance of the candidate items and the sensitivity analysis are provided in Additional file 9, Table A9-1.

#### Qualitative analysis based on open-ended feedback from participants

Following the methodology defined in Analysis section, general comments from Round One were coded and grouped into six higher-level themes: content consideration, feedback on experience of Delphi process, guideline structure, trial participant characteristics, patient and public involvement, and unrelated content (see Additional file 11). The feedback was used to further refine candidate items to improve their applicability and as background information for the consensus meeting discussions.

#### Inclusion of candidate items in DEFINE checklists

At the end of the Delphi survey, 60 items were automatically recommended for inclusion based on the pre-specified inclusion threshold (Fig. 1). The remaining 20 items (SPIRIT-DEFINE, *n* = 10, and CONSORT-DEFINE, *m* = 10) were tabled for discussion at the DEFINE consensus meeting (see Additional file 10).

#### Expert Focus Group with expertise in non-oncology and healthy volunteers trials

In response to feedback that some candidate items were oncology-focused, after Round Two, we conducted a targeted engagement step via an Expert Focus Group of five international stakeholders with expertise in healthy volunteers and non-oncology EPDF trials to seek their views on the candidate items and identify if refinements to the item description may be required to facilitate their applicability in a non-oncology context. Based on that discussion, some terminologies were changed to ones with broader applicability, such as “dose-limiting toxicity” to “safety measures”; item explanations were enriched to be applicable in all settings (including first-in-human studies), and optionality, i.e. “where applicable,” was added in some cases.

### Lay summary toolkit

As described in Delphi survey section, a PPIE working group was formed, consisting of six representatives from both the oncology and non-oncology fields, as well as those with experience in early phase trials. The perspectives of patients and participants on the reporting of EPDF trials were explored. The discussion highlighted the need to develop an easy-to-understand lay summary of the scientific publication of EPDF trials in order to provide increased transparency for patients and the general public. It was also suggested that the provision of a good lay summary exemplar of a published EPDF trial would be helpful to facilitate implementation. This led to the co-development of a lay summary toolkit, which includes a template with guidance notes and an exemplar for the reporting of EPDF trials, taking into consideration the CONSORT-DEFINE candidate items. The drafted template, guidance, and exemplar were then sent for review to three professional experts (a communications media manager, an ethics committee member, and a regulator) to gather feedback and further refinement before a wider consultation with the patient and public involvement and engagement in musculoskeletal research (PIMS) group. The resultant lay summary toolkit provides recommendations of key information that should be reported from the perspective of both patients and the general public (see Additional file 7).

## Discussion

The prevalence and importance of early phase trials in the clinical development pipeline, as well as the increasing complexity of the designs used, necessitate thorough, precise, and transparent efforts to ensure that the work can be accurately evaluated and, if necessary, reproduced. The methodological review findings of inconsistent and inadequate reporting of important methodological features in design, conduct, and analysis confirm the need for robust, consensus-driven extensions of SPIRIT 2013 [139] and CONSORT 2010 [9] to provide the research community with the tools needed to accurately report and assess EPDF trials while meeting the standards achieved by the original checklists [4, 8] and existing extensions in the broader clinical trials reporting landscape [140].

SPIRIT-DEFINE and CONSORT-DEFINE were developed using gold-standard methodology to provide robust international evidence and consensus-based guidance. Concurrent development has enabled us to harmonise both guidelines while also achieving increased clarity and a broader perspective on the items required for either or both. This will make it easier for researchers to write the trial protocol and trial report, as well as enable reviewers to assess the final report’s adherence to the protocol as per other recent joint SPIRIT and CONSORT extensions, such as for outcomes reporting [141].

Not many reporting guidelines have provided detailed descriptions of their development processes and results (ACE [14] is one of the exceptions), and we hope that this paper will assist readers in understanding the “DEFINE development journey” as well as enable researchers planning to develop future guidelines or extensions to learn from our experience.

### Main strengths

This project’s main strengths were its solid methodological foundations and broad participation. The development of the guidelines was supported by independent oversight and was underpinned by detailed protocols that pre-specified the methods [10].

Strong PPIE engagement was achieved throughout the process, with the co-development of a lay summary toolkit (consisting of a user-friendly template with guidance notes and an exemplar) with our PPIE partners. To the best of our knowledge, this is the first time that a reporting guideline development has embedded within it the production of a toolkit to aid researchers in providing a lay summary of their trial results more effectively to patients and the public. We hope this will encourage future guideline developers to consider this to facilitate implementation. Close engagement will be maintained throughout the dissemination stages.

The comprehensive methodological review included 476 randomly selected trial reports, which were supplemented by subsequent literature reviews.

From the beginning of the process, the DEFINE group sought to engage multiple organisations, both academic and commercial, to ensure that the resulting guidelines would meet the needs of the entire EPDF community. The Delphi survey was successful in involving 206 delegates from 5 continents and 24 countries in a variety of job roles in EPDF trials, exceeding the participation target. It also achieved a high level of consensus across the two rounds, with 60 items meeting the pre-defined threshold.

The dissemination strategy aims to maximise guideline awareness and uptake, including but not limited to dissemination in stakeholder meetings, conferences, peer-reviewed publications, and on the EQUATOR Network and DEFINE project websites.

### Main limitations

Despite the above-mentioned key strengths, we encountered several limitations. A combination of approaches was employed to distribute the survey invitations. These included advertising on social media, sending invitations to targeted mailing lists of professional groups, corresponding authors from a random selection of trials, and pre-selected individuals with expertise in the field. For mailing lists of larger groups, as participants were self-selected, the survey results might have been influenced by non-response bias, and we were unable to determine the profile of those who did not sign up to participate. The approach of pre-selecting specific individuals might have introduced certain biases, such as selection bias and sampling bias. Nevertheless, the combination of circulating surveys to larger groups and pre-selected individuals ensured a balanced blend of inclusivity and targeted engagement, maximising the diversity of participants while levering the expertise of key stakeholders, ultimately yielding valuable contributions.

Given that the survey had 80 candidate items and was expected to take around 30 min to complete, 29% of Round One respondents did not return to Round Two, which could be attributed to participant fatigue. To address this, items relevant to both guidelines were presented together, with participants able to save and return later.

Despite the distribution of respondents' characteristics being consistent with the landscape of EPDF clinical trials, the majority of respondents came from academic organisations. Some geographical regions outside of Western Europe and North America were underrepresented, and targeted efforts were made to recruit more participants from these areas. Finally, after Round One, participants were unable to provide feedback on individual items but could provide general comments.

## Conclusions

By implementing a robust, comprehensive gold-standard methodological framework for guideline development, SPIRIT-DEFINE and CONSORT-DEFINE will allow investigators to effectively address the essential items that should be included in trial protocols and reporting, thus promoting transparency, completeness, and reproducibility of methods. SPIRIT-DEFINE and CONSORT-DEFINE will also provide a framework for peer review of EPDF trial protocols and reports, including an assessment of the quality of the trial design and methods as well as the risk of bias in the reported outcomes.

By sharing the DEFINE development methods and the decisions undertaken with multi-stakeholder groups, including PPIE partners, we hope it will serve as a model to support future guideline development projects.

## Supplementary Information

**Additional file 1.** Composition of the DEFINE Executive Committee and Independent Expert Panel.

**Additional file 2.** CONSORT and SPIRIT DEFINE Literature review: **Figures A2-1 – A2-2**, **Tables A2-1.** **Figure A2-1.** PRISMA 2020 flow diagram for the CONSORT-DEFINE literature search and review. **Figure A2-2.** PRISMA flow diagram for the SPIRIT-DEFINE literature search and review. **Table A2-1.** Terms used in the PubMed searches and the number of hits from each search terms for SPIRIT-DEFINE.

**Additional file 3.** Organisations contacted for protocol templates or guidelines and responses.

**Additional file 4.** Delphi Survey key stakeholder groups and methods of access.

**Additional file 5.** Further details on the statistical analysis plan.

**Additional file 6.** SPIRIT-DEFINE and CONSORT-DEFINE items generation for Delphi survey: **Table A6-1.** **Table A6-1.** References supporting the proposed candidate items in the DEFINE Delphi survey.

**Additional file 7.** Toolkit for Lay Summary of EPDF Trial Results.

**Additional file 8.** Delphi survey participants demographics and harmonisation of roles: **Tables A8-1** – **A8-2.** **Table A8-1.** Demographics of Delphi survey participants. **Table A8-2.** Harmonisation of participants’ defined roles.

**Additional file 9.** Perception of proposed items and sensitivity analysis: **Tables A9-1 – A9-3**, **Figures A9-1** – **A9-13.** **Table A9-1.** Summary of categorical and numerical ratings at Round One and 2. **Figure A9-1.** Percentage of participants changing their numerical scores at Round Two. **Figure A9-2.** Percentage of participants changing their categorical scores at Round Two. **Table A9-2.** Number of participants changing their ratings between Round One and Round Two. **Table A9-3.** Frequency and percentage of perfect agreement. **Figure A9-3.** Stacked bar plots of candidate item [17 SPIRIT-DEFINE] “Planned dosing regimens presented as a diagram or table, where applicable” by stakeholders. **Figure A9-4.** Stacked bar plots of candidate item [18 CONSORT-DEFINE] “Planned and delivered dosing regimens presented as a diagram or table, where applicable” by stakeholders. **Figure A9-5.** Stacked bar plots of candidate item [48 SPIRIT-DEFINE] “Statistical methods for additional analyses” by stakeholders. **Figure A9-6.** Stacked bar plots of candidate item [69 CONSORT-DEFINE] “Specify if and when results were reported whilst the trial was still ongoing” by stakeholders. **Figure A9-7.** Stacked bar plots of candidate item [70 SPIRIT-DEFINE] “Where the full trial protocol or the redacted version, with amendments, can be accessed” by stakeholders. **Figure A9-8.** Stacked bar plots of candidate item [17 SPIRIT-DEFINE] “Planned dosing regimens presented as a diagram or table, where applicable” by Continent. **Figure A9-9.** Stacked bar plots of candidate item [18 CONSORT-DEFINE] “Planned and delivered dosing regimens presented as a diagram or table, where applicable” by Continent. **Figure A9-10.** Stacked bar plots of candidate item [48 SPIRIT-DEFINE] “Statistical methods for additional analyses” by Continent. **Figure A9-11.** Stacked bar plots of candidate item [69 CONSORT-DEFINE] “Specify if and when results were reported whilst the trial was still ongoing” by Continent. **Figure A9-12.** Stacked bar plots of candidate item [70 SPIRIT-DEFINE] “Where the full trial protocol or the redacted version, with amendments, can be accessed” by Continent. **Figure A9-13.** Stacked bar plots of candidate item [5 SPIRIT-DEFINE] “Summary of findings from existing correlative biomarker, correlative and associated studies to support planned biomarker sub-study” by Continent.

**Additional file 10.** List of candidate items to be discussed at the DEFINE Consensus meeting.

**Additional file 11.** Qualitative analysis. **Table A11-1**; **Figure A11-1.** **Table A11-1.** Themes and codes identified in the general comments received in Delphi survey. **Figure A11-1.** Tree diagram of the comments received per theme.

## Data Availability

Data is shared under a data transfer agreement or a collaboration agreement depending on the nature of the sharing. Requests are via a standard proforma describing the nature of the proposed research and extent of data requirements.

## Abbreviations

ACE: Adaptive designs CONSORT extension
CONSORT: Consolidated Standards of Reporting Trials
CONSORT-DEFINE: CONSORT extension for early phase dose-finding trials
CRO: Clinical Research Organisation
DEFINE: Dose-finding extensions
EC: Executive committee
EMA: European Medicines Agency
EPDF: Early phase dose-finding
EQUATOR: Enhancing the QUAlity and Transparency Of health Research
IEP: Independent Expert Panel
IQR: Interquartile range
MHRA: Medicines and Healthcare products Regulatory Agency
NCRI: National Cancer Research Institute
PPIE: Patient and public involvement and engagement
SPIRIT: Standard Protocol Items: Recommendations for Interventional Trials
SPIRIT-DEFINE: SPIRIT extension for early phase dose-finding trials
