# Supplementary material for: Development of consensus-driven SPIRIT and CONSORT extensions for early phase dose-finding trials: the DEFINE study
Source: BMC Med. 2023 Jul 5;21:246. doi: 10.1186/s12916-023-02937-0 (PMC10324137; doi:10.1186/s12916-023-02937-0)
Supplement: Supplementary file 1 — Additional file 1. Composition of the DEFINE Executive Committee and Independent Expert Panel. [file 12916_2023_2937_MOESM1_ESM.docx]

# Composition of the DEFINE Executive Committee and Independent Expert Panel

Executive Committee

Christina Yap (Principal Investigator), Institute of Cancer Research, UK; Munyaradzi Dimairo, University of Sheffield, UK; Christopher Weir, University of Edinburgh, UK; Adrian Mander, GlaxoSmithKline, UK; Thomas Jaki, University of Cambridge, UK; Jeff Evans, University of Glasgow, UK; Rong Liu, Bristol-Myers Squibb, US; Shing Lee, Columbia University, US; Andrew Kightley (DEFINE PPIE Lead), Tarsius, UK; Sally Hopewell, University of Oxford, UK; Johann de Bono, Institute of Cancer Research, UK; Alun Bedding, Roche, UK.

Collaborators/Advisors

Khadija Rantell, Medicines and Healthcare products Regulatory Agency, UK; Moreno Ursino, Université de Paris, France; John Kirkpatrick, Roche, UK; Stephen Hahn, Flagship Pioneering, US.

Independent Expert Panel

Elizabeth Garret-Mayer (chair), American Society of Clinical Oncology, US; Deborah Ashby, Imperial College London, UK; John Isaacs, Newcastle University, UK; and Melanie Calvert, University of Birmingham, UK.
