# Supplementary material for: Development of consensus-driven SPIRIT and CONSORT extensions for early phase dose-finding trials: the DEFINE study
Source: BMC Med. 2023 Jul 5;21:246. doi: 10.1186/s12916-023-02937-0 (PMC10324137; doi:10.1186/s12916-023-02937-0)
Supplement: Supplementary file 2 — Additional file 2. CONSORT and SPIRIT DEFINE Literature review: Figures A2-1 – A2-2, Tables A2-1. Figure A2-1. PRISMA 2020 flow diagram for the CONSORT-DEFINE literature search and review. Figure A2-2. PRISMA flow diagram for the SPIRIT-DEFINE literature search and review. Table A2-1. Terms used in the PubMed searches and the number of hits from each search terms for SPIRIT-DEFINE. [file 12916_2023_2937_MOESM2_ESM.docx]

# CONSORT and SPIRIT-DEFINE Literature Review

CONSORT-DEFINE

To identify literature with guidance on reporting of EPDF trials, we conducted two searches. The first search was conducted on the MEDLINE database via PubMed on June 18, 2021:

("dose escalat*"[Title/Abstract] OR "dose find*"[Title/Abstract] OR "dose expan*"[Title/Abstract] OR "single ascending dose"[Title/Abstract] OR "multiple ascending dose"[Title/Abstract] OR "first in man"[Title/Abstract] OR "first in human"[Title/Abstract] OR "early phase"[Title/Abstract] OR "phase 1a"[Title/Abstract] OR "phase 1b"[Title/Abstract] OR "phase ia"[Title/Abstract] OR "phase ib"[Title/Abstract] OR "phase 1-2"[Title/Abstract] OR "phase i-ii"[Title/Abstract] OR "MTD"[Title/Abstract] OR "dose-titration"[Title/Abstract]) AND ("reporting"[Title/Abstract] OR "recommendation*"[Title/Abstract] OR "best practice"[Title/Abstract] OR "good practice"[Title/Abstract] OR "panel discussion*"[Title/Abstract] OR "guidance"[Title/Abstract] OR "guideline*"[Title/Abstract] OR "expert opinion*"[Title/Abstract] OR "interpretation"[Title/Abstract]).

On September 7, 2021, we repeated the PubMed search, but this time using MeSH terms, as well as EMBASE was searched via the Healthcare Databases Advanced Search National Institute of Health and Care Excellence (HDAS NICE):

(("clinical trials, phase i as topic"[MeSH Terms] OR "clinical trials, phase ii as topic"[MeSH Terms] OR "Maximum Tolerated Dose"[MeSH Terms] OR "Clinical Trials Data Monitoring Committees"[MeSH Terms]) AND ("reporting"[Title/Abstract] OR "recommendation*"[Title/Abstract] OR "best practice"[Title/Abstract] OR "good practice"[Title/Abstract] OR "panel discussion*"[Title/Abstract] OR "guidance"[Title/Abstract] OR "guideline*"[Title/Abstract] OR "expert opinion*"[Title/Abstract] OR "interpretation"[Title/Abstract]) AND "humans"[MeSH Terms]) AND ((humans[Filter]) AND (english[Filter]))

The searches yielded 5,291 articles, 548 of which were deleted as duplicates. Two reviewers independently screened the titles and, if necessary, abstracts of the remaining 4,743 articles, and 4,412 records were excluded as irrelevant. One reviewer further screened the full texts of the remaining 331 articles, and only 107 were recommended for eligibility assessment. Nine experts from the Executive Committee and two reviewers evaluated the articles, and another 60 were rejected as irrelevant. Thus, 47 articles retrieved from databases were identified as relevant. Twelve regulatory, guidance, and recommendation documents related to EPDF trials were recommended by regulatory and professional body experts (Figure A2-1).


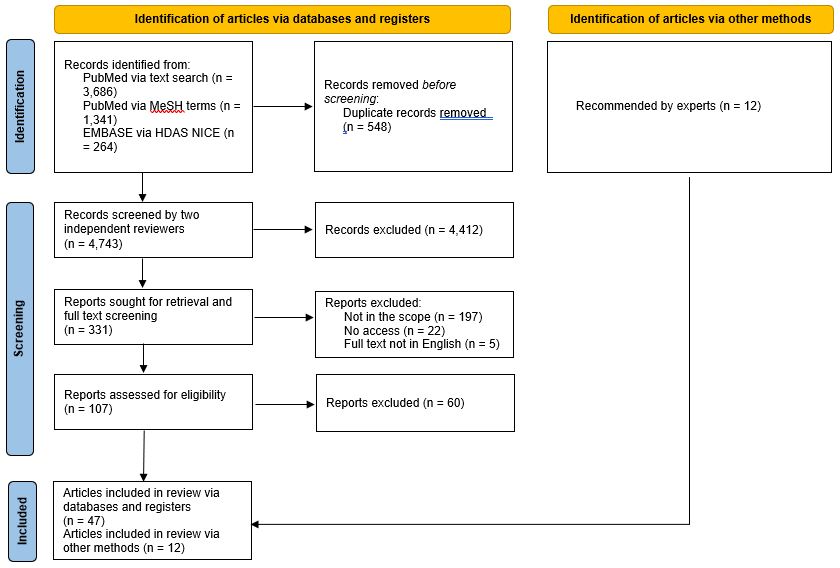


*Figure A2-1: PRISMA 2020 flow diagram for the CONSORT-DEFINE literature search and review.*

SPIRIT-DEFINE

Two independent searches were conducted in PubMed for published literature on EPDF protocol guidance. On January 17, 2022, the first search was conducted using a modified version of the CONSORT-DEFINE search. On March 17, 2022, a second search was conducted using broader search terms.

The first search used the same search terms as the CONSORT-DEFINE search but with additional “AND ("protocol"[Title/Abstract] OR "SPIRIT"[Title/Abstract])) AND ((fft[Filter]) AND (english[Filter])”. Table S2-1 shows the terms used in the search and the number of hits for the corresponding search terms. Figure S2-2 shows the PRISMA flow diagram of the number of articles searched, reviewed, and assessed. There were 265 hits, and only nine (3.4%) articles were retrieved for assessment. Any discrepancies were resolved by discussion and consensus. All were included to generate candidate items for the SPIRIT-DEFINE checklist. We performed backward and forward searches on the first search, and nine articles were retrieved for eligibility. A further 39% (24/61) peer-reviewed articles, book chapters and grey literature from the backward-forward search and recommendation by experts were included to draft the SPIRIT-DEFINE checklist.

The second independent search used broader search terms (Table A2-1) and 102/6741 (1.5%) articles were retrieved for assessment. Of these, 34 were included (Figure A2-2) but as 10 were duplicates from the first search, there were 57 unique articles included. Articles from the second search did not add or modify any further candidate items but provided additional content for the candidate items (including further details on what should be included and why they are important). Nine of the 57 unique articles in SPIRIT-DEFINE were also included in CONSORT-DEFINE.

*Table A2-1: Terms used in the PubMed searches and the number of hits from each search terms for SPIRIT-DEFINE*

| First search on 17 January 2022 | | | Second search on 17 March 2022 | | | |
| --- | --- | --- | --- | --- | --- | --- |
| Search number | **Search terms** | **Number of hits** | **Search number** | **Search terms** | **Number of hits** |  |
| #1 | "dose escalat*" OR "dose find*" OR "dose expan*" | 18,110 | #1 | "phase I" OR "phase Ia" OR "phase Ib" | 47,898 |  |
| #2 | "single ascending dose" OR "multiple ascending dose" | 624 | #2 | "phase 1" OR "phase 1a" OR "phase 1b" | 19,758 |  |
| #3 | "first in man" OR "first in human" | 3,994 | #3 | "phase I/II" OR "phase 1/2" | 9,179 |  |
| #4 | "early phase" OR "phase 1a" OR "phase 1b" OR "phase ia" OR "phase ib" | 31,018 | #4 | "early phase" OR "expansion cohort" | 29,538 |  |
| #5 | "phase 1-2" OR "phase i-ii" | 9,133 | #5 | "dose rang*" OR "dose find*" OR "dose expan*" | 20,071 |  |
| #6 | "MTD" OR "dose-titration" | 8,515 | #6 | "dose escalat*" OR "dose de-escalat*" OR "dose ascending" OR "ascending dose" OR "dose titration" | 18,303 |  |
| #7 | "reporting" OR "recommendation*" | 516,542 | #7 | MTD OR "maximum tolerated dose" OR DLT OR "dose limiting toxicit*" | 17,485 |  |
| #8 | "best practice" OR "good practice" | 20,390 | #8 | "first-in-human" OR "first-in-man" | 4,063 |  |
| #9 | "panel discussion*" OR "guidance" OR "guideline*" OR "expert opinion*" OR "interpretation" | 793,688 | #9 | guid* OR checklist* OR perspective* OR reflect* OR report* OR issue* OR require* OR "panel discussion*" OR opinion* OR interpretation | 8,054,538 |  |
| #10 | "protocol" OR "SPIRIT" | 379,308 | #10 | "recommend*" OR "best practice" OR "good practice" | 780,191 |  |
| #11 | (#1 OR #2 OR #3 OR #4 OR #5 OR #6) | 65,896 | #11 | (#1 OR #2 OR #3 OR #4 OR #5 OR #6 OR #7 OR #8) | 133,355 |  |
| #12 | (#7 OR #8 OR #9) | 1,211,970 | #12 | (#9 OR #10) | 8,421,099 |  |
| #13 | (#11 AND #12) AND #10 | 276 | #13 | #11 AND #12 | 51,410 |  |
| #14 | #13 Filters: full text | 268 | #14 | #13 AND (protocol OR "SPIRIT") | 7,607 |  |
| #15 | #14 Filters: English | 265 | #15 | #14: Filters: English only | 7,414 |  |
|  |  |  | #16 | #14: Filters: English and full text | 6,741 |  |

*
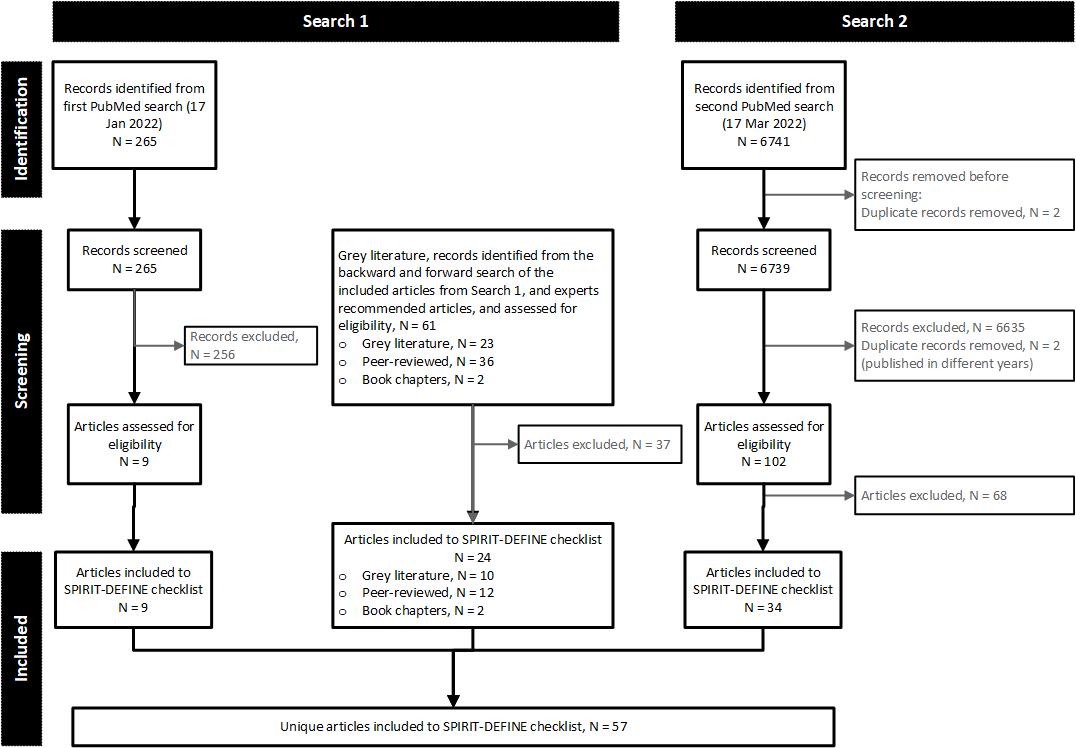
*

*Figure A2-2: PRISMA flow diagram for the SPIRIT-DEFINE literature search and review.*
