# Supplementary material for: Development of consensus-driven SPIRIT and CONSORT extensions for early phase dose-finding trials: the DEFINE study
Source: BMC Med. 2023 Jul 5;21:246. doi: 10.1186/s12916-023-02937-0 (PMC10324137; doi:10.1186/s12916-023-02937-0)
Supplement: Supplementary file 3 — Additional file 3. Organisations contacted for protocol templates or guidelines and responses. [file 12916_2023_2937_MOESM3_ESM.docx]

# Organisations contacted for protocol templates or guidelines and responses

| **Type** | **Name** | **Number responded (%)** |
| --- | --- | --- |
| Funder | 1. National Institute for Social Care and Health Research (NISCHR) | 8 (53%) |
|  | 1. NIHR Invention for Innovation (i4i) |  |
|  | 1. NIHR Efficacy and Mechanism Evaluation (EME) |  |
|  | 1. NIHR Programme Development Grant (PDG) and Programme Grants for Applied Research (PGfAR) |  |
|  | 1. Medical Research Council (MRC) DPFS |  |
|  | 1. National Cancer Institute of Canada Clinical Trials Group (NCIC) |  |
|  | 1. Cancer Research UK |  |
|  | 1. Multiple Sclerosis Society |  |
|  | 1. Arthritis Research UK |  |
|  | 1. Asthma UK |  |
|  | 1. Breast Cancer Now (formerly Breakthrough Breast Cancer) |  |
|  | 1. Breast Cancer Campaign |  |
|  | 1. Marie Curie Cancer Care |  |
|  | 1. Experimental Cancer Medicine Centres (ECMC) |  |
|  | 1. Blood Cancer UK |  |
| Regulators | 1. MHRA | 3 (75%) |
|  | 1. Federal Institute for Drugs and Medical Devices/ Bundesinstitut für Arzneimittel und Medizinprodukte (BfArM) (Germany) |  |
|  | 1. Health Canada |  |
|  | 1. Pharmaceuticals and Medical Devices Agency (PMDA) (Japan) |  |
| Research Ethics Committee | 1. UK Health Research Authority Research Ethics Committee | 1 (100%) |
| Pharmaceutical companies / Clinical Research Organisations | 1. Roche | 4 (40%) |
|  | 1. BMS |  |
|  | 1. Johnson & Johnson |  |
|  | 1. AstraZeneca |  |
|  | 1. Novartis |  |
|  | 1. Boehringer Ingelheim |  |
|  | 1. GSK |  |
|  | 1. Takeda Clinical Trials |  |
|  | 1. Pfizer |  |
|  | 1. Iqvia (Quintiles) |  |
| Research institutes/hospitals | 1. CRUK Centre for Drug Development | 9 (90%) |
|  | 1. National Cancer Centre Singapore |  |
|  | 1. Murdoch Children's Research Institute |  |
|  | 1. National Cancer Center Hospital (NCCH) Japan |  |
|  | 1. Chinese University of Hong Kong |  |
|  | 1. National Taiwan University Hospital |  |
|  | 1. Kenya Medical Research Institute (KEMRI)-Wellcome/Imperial College London |  |
|  | 1. Kenya Medical Research Institute (KEMRI)-Wellcome |  |
|  | 1. UCL |  |
|  | 1. Wellcome Mahidol Oxford Tropical Medicine Research Unit (MORU) |  |
| MHRA phase I units | 1. Labcorp Clinical Research Unit Limited (Leeds) (formerly known as Covance Clinical Research Unit (CRU)) | 2 (29%) |
|  | 1. Edinburgh Clinical Research Facility (Edinburgh) (WTCRF, RIECRF & CCRF) |  |
|  | 1. NIHR Guy’s & St Thomas Clinical Research Facility (Guy’s Unit) |  |
|  | 1. Quotient Sciences (Nottingham) |  |
|  | 1. Richmond Pharmacology (London) |  |
|  | 1. Royal Liverpool & Broadgreen University Hospital Trust Clinical Research Facility (RLBUH CRF) (Liverpool) |  |
|  | 1. Simbec-Orion (Merthyr Tydfil) (formerly known as Simbec Research Ltd) |  |
| Professional association/  consortium | 1. The Association of the British Pharmaceutical Industry (ABPI) | 2 (100%) |
|  | 1. Asian Oncology Early Phase 1 Consortium (AsiaOne) |  |
