# Supplementary material for: Development of consensus-driven SPIRIT and CONSORT extensions for early phase dose-finding trials: the DEFINE study
Source: BMC Med. 2023 Jul 5;21:246. doi: 10.1186/s12916-023-02937-0 (PMC10324137; doi:10.1186/s12916-023-02937-0)
Supplement: Supplementary file 4 — Additional file 4. Delphi Survey key stakeholder groups and methods of access. [file 12916_2023_2937_MOESM4_ESM.docx]

# Delphi survey key stakeholder groups and methods of access

| Stakeholders | Platforms |
| --- | --- |
| Clinical Trials Researchers (including  Clinicians/ Clinical Pharmacologists,  Trial management staff,  Statisticians,  Trial methodologists) | - Medical Research Council - National Institute for Health and Care Research Trial Methodology Research Partnership (MRC-NIHR TMRP) (UK) - UK Clinical Research Collaboration (UKCRC) Network of Registered clinical trial Units - Targeted conferences or organisations such as Society for Clinical Trials, International Clinical Trials Methodology Conference (ICTMC), International Society for Clinical Biostatistics (ISCB), Statisticians in the Pharmaceutical Industry (PSI), European Federation of Statisticians in the Pharmaceutical Industry (EFSPI), Drug Information Association (DIA) - Clinical Conferences such as the National Cancer Research Institute (NCRI) annual conference (NCRI), European Society for Medical Oncology (ESMO) congress, American Society for Clinical Oncology (ASCO), the Experimental Cancer Medicine Centres (ECMC) events, European Centre for Rare Diseases and orphan products (ECRD) - Sponsors from industry (via organisations such as Pharmaceutical Research and Manufacturers of America (PhRMA) in US, European Federation of Pharmaceutical Industries and Associations (EFPIA) in Europe) or the Association of British Pharmaceutical Industry (ABPI) - Publications (including corresponding authors of papers selected through the methodological review process) - Executive Committee members professional contacts - Targeted professional social network groups |
| Regulators | - US Food and Drug Administration (FDA) - European Medicines Agency (EMA) - UK Medicines and Healthcare products Regulatory Agency (MHRA), - Japan Pharmaceuticals and Medical Devices Agency (PMDA) - China National Medical Product Association Centre for Drug Evaluation (NMPA CDE) - Australia Therapeutic Group Administration (TGA) - Drugs Controller General of India (DCGI) - Health Products and Food Branch (HPFB), Health Canada - Ministry of Food and Drug Safety, South Korea - Executive Committee members professional contacts |
| Ethics Committee / Ethics Committee members | - UK Health Research Authority (HRA) (targeting Research Ethics Committees (RECs) specialised in reviewing early phase trials) - EUREC (European Network of Research Ethics Committees) - US Institutional Review Boards - Australia Health Research Ethics Committees registered through the National Human Medical Research Council - India Institutional Ethics Committees - Health Canada and Public Health Agency of Canada Research Ethics Board (PHAC REB) - South Korea Institutes Review Board - Executive Committee members professional contacts |
| Journal editors, associate editors and Conference Abstracts Review Committee Members | - Leading medical research journals that publish clinical trials, and targeted journals where many Phase I trials have been published (identified through methodological review) - International Committee of Medical Journal Editors (ICMJE) - Abstract review Committee members from leading conferences presenting Phase 1 results (see above) - Executive Committee members professional contacts |
| Funders / Funding Committee members | - Funding panels such as Medical Research Council (MCR), National Institute for Health and care Research (NIHR), Cancer Research UK (CRUK), Blood Cancer UK, Wellcome Trust, Melinda and Bill Gates Foundation, Great Ormond Street Hospital (GOSH) and other selected charities funding phase 1 work as applicable - USA National Institutes of Health (NIH) - Pharmaceutical companies - Executive Committee members professional contacts |
| Patients and Public | - Patient and Public engagement platforms - European Patients’ forum <https://www.eu-patient.eu/> - International disease specific advocacy groups - Patient representatives on Phase 1 trials management groups (through Clinical Trials Units portfolios) - Executive Committee members’ professional contacts |
