# Supplementary material for: Development of consensus-driven SPIRIT and CONSORT extensions for early phase dose-finding trials: the DEFINE study
Source: BMC Med. 2023 Jul 5;21:246. doi: 10.1186/s12916-023-02937-0 (PMC10324137; doi:10.1186/s12916-023-02937-0)
Supplement: Supplementary file 5 — Additional file 5. Further details on the statistical analysis plan. [file 12916_2023_2937_MOESM5_ESM.docx]

Further details on the statistical analysis plan (quantitative analyses)

As part of a sensitivity analysis, responses from Round One were carried over to Round Two if the respondents did not return for Round Two; it was assumed that they did not wish to change their responses. The scoring for each item was summarised as a continuous variable with a mean and standard deviation (SD), median (IQR), minimum, and maximum. The summary ratings were presented as: 1) R1, all Round One respondents, 2) R1*, all Round One respondents who returned to Round Two, 3) R2, Round Two respondents only, and 4) R2*, all Round Two respondents; the data from Round One were carried over for those who did not return.

In the analysis of the percentage of respondents scoring an item as “critically important” or “not important”, those with missing ratings were excluded from the analysis. Following visual inspection of the items scored by 1) stakeholder category, 2) years of early phase research, and 3) continents, if a group within each of these categories rated an item differently from another group or the overall, then those items were investigated further in subsequent rounds.

We tabulated the number of respondents changing their ratings for each item. The difference in numerical and categorical scores between Round Two and Round One for each item and respondent was computed. A positive change would indicate that the score at Round Two was higher than Round One.

Perfect agreement of each item between Rounds One and Two occurred when the individual participant provided the same numerical (categorical) score. The percentage of participants with perfect agreement was summarised, and the 95% confidence interval (CI) was estimated with the exact binomial method (R package “binom”) [21]. The other level of agreement between Rounds One and Two was also estimated with a weighted Cohen’s kappa coefficient using absolute error weights and its associated 95% CI estimated using bootstrapping (R package “irr”, function kappa2 with method="equal" and package boot with seed=5003) [20, 22-25].

All analyses were conducted in R (at least version 4.1.2), and the report was created using R Markdown.
