# Supplementary material for: Development of consensus-driven SPIRIT and CONSORT extensions for early phase dose-finding trials: the DEFINE study
Source: BMC Med. 2023 Jul 5;21:246. doi: 10.1186/s12916-023-02937-0 (PMC10324137; doi:10.1186/s12916-023-02937-0)
Supplement: Supplementary file 6 — Additional file 6. SPIRIT-DEFINE and CONSORT-DEFINE items generation for Delphi survey: Table A6-1. Table A6-1. References supporting the proposed candidate items in the DEFINE Delphi survey. [file 12916_2023_2937_MOESM6_ESM.docx]

# SPIRIT-DEFINE and CONSORT-DEFINE items generation for Delphi survey

Some papers were applicable to almost all the items, so for brevity they were not cited everywhere [7, 28-50]. Some articles were about ethics and risks [51-55], which are generally applicable to all trials, and no early phase dose-finding (EPDF) specific candidate items were proposed in the Delphi survey regarding ethics and risks. Chang *et al*. [56] presented “templates for phase I/II studies in newly diagnosed glioblastoma multiforme (GBM), recurrent malignant glioma, and recurrent meningioma,” which would also correspond to many SPIRIT-DEFINE items. Two highly relevant protocol templates [57, 58] were not referenced everywhere in the table below as they were applicable to most, if not all, items.

Some items from the original SPIRIT [8] and CONSORT [4] checklists did not need modification as they are relevant to EPDF trials, e.g., eligibility criteria for participants (item 10 in SPIRIT, item 4a in CONSORT); thus, they have not been included in the table below.

**Table A6-1: References supporting the proposed candidate items in the DEFINE Delphi survey.**

| **Checklist candidate items proposed in Delphi** | | **References** |
| --- | --- | --- |
| No. | **Title** | |
| [01/M] | SPIRIT: Identification as a first-in-human or early phase dose-finding trial |  |
| [02/M] | CONSORT: Identification as a first-in-human or early phase dose-finding trial |  |
|  | **Background and rationale** | |
| [03/M] | SPIRIT: Summary of findings from nonclinical/pre-clinical research | [34, 57, 59-68] |
| [04/M] | CONSORT: Summary of findings from nonclinical/pre-clinical research |  |
| [05/N] | SPIRIT: Description of findings from existing correlative biomarker, correlative and associated studies to support planned biomarker sub-study (if applicable) | [42, 57, 68] |
| [06/N] | CONSORT: Description of findings from existing correlative biomarker, correlative and associated studies to support planned biomarker sub-study (if applicable) |  |
|  | **Objectives** | |
| [07/M] | SPIRIT: Specific objectives (e.g., safety, activity, pharmacokinetics, pharmacodynamics) or hypotheses | [39, 49, 58, 61, 65, 69-76] |
| [08/M] | CONSORT: Specific objectives (e.g., safety, activity, pharmacokinetics, pharmacodynamics) or hypotheses |  |
|  | **Methods** | |
| [09/N] | SPIRIT: Trial design schema (to show flow of decision points, e.g., dose escalation to expansion) | [35, 56, 64, 77, 78] |
| [10/N] | CONSORT: Trial design schema (to show flow of decision points, e.g., dose escalation to expansion) |  |
| [11/N] | SPIRIT: Statistical methodology underpinning the trial design | [2, 33, 45, 60, 64, 69, 71, 72, 79-83] |
| [12/N] | CONSORT: Statistical methodology underpinning the trial design |  |
| [13/N] | SPIRIT: Starting dose(s) specification with rationale | [7, 35, 39, 42, 49, 59, 67, 74, 77, 83-90] |
| [14/N] | CONSORT: Starting dose(s) specification with rationale |  |
| [15/N] | SPIRIT: Dosing regimens (e.g., doses/schedules or intensity of fractionation) considered with rationale | [7, 41, 43, 45, 58, 59, 67, 71, 74, 76, 80, 83-86, 91-96] |
| [16/N] | CONSORT: Dosing regimens (e.g., doses/schedules or intensity of fractionation) considered with rationale |  |
| [17/N] | SPIRIT: Planned dosing regimens presented as a diagram or table | [45, 58, 83, 96] |
| [18/N] | CONSORT: Planned and delivered dosing regimens presented as a diagram or table |  |
| [19/N] | SPIRIT: Skipping of dose level | [83, 97] |
| [20/N] | CONSORT: Skipping of dose level |  |
| [21/N] | SPIRIT: Planned cohort size(s) (fixed or flexible) | [35, 39, 42, 49, 61, 87, 90, 94, 96] |
| [22/N] | CONSORT: Planned cohort size(s) (fixed or flexible) |  |
| [23/N] | SPIRIT: Sequence and interval between dosing of participants | [7, 35, 39, 42, 43, 49, 76, 77, 85, 90, 91, 94] |
| [24/N] | CONSORT: Sequence and interval between dosing of participants |  |
| [25/N] | SPIRIT: Pre-planned interim decision-making criteria/rules to guide the trial adaptation process (e.g., dosing decision to [de-]escalate); pre-planned timing and frequency of interim data looks and the information to inform the adaptations | [39, 43, 45, 49, 64, 80, 87, 88, 90, 98, 99] |
| [26/N] | CONSORT: Pre-planned interim decision-making criteria/rules to guide the trial adaptation process (e.g., dosing decision to [de-]escalate); pre-planned timing and frequency of interim data looks and the information to inform the adaptations |  |
| [27/N] | SPIRIT: Trial stopping criteria and consequences | [39, 43, 45, 49, 57, 58, 67, 71, 77, 78, 84-86, 90, 99, 100] |
| [28/M] | CONSORT: Trial stopping criteria and consequences |  |
| [29/N] | SPIRIT: Dose expansion cohort(s), if applicable | [57, 58, 64, 68, 75, 78, 80, 101] |
| [30/N] | CONSORT: Dose expansion cohort(s), if applicable |  |
| [31/M] | CONSORT: Important changes to the design or methods after trial commencement that are relevant for dose determination outside the scope of the pre-planned adaptive design features, with reasons | [2, 78, 81, 83, 102] |
|  | **Methods -> Interventions** | |
| [32/M] | SPIRIT: The interventions for each dose level (and within each treatment group for randomised early phase trials, if applicable) | [7, 35, 38, 58, 67, 100] |
| [33/M] | CONSORT: The interventions for each dose level (and within each treatment group for randomised early phase trials, if applicable) |  |
| [34/M] | SPIRIT: Criteria for discontinuing, dose modifications and dosing delays of allocated interventions for a given trial participant | [2, 31, 38, 57, 67, 81, 92, 98, 103, 104] |
| [35/M] | CONSORT: Criteria for discontinuing, dose modifications and dosing delays of allocated interventions for a given trial participant |  |
|  | **Methods -> Outcomes** | |
| [36/M] | SPIRIT: Define pre-specified primary and secondary outcome measures, including how and when they were assessed. Any other outcome measures used to inform pre-planned adaptations should be described with the rationale | [2, 39, 41, 62, 64, 65, 69, 70, 72, 73, 75, 77, 78, 80, 85, 98, 105-110] |
| [37/M] | CONSORT: Define pre-specified primary and secondary outcome measures, including how and when they were assessed. Any other outcome measures used to inform pre-planned adaptations should be described with the rationale |  |
|  | **Methods -> Sample size and operating characteristics** | |
| [38/M] | SPIRIT: Estimated number of participants (minimum/lower bound, maximum or expected range) needed to address study objectives and how it was determined, including clinical and statistical assumptions supporting any sample size AND operating characteristics | [2, 7, 33, 35, 39, 42, 44, 45, 83, 96, 97, 109, 111] |
| [39/M] | CONSORT: Estimated number of participants (minimum/lower bound, maximum or expected range) needed to address study objectives and how it was determined, including clinical and statistical assumptions supporting any sample size AND operating characteristics |  |
|  | **Methods -> Participant Timeline** | |
| [40/M] | SPIRIT: Time schedule of enrolment, interventions (including any run-ins and washouts), assessments, and visits for participants (including in-house stay or out-patient follow-up period where applicable). A schematic diagram is highly recommended | [7, 109, 110] |
|  | **Methods -> Recruitment** | |
| [41/N] | SPIRIT: Plans for recruitment/screening slots for sequential cohorts of participants | [39] |
|  | **Methods -> Assignment of interventions (for randomised trials) -> Sequence generation** | |
| [42/N] | SPIRIT: Any pre-planned allocation rule or algorithm to update randomisation with timing and frequency of updates |  |
| [43/N] | CONSORT: Any pre-planned allocation rule or algorithm to update randomisation with timing and frequency of updates |  |
| [44/N] | CONSORT: Any changes to the allocation rule after trial adaptation decisions |  |
|  | **Methods -> Data management** | |
| [45/M] | SPIRIT: Specify if the data management plans in the initial dose-finding component are different from subsequent stages (e.g., expansion cohort(s) or Phase II) of the trial |  |
|  | **Methods -> Statistical methods** | |
| [46/M] | SPIRIT: For the proposed adaptive design features, statistical methods used to estimate target dose(s), treatment effects for key endpoints and to make inferences | [2, 3, 33, 64, 69, 71, 80, 83, 95, 97, 109, 112-114] |
| [47/M] | CONSORT: For the implemented adaptive design features, statistical methods used to estimate target dose(s), treatment effects for key endpoints and to make inferences |  |
| [48/M] | SPIRIT: Statistical methods for additional analyses (e.g., subgroup and adjusted analyses, PK/PD, biomarker correlative analyses) | [7, 57, 82, 95, 106, 115] |
| [49/M] | CONSORT: Statistical methods for additional analyses (e.g., subgroup and adjusted analyses, PK/PD, biomarker correlative analyses) |  |
|  |  |  |
| [50/M] | SPIRIT: Clearly-defined analysis population (e.g., evaluable population for dose determination, safety and key outcomes) | [39, 71, 82, 87, 106] |
| [51/N] | CONSORT: Clearly-defined analysis population (e.g., evaluable population for dose determination, safety and key outcomes) |  |
| [52/N] | SPIRIT: Pre-specify handling strategies of events occurring after treatment initiation (e.g., how dosing delays will be handled) that affect either the interpretation or the existence of the measurements associated with the clinical question of interest | [39] |
| [53/N] | CONSORT: Pre-specify handling strategies of events occurring after treatment initiation (e.g., how dosing delays will be handled) that affect either the interpretation or the existence of the measurements associated with the clinical question of interest |  |
| [54/N] | SPIRIT: Statistical software and packages used for design (e.g., simulation) and to be used for planned analyses | [39, 96] |
| [55/N] | CONSORT: Statistical software and packages used |  |
|  | **Methods -> Monitoring** | |
| [56/M] | SPIRIT: Any decision-making group or safety review committee, alternatively, an explanation of why such a committee is not needed | [32, 35, 37, 39, 45, 49, 84, 86, 90, 99, 116-119] |
| [57/M] | CONSORT: Any decision-making group or safety review committee |  |
| [58/M] | SPIRIT: Description of the plans for any interim data review (including data to be used for decision-making) and interim statistical analyses (e.g., safety/toxicity, dose [de-]escalation decisions) and stopping guidelines | [32, 35, 37, 39, 42, 45, 49, 84, 86, 90, 95, 99, 100, 119] |
| [59/M] | CONSORT: Description of the plans for any interim data review (including data to be used for decision-making) and interim statistical analyses (e.g., safety/toxicity, dose [de-]escalation decisions) and stopping guidelines |  |
|  | **Results -> Participant flow** | |
| [60/M] | CONSORT: For each group, the number of participants who were assigned to each dose level at each interim analysis (e.g., for dosing decisions), received intended treatment, and were analysed for the primary outcome and, if applicable, any other outcomes used to inform pre-planned adaptations | [78, 82] |
|  | **Results -> adaptations** | |
| [61/N] | CONSORT: Trial adaptation decisions made in light of the pre-planned decision-making criteria and observed accrued data | [2, 39, 81, 86, 98] |
|  | **Results -> Baseline data** | |
| [62/M] | CONSORT: Baseline demographic and clinical characteristics across each dose level within each group | [82] |
|  | **Results -> Numbers analysed** | |
| [63/M] | CONSORT: For each group, the number of participants (denominator) included in each (interim/final) analysis across each dose level, and whether the analysis was by original assigned interventions | [82] |
|  | **Results -> Outcomes and estimation** | |
| [64/M] | CONSORT: For each primary and secondary outcome, results for each dose level within each group, and the estimated effect size and its precision if applicable | [2, 72, 82] |
| [65/N] | CONSORT: Report interim results used to inform interim decision-making such as dose escalation, de-escalation or staying at the same dose | [45, 49, 90, 99] |
|  | **Results -> Harms** | |
| [66/M] | SPIRIT: Plans for collecting, assessing, reporting, and managing solicited and spontaneously reported adverse events and other unintended effects of trial interventions (e.g., prior to any planned next dosing) or trial conduct | [35, 39, 42] |
| [67/M] | CONSORT: All important toxicities and adverse events reported by dose level in each group (for specific guidance see CONSORT for harms) | [109, 119, 120] |
|  | **Ethics and dissemination** | |
| [68/M] | SPIRIT: Specify if and when results (e.g., DLT, response outcomes) can be shared whilst the trial is still ongoing | [68, 69, 120] |
| [69/M] | CONSORT: Specify if and when results (e.g., DLT, response outcomes) were reported whilst the trial was still ongoing |  |
|  | **Other information** | |
| [70/M] | CONSORT: Where the full trial protocol or the redacted version, with amendments (if any), can be accessed | [37, 68] |
| [71/N] | SPIRIT: Where other relevant trial documents (Oversight Committee, Safety Review Charter, quality aspects of investigational medicinal product, investigators brochure, simulation report, this list is non-exhaustive) can be accessed |  |
| [72/N] | CONSORT: Where the full statistical analysis plan and other relevant trial documents (Oversight Committee, Safety Review/Data Monitoring Committee Charter, quality aspects of investigational medicinal product, investigators brochure, simulation report, this list is non-exhaustive) can be accessed | [118, 120] |
|  | **Appendices -> Dose Transition Pathways** | |
| [73/N] | SPIRIT: Dose transition pathways or dose decision paths (using, for example, flow diagram or table) projecting in advance how a proposed dose-finding design will recommend doses | [39, 45, 49, 90, 121] |
|  | **Patient (service users) / their carer or members of public involvement (PPI)** | |
| [74/N] | SPIRIT: PPI involvement | [36, 118, 122] |
| [75/N] | CONSORT: PPI involvement |  |
|  | **Lay summary** | |
| [76/N] | SPIRIT: Lay summary or where it can be accessed | [123, 124] |
| [77/N] | CONSORT: Lay summary or where it can be accessed |  |
|  | **Abstract (of conferences and journal articles)** |  |
| [78/M] | CONSORT-abstract: Identification as a dose-finding trial |  |
| [79/N] | CONSORT-abstract: Dose decisions/adaptations were made in light of pre-planned decision-making criteria and observed accrued data | [45, 49, 80, 90, 99] |

[../M] – Items that are modified from the original SPIRIT and CONSORT guidance;

[../N] – New items introduced for the DEFINE study.
