# Supplementary material for: Development of consensus-driven SPIRIT and CONSORT extensions for early phase dose-finding trials: the DEFINE study
Source: BMC Med. 2023 Jul 5;21:246. doi: 10.1186/s12916-023-02937-0 (PMC10324137; doi:10.1186/s12916-023-02937-0)
Supplement: Supplementary file 7 — Additional file 7. Toolkit for Lay Summary of EPDF Trial Results. [file 12916_2023_2937_MOESM7_ESM.pdf]

Additional file 7

Toolkit for lay summary of early phase dose-finding clinical trial results

The overall aim of the Patient and public involvement and engagement (PPIE) in this study is to embed the perspective of patients as key stakeholders in the DEFINE development process for both SPIRIT and CONSORT extension for early phase dose-finding (EPDF) trials, as well as to facilitate the dissemination of the resulting guidelines.

To do this, a PPIE representative (AK) was invited to be part of the DEFINE executive committee from the onset to help shape the draft candidate items, including participating in the methodological review of the reporting quality of early phase dose-finding (EPDF) published trials [1] The resulting draft of candidate items was sent to 3 experienced PPIEs, ahead of the Delphi survey to assess if patients and participants could fully contribute. The main feedback from this review was that the technicality of the items might be a barrier, as significant background knowledge of EPDF as well as of the context of the CONSORT and SPIRIT guidelines would be required, with one reviewer stating: “This will be hard for anyone not familiar with SPIRIT or CONSORT, or at least the concepts behind them if not the full contents.” As a result, a formal PPIE working group was established to see how we can best embed patients’ perspective in the DEFINE development process. PPIE representatives with experience in early phase clinical trials were still invited to contribute to the Delphi survey (4 participated) and two participated in the subsequent DEFINE consensus meeting.

The PPIE working group explored patients and participant’s perspectives regarding the reporting of EPDF trials. The discussion highlighted the need to develop a lay summary toolkit to provide easy-to-understand summaries of scientific publications of EPDF trials to provide increased transparency to the patients and the public, with an associated published EPDF trial example to facilitate implementation. The development process included a review of publicly available examples of lay study results summaries [126-131], as well as lay summary guidance [132-138] from a variety of disease settings as well as academia and the pharmaceutical industries. Through expert discussions the most relevant items were selected and an appropriate structure, format as well as detailed guidance notes devised. The draft template was then piloted with a published dose-finding trial to ensure it was fit for purpose and produce a best practice exemplar. The drafted template, guidance and exemplar was then sent for review to 3 professional experts (a Communications Media Manager, an Ethics Committee member, and a Regulator) to gather feedback and further refinement, before a wider consultation with the Patient and public Involvement and engagement in Musculoskeletal reSearch (PIMS) group.

The resulting lay result summary toolkit was very well received by the experts and additional PPIE group, and further plans include dissemination of the toolkit to improve reporting of EPDF trial results to lay audiences. One limitation is that the toolkit was not independently tested, however we are confident the thorough engagement with relevant stakeholder groups and development processes to elicit important information and how best to present them will mitigate this. Additionally, the varied background of the PPIE representatives including patients and carers from non-oncology and non-oncology settings, with different degree of expertise and familiarity with EPDF trials, and multistakeholder experts involved in the working group and subsequent consultations should ensure the tool is applicable to a multitude of studies and settings. The dissemination will also be used as an opportunity to gain wider feedback on the usability of the toolkit and refine it further if necessary.

Acknowledgements

We would like to thank Elizabeth Abbott, Mark Chegwidden, Annabel Dawson, Rosie Flower, Liz Hammer, Sue Leeder, Cate Middleton, Mike Solomon and Pamela Spearing from the Patient and public Involvement and engagement in Musculoskeletal reSearch (PIMS) group, as well as Arthur Pratt, Leigh Romaniuk, Julia Bakker, Karen Poole and Kate Craig for their valuable input in the development of the lay summary toolkit for reporting of early phase trial results.

Lay summary template for EPDF trial results

Title/Headline

- 1-2 sentences summarising the key findings/main outcomes of the trial.
- State the dates the period the trial ran for, and the date the summary was written

Why was this research needed?

- What was the problem/what was the question? – Brief explanation of the background and hypothesis/main objective(s).

What were the conclusions?

- High level overview of the study results

Who volunteered to take part in the study?

- Brief description of the study population

How did we deliver the trial?

- Brief description of the research team, PPIE involvement, recruitment dates, where it took place and any other relevant information.

How was the trial done?

- Description of the intervention

**Name of drug or intervention:**	**Route (e.g):**	**Dose/Dose level or ranges:**	**Frequency (e.g.):**	**Cycle (e.g.):**
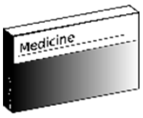	Injection<br>Tablets/ caplets taken by mouth...	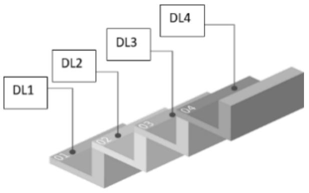	Twice a day; weekly;	28 days;4 days on, 3 days off.

- Brief description of the mechanism of action if appropriate
- How was the safety of the participants monitored?

What were the results?

What worked well:

What remains unclear

What didn't work well

What were the main challenges for the patients with the treatment?

- Explain treatment side effects as well as any quality of life/patient reported outcome measures used in the trial.

What happens next?

- Discuss the results of the study
- Explain any plans for further studies, already running or planned.

Where can I find more information?

- Full study name, funder and sponsor
- Link to full results/scientific summary as appropriate

Acknowledgments

- We would like to thank all the participants that volunteered to take part in this trial as well as the site and trial teams that helped deliver this trial.

Lay summary template for EPDF trial results - Guidance Notes

General guidance:

- Consider who will write the lay results summary. Could the trial lay representative be producing this summary?
- Consider where and how this will be disseminated (to relevant charities/advocacy groups/patients organisations etc) as well as linking to main results, and how this might impact the summary content as well as layout/graphic design etc of the document (e.g., use of QR codes instead of links on printed documents)
- Think about your audience. What details will be relevant to them? Remove jargon and make it accessible to as wide a public as possible (readers may not only be patients with a vested interest.). Consider reading age. Spell out the findings clearly and in language the audience understands and do not use words like 'outcomes' without explaining what they are.. Did the treatment shrink tumours, help prevent cancer getting worse or help patients live longer, for example?
- The summary should tell a story – think about developing a narrative using the sections below, putting the "punchline" upfront.
- The language should be kept neutral and non-promotional. Consider ethical implications if benefit/efficacy or "promising" results are stated.
- Keep the language consistent throughout, for example when using the terms study/trial – keep to the same term.
- Tables/ statistical information should be avoided. Simple graphics and statements should be used instead.
- Keep it brief – preferably up to 2-3 pages

Further advice and resources are available at: https://www.nihr.ac.uk/documents/plain-english-summaries/27363

Evidence-Based graphics resources are available at: http://www.vizhealth.org/

Title/Headline

- 1-2 sentences summarising the key findings/main outcome(s) of the trial.
- The section should mention the results are from an early phase trial.
- Period the study ran for, and date the summary was written should be included.

Further advice and guidance on writing title are available at: https://plos.org/resource/how-to-write-a-great-title/

What were the conclusion?

- This section could be seen as a "lay abstract", providing high level overview of the study results (beyond the main headline described above)
- Avoid jargon and be clear about what was observed. Use phrases like shrunk tumours cleared infection or helped patients live longer.
- Use of non-promotional language.
- State the results do not translate into clinical benefit, how the study has helped (who/what?) and briefly outline the implications of these results for the current research landscape (e.g., further trials planned or not)
- Consider your audience, and what you want them/what they can do with this information.

Why was this research needed?

- Identify the condition or diagnosis. Provide brief explanation of the rationale for the trial, identifying the state of knowledge before the trial and the key question it was trying to answer.
- Include one or two sentences that provide key background information.

Who volunteered to take part in the trial?

- State the number of participants involved.
- State the type and stage of disease. E.g., for cancer, state the type of cancer and the stage of cancer.
- Consider the relevance and amount of information provided on trial population. What is important/relevant to provide for this specific question/trial?

How did we deliver the trial?

- State the type and stage of disease. E.g., for cancer, state the type of cancer and the stage of cancer.
- State who the research team are and highlight PPIE involvement in the design or the study delivery.
- State where the study took place, and the period of time patients were recruited over (if different from the dates indicated in the headline).

How was the trial done?

- Describe the intervention in simple pictorial manner (if possible/appropriate) Consider how best to depict the intervention at an individual (patient) level, as well as any group/cohorts (as appropriate).
- Focus on what actually happened in the trial, and not what was originally planned (though if important or relevant changes were made during the course of the trial, this should be highlighted and discussed in next sections.
- Consider including information on the mechanism of action of the treatment.
- Provide information on procedures and frequency of follow-up and safety monitoring (on and off treatment)
- Ensure that patient reported outcomes are given equal importance to outcomes related to drug effect.

What were the results?

- To avoid repetition, summarise this with key statistics from the trial. E.g., X % of participants could tolerate this dose.
- This section should be used to report on the trial conduct and results and reflect/assess success/performance against what the trial set out to achieve (consider trial conduct as well as scientific objectives):
  - Trial recruited to target, what safe doses were identified, hypotheses proved. This section should highlight positives from the trial conduct and results.
  - Any issues that necessitated changes to the trial.
  - Any questions that weren't answered or that emerged due to the results

Note that all three green, amber and red sections may not be needed or appropriate for all trials.

What were the main challenges for patients with the treatment?

- Side effects observed. Adverse events/reactions jargon should be avoided. Side effects should ideally not be listed in a table but summarised by relevance and put into context (expectedness, overall tolerability, comparison with other treatments… as appropriate).
- Include PROMS and QoL results as appropriate to contextualise tolerability.

What happens next?

- Any plans/recommendations to answer the remaining questions stated in the section above.
- Explain any plans for further studies, already running or planned.

Where can I find more information?

- Full study name, funder and sponsor
- When including link to scientific publication, consider appropriate format (depending on how the summary is disseminated) as well as how accessible the linked publication is (journal subscriptions, etc). Also consider the use of QR codes or any other support to increase accessibility.
- If appropriate, include a contact to allow readers to get in touch.

Acknowledgments/Statement of thanks:

- Use this statement to thanks the study participants and acknowledge their contribution to the study, as well as sites and trial teams and any other relevant contributor.

A lay summary example based on a published EPDF trial

First clinical trial to test a new drug combination treatment that boosts the immune system's ability to fight some cancers.

This trial was conducted between January 2017 to January 2020. This summary was written in February 2023.

Why was this research needed?

- **Immunotherapy** is a type of treatment that can help the body fight cancer by boosting the immune system.
- One effective immunotherapy drug for treating solid tumours (tumours that are located in the organs) is called **pembrolizumab**. Unfortunately, sometimes these tumours can become resistant to immunotherapies, and the tumours can grow with the disease spreading. This is called “progression.”
- Previous research suggests that a drug called **guadecitabine** may help increase the effectiveness of immunotherapy on these resistant tumours. To investigate this, we conducted a type of research study called a clinical trial. The aim was to find out if combining guadecitabine and pembrolizumab is safe for patients with resistant solid tumours, without causing bad side effects.

What were the conclusions?

- This trial found the best dose level, timing, and safety for giving patients the helper drug guadecitabine together with pembrolizumab.
- The trial showed that over a period of 24 weeks, the cancer remained stable in more than three out of every ten patients who received the new drug combination.

Who volunteered to take part in the study?

- The trial recruited 34 patients with advanced solid tumours, with 14 in the first stage and 20 in the second. The participants were aged between 47 and 73.5 years old. There were 17 male and 17 female patients. All the patients had cancer that had progressed too far for the standard treatments to work.

How did we deliver the trial?

- This trial was led by researchers at the Royal Marsden Hospital. It took place at the Royal Marsden Hospital and University College London Hospital in the UK. We designed the study to have two stages:
    - Stage 1 was aimed at finding the recommended dose of guadecitabine to be given with pembrolizumab.

- Stage 2 involved recruiting more patients to further explore the effects of the drug combination.

How was the trial done?

Drugs:	Administration:	Doses:	Frequency:	Cycle:
Guadecitabine	Injection under the skin (subcutaneously)	Low dose 30mg OR High dose 45mg	Daily on days 1-4 of each cycle	3 week cycle
Pembrolizumab	Into the vein (intravenously)	200mg	On first day of each cycle, starting on cycle 2	

- In the first stage of the trial, 14 patients were given 200mg of pembrolizumab along with either a low or high dose of the helper drug guadecitabine.
- In the second stage, 20 patients were given the recommended dose of guadecitabine (found to be 30mg during the first) along with 200mg pembrolizumab.
- Throughout the trial, patient safety was assessed every three weeks through physical examination, heart monitoring, and analysis of urine and blood samples.

What were the results?

- We found that combining a low dose of the helper drug guadecitabine with a standard dose of pembrolizumab is safe for patients.
- This combination also made tumours sensitive to immunotherapy again, and for 37% of patients who received the drug combination, their cancers did not progress for at least24 weeks.

- Researchers analysed tumour samples from the patients who had benefited from the treatment. This laboratory work found a marker that in future could be used to identify other patients who might be helped by the treatment.

- A higher dose of guadecitabine caused some severe side effects. This meant that some patients could not continue with the treatment at this high dose.

What were the main challenges for the patients with the treatment?

- Patients who received the recommended dose of guadecitabine (30mg) along with pembrolizumab experienced side effects that are related to the treatment. These include a low neutrophil count (a type of white blood cell), fatigue, and nausea.
- Patients experienced some other side effects, but these were mostly mild.

What happens next?

- This trial is the first of its kind to investigate the combination of guadecitabine and pembrolizumab for treating solid tumours. However, as only 34 patients took part, it is too early to say if the treatment can be put into widespread use.
- As some patients in the trial had not previously received immunotherapy, more research is needed to determine if this treatment would be effective in this group of patients.

- The next steps will be to conduct additional trials to confirm the promising results and to identify groups of patients who may benefit from this treatment based on the genetic makeup of their tumours

Where can I find more information?

- Full title of the trial: Phase 1, dose-escalation study of guadecitabine (SGI-110) in combination with pembrolizumab in patients with solid tumours.
- This trial was sponsored by Royal Marsden NHS Foundation Trust and funded by Astex Pharmaceuticals Inc. (manufacturer of Guadecitabine) and Merck, Sharp and Done LLC (manufacturer of Pembrolizumab).
- Full scientific publication available at:

https://jitc.bmj.com/content/jitc/10/6/e004495.full.pdf

- For more information, please contact <<<insert contact>>>

Acknowledgments

We would like to thank all the patients who volunteered to take part in this trial as well as the site and trials teams who helped deliver it.
