# Supplementary material for: Development of consensus-driven SPIRIT and CONSORT extensions for early phase dose-finding trials: the DEFINE study
Source: BMC Med. 2023 Jul 5;21:246. doi: 10.1186/s12916-023-02937-0 (PMC10324137; doi:10.1186/s12916-023-02937-0)
Supplement: Supplementary file 8 — Additional file 8. Delphi survey participants demographics and harmonisation of roles: Tables A8-1 – A8-2. Table A8-1. Demographics of Delphi survey participants. Table A8-2. Harmonisation of participants’ defined roles. [file 12916_2023_2937_MOESM8_ESM.docx]

# Delphi survey participants demographics and harmonisation of roles

## **Table A8-1. Demographics of Delphi survey participants**

| **Descriptions** | **Registered** | **Round One** | **Round Two** |
| --- | --- | --- | --- |
|  | **(N=244)** | **(N=206)** | **(N=151)** |
|  | n(%) | n(%) | n(%) |
| ***Early phase roles**** |  |  |  |
| Clinicians/clinical pharmacologists | 79(32.4%) | 63(30.6%) | 44(29.1%) |
| Ethics committee/institutional review board members | 21(8.6%) | 19(9.2%) | 15(9.9%) |
| Funders/funding committee members | 13(5.3%) | 12(5.8%) | 10(6.6%) |
| Journal editors, associate editors and conference abstracts committee members | 23(9.4%) | 18(8.7%) | 15(9.9%) |
| Regulators | 19(7.8%) | 15(7.3%) | 13(8.6%) |
| Statisticians/trial methodologists/data scientists/quantitative analysts | 115(47.1%) | 100(48.5%) | 73(48.3%) |
| Trial management staff (including trial managers/co-ordinators and data managers | 29(11.9%) | 26(12.6%) | 18(11.9%) |
| Others | 14(5.7%) | 10(4.9%) | 7(4.6%) |
| ***Other role(s)*** |  |  |  |
| Consultant | 2(0.8%) | 2(1%) | 1(0.7%) |
| Medical writer | 2(0.8%) | 2(1%) | 2(1.3%) |
| Patients, public, patient advocates | 5(2.1%) | 4(1.9%) | 4(2.7%) |
| Postgraduate research student | 1(0.4%) | 1(0.5%) | 0 |
| Trial investigators (including scientist, researcher, etc.) | 4(1.6%) | 1(0.5%) | 0 |
| ***Types of main employment organisation*** |  |  |  |
| Commercial organisation (e.g., pharmaceutical, biotech, contract research organisation) | 45(18.4%) | 38(18.5%) | 18(11.9%) |
| Independent | 6(2.5%) | 6(2.9%) | 4(2.7%) |
| Non-commercial organisation (e.g., academia, hospital, charity, government organisation, non-profit organisation) | 189(77.5%) | 158(76.7%) | 125(82.8%) |
| Other types of main employment organisation | 4(1.6%) | 4(1.9%) | 4(2.7%) |
| ***Other types of main employment organisation***** |  |  |  |
| I'm a consultant | 1(0.4%) | 1(0.5%) | 1(0.7%) |
| Medical journal | 1(0.4%) | 1(0.5%) | 1(0.7%) |
| Patient Representative | 1(0.4%) | 1(0.5%) | 1(0.7%) |
| Retired | 2(0.8%) | 2(1%) | 2(1.3%) |
| ***Area of clinical research**** |  |  |  |
| Cancer trials | 187(76.6%) | 157(76.2%) | 112(74.2%) |
| Non-cancer trials | 142(58.2%) | 122(59.2%) | 87(57.6%) |
| Trials involving healthy volunteers | 94(38.5%) | 80(38.8%) | 56(37.1%) |
| None of the above | 12(4.9%) | 11(5.3%) | 10(6.6%) |
| ***Years of clinical research*** |  |  |  |
| < 1 year | 6(2.5%) | 5(2.4%) | 5(3.3%) |
| 1-5 years | 30(12.3%) | 27(13.1%) | 20(13.3%) |
| 6-14 years | 81(33.2%) | 67(32.5%) | 45(29.8%) |
| 15 years or more | 127(52.1%) | 107(51.9%) | 81(53.6%) |
| ***Years of early phase research*** |  |  |  |
| < 1 year | 29(11.9%) | 26(12.6%) | 23(15.2%) |
| 1-5 years | 62(25.4%) | 54(26.2%) | 38(25.2%) |
| 6-14 years | 76(31.2%) | 62(30%) | 46(30.5%) |
| 15 years or more | 77(31.6%) | 64(31.1%) | 44(29.1%) |
| ***Number of roles*** |  |  |  |
| 1 | 193(79.1%) | 165(80.1%) | 121(80.1%) |
| 2 or more | 51(20.9%) | 41(20%) | 30(19.8%) |
| ***Continent of residence*** |  |  |  |
| Africa | 3(1.2%) | 3(1.5%) | 1(0.7%) |
| Asia | 20(8.2%) | 17(8.3%) | 14(9.3%) |
| Australia/Oceania | 6(2.5%) | 5(2.4%) | 3(2%) |
| Europe | 146(59.8%) | 128(62.1%) | 102(67.6%) |
| North America | 69(28.3%) | 53(25.7%) | 31(20.5%) |
| ***Country of residence*** |  |  |  |
| Australia | 6(2.5%) | 5(2.4%) | 3(2%) |
| Austria | 1(0.4%) | 1(0.5%) | 1(0.7%) |
| Belgium | 2(0.8%) | 2(1%) | 1(0.7%) |
| Canada | 16(6.6%) | 15(7.3%) | 9(6%) |
| China | 9(3.7%) | 8(3.9%) | 5(3.3%) |
| Czechia | 1(0.4%) | 1(0.5%) | 0 |
| Denmark | 1(0.4%) | 1(0.5%) | 1(0.7%) |
| France | 14(5.7%) | 12(5.8%) | 11(7.3%) |
| Germany | 6(2.5%) | 5(2.4%) | 4(2.7%) |
| India | 2(0.8%) | 1(0.5%) | 2(1.3%) |
| Italy | 2(0.8%) | 2(1%) | 1(0.7%) |
| Japan | 2(0.8%) | 2(1%) | 2(1.3%) |
| Kenya | 1(0.4%) | 1(0.5%) | 1(0.7%) |
| Malaysia | 1(0.4%) | 1(0.5%) | 1(0.7%) |
| Netherlands | 3(1.2%) | 3(1.5%) | 2(1.3%) |
| Norway | 1(0.4%) | 1(0.5%) | 1(0.7%) |
| Russia | 1(0.4%) | 0 | 0 |
| Singapore | 4(1.6%) | 3(1.5%) | 3(2%) |
| South Africa | 1(0.4%) | 1(0.5%) | 0 |
| South Korea | 2(0.8%) | 2(1%) | 1(0.7%) |
| Spain | 4(1.6%) | 1(0.5%) | 0 |
| Switzerland | 7(2.9%) | 7(3.4%) | 5(3.3%) |
| Uganda | 1(0.4%) | 1(0.5%) | 0 |
| United Kingdom (UK) | 103(42.2%) | 92(44.7%) | 75(49.7%) |
| United States of America (USA) | 53(21.7%) | 38(18.5%) | 22(14.6%) |
| * Multiple entries per participant were allowed. ** Note that although there were four participants who specified that their main employment was ‘other’, five provided further details on their ‘other’ employment. One of them identified as Retired in a non-commercial organisation. | | | |

## **Table A8-2. Harmonisation of participants’ defined roles**

| **Predefined roles** | **Participants defined “Other” roles** |
| --- | --- |
| Clinicians/clinical pharmacologists | “clinical trial pharmacist”  “clinician-scientist”  “medical officer”  “nurse specialist histopathology and oncology research”  “oncologist pharmacist dealing with phase 1 trial drugs” and “pharmacist” |
| Statisticians/trial methodologists/data scientists/quantitative analysts | “outcomes methodologist” |
| Trial management staff (including trial managers/co-ordinators and data managers | “programmer”  “quality assurance”  “research nurse manager” |
|  |  |
| **New harmonised roles** |  |
| Patients, public, patient advocates | “cancer research and patient advocate”  “patient advocate”  “patient engagement practitioner in the clinical trials/research space”  “PPI” |
| Trial investigators (including scientist, researcher, etc.) | “clinical development”  “neuroscientist/clinical researcher/professor”  “preclinical scientist”  “scientist” |
