# Supplementary material for: Development of consensus-driven SPIRIT and CONSORT extensions for early phase dose-finding trials: the DEFINE study
Source: BMC Med. 2023 Jul 5;21:246. doi: 10.1186/s12916-023-02937-0 (PMC10324137; doi:10.1186/s12916-023-02937-0)
Supplement: Supplementary file 9 — Additional file 9. Perception of proposed items and sensitivity analysis: Tables A9-1 – A9-3, Figures A9-1 – A9-13. Table A9-1. Summary of categorical and numerical ratings at Round One and 2. Figure A9-1. Percentage of participants changing their numerical scores at Round Two. Figure A9-2. Percentage of participants changing their categorical scores at Round Two. Table A9-2. Number of participants changing their ratings between Round One and Round Two. Table A9-3. Frequency and percentage of perfect agreement. Figure A9-3. Stacked bar plots of candidate item [17 SPIRIT-DEFINE] “Planned dosing regimens presented as a diagram or table, where applicable” by stakeholders. Figure A9-4. Stacked bar plots of candidate item [18 CONSORT-DEFINE] “Planned and delivered dosing regimens presented as a diagram or table, where applicable” by stakeholders. Figure A9-5. Stacked bar plots of candidate item [48 SPIRIT-DEFINE] “Statistical methods for additional analyses” by stakeholders. Figure A9-6. Stacked bar plots of candidate item [69 CONSORT-DEFINE] “Specify if and when results were reported whilst the trial was still ongoing” by stakeholders. Figure A9-7. Stacked bar plots of candidate item [70 SPIRIT-DEFINE] “Where the full trial protocol or the redacted version, with amendments, can be accessed” by stakeholders. Figure A9-8. Stacked bar plots of candidate item [17 SPIRIT-DEFINE] “Planned dosing regimens presented as a diagram or table, where applicable” by Continent. Figure A9-9. Stacked bar plots of candidate item [18 CONSORT-DEFINE] “Planned and delivered dosing regimens presented as a diagram or table, where applicable” by Continent. Figure A9-10. Stacked bar plots of candidate item [48 SPIRIT-DEFINE] “Statistical methods for additional analyses” by Continent. Figure A9-11. Stacked bar plots of candidate item [69 CONSORT-DEFINE] “Specify if and when results were reported whilst the trial was still ongoing” by Continent. Figure A9-12. Stacked bar plots of candidate item [7 [file 12916_2023_2937_MOESM9_ESM.docx]

# Perceptions of proposed candidate items and sensitivity analysis

## Perceptions of proposed candidate items

Figure 3 of the main paper presents the percentage of respondents in Round Two (N = 151) scoring each candidate item. Of 80 candidate items, 60 had at least 70% of respondents rating them as “critically important” (scores 7 to 9) (See Table A9-1). All 52 items in Round One that met the inclusion threshold remained highly scored in Round Two.

Every item had more than 15% of respondents changing their numerical ratings (see Figure A9-1), but only eight items had more than 15% of respondents changing their categorical ratings (see Figure A9-2). This suggests that although there were many respondents changing their numerical ratings for each item, those changes were not substantial enough to cause a change in categorical level. Almost all items had at least one participant changing their categorical rating, with candidate item 6 [(New) CONSORT: *Summary of findings from existing correlative biomarker, correlative and associated studies to support planned biomarker sub-study (if applicable)*] having the largest number of 14 respondents changing their scores (n = 4 from “not important” to “important, but not critical”; n = 1 from “important, but not critical” to “critical”; and n = 9 from “critical” to “important, but not critical”) (see Table A9-2).

In our assessment of the stability and consistency of individual ratings of item importance across rounds, we found a reasonably high level of individual agreement. Twenty items had moderate agreement (0.41 to 0.6) and 59 have substantial agreement (0.61 to 0.8) (see Table A9-3). This suggested that respondents had “converged” in their responses. Following discussion among members of the Executive Committee and Independent Expert Panel, it was agreed that there was no utility in running another round of the Delphi survey.

Visual inspection of the item rating by early phase roles showed that there was no difference for most items except for candidate items [17 SPIRIT-DEFINE/18 CONSORT-DEFINE] *“Planned dosing regimens presented as a diagram or table, where applicable”*, [48 SPIRIT-DEFINE] *“Statistical methods for additional analyses (e.g., subgroup and adjusted analyses, PK/PD, biomarker correlative analyses)”*, [69 CONSORT-DEFINE] *“Specify if and when results (e.g., safety/response outcomes) were reported whilst the trial was still ongoing”* and [70 CONSORT-DEFINE] *“Where the full trial protocol or the redacted version, with amendments (if any), can be accessed”* (see Figures A9-3 to A9-7). A greater percentage of respondents identified as funders (8/10; 80%) voted items #17 and #18 as ‘critically important’. Statisticians (52/73; 71%), regulators (11/13; 85%) and others (6/7; 86%) voted item #48 as ‘critically important’ compared to the other stakeholders. Whereas a greater percentage of clinicians (33/43; 75%), regulators (11/13; 85%) and funding panel members (8/10; 80%) voted item #69 as ‘critically important’ and only statisticians (53/73; 73%) rated item #70 as ‘critically important’. All these items were also rated by more respondents from Asia than from Europe and North America (see Figures A9-8 to A9-12). A higher proportion of respondents from Asia (11/14; 78.6%) voted candidate item [5 SPIRIT-DEFINE] *“Summary of findings from existing correlative biomarker, correlative and associated studies to support planned biomarker sub-study (if applicable)*” as ‘critically important’ (Figure A9-13).

A visual inspection of the ratings of respondents revealed broad consistency across years of early phase trial experience in both rounds one and two.

## Sensitivity analysis

Data from Round One were carried forward for those who did not return to Round Two. From the 210 respondents, only 57 items would have been automatically included in the checklists, compared to the 60 based on the data from Round Two respondents only. The three items that did not meet the threshold to be included were [item 4 CONSORT-DEFINE] ‘*Summary of* *key findings from relevant nonclinical/pre-clinical research’* (R2 only, 70.9% vs. R2 with R1 carry forward, 65.7% respondents rating it as ‘critically important’), [item 49 CONSORT-DEFINE] ‘*Statistical methods for additional analyses (e.g., subgroup and adjusted analyses, PK/PD, biomarker correlative analyses)’* (70.7% vs. 64.5%) and [item 68 SPIRIT-DEFINE] ‘*Specify if and when results (e.g., safety/response outcomes) can be shared externally/were reported whilst the trial is still ongoing’* (73.2% vs. 68.5%) (see Table A9-1). These items marginally met the inclusion threshold based on Round Two respondents and would be excluded from the sensitivity analysis.

*Table A9-1. Summary of categorical and numerical ratings at Round One (all R1 respondents), at Round One* (R1 respondents who also responded at R2), at Round Two (all R2 respondents) and at Round Two* with data carried over from Round One for those who did not return to Round Two. (N: new candidate item; M: modified candidate item, adapted from SPIRIT 2013 or CONSORT 2010)*

|  |  | Missing | Not important | Important, but not critical | Critically important | Unable to rate | N | Mean (sd) | Median (IQR); (min, max) |
| --- | --- | --- | --- | --- | --- | --- | --- | --- | --- |
| [1.2/M] Identification as a first-in-human or early phase dose-finding trial | | | | | | | | | |
| SPIRIT | 1 | 0 | 2 (0.97) | 28 (13.59) | 172 (83.5) | 4 (1.94) | 202 | 7.9 (1.37) | 8 (7 to 9); (3, 9) |
|  | 1* | 0 | 1 (0.68) | 16 (10.96) | 127 (86.99) | 2 (1.37) | 144 | 8.03 (1.23) | 8 (7 to 9); (3, 9) |
|  | 2 | 0 | 0 (0) | 8 (5.3) | 143 (94.7) | 0 (0) | 151 | 8.48 (0.91) | 9 (8 to 9); (5, 9) |
|  | 2* | 0 | 1 (0.48) | 20 (9.52) | 187 (89.05) | 2 (0.95) | 208 | 8.23 (1.22) | 9 (8 to 9); (3, 9) |
| CONSORT | 1 | 0 | 2 (0.97) | 31 (15.05) | 169 (82.04) | 4 (1.94) | 202 | 7.85 (1.36) | 8 (7 to 9); (3, 9) |
|  | 1* | 0 | 1 (0.68) | 17 (11.64) | 126 (86.3) | 2 (1.37) | 144 | 8 (1.22) | 8 (7 to 9); (3, 9) |
|  | 2 | 0 | 0 (0) | 8 (5.3) | 143 (94.7) | 0 (0) | 151 | 8.49 (0.89) | 9 (8 to 9); (5, 9) |
|  | 2* | 0 | 1 (0.48) | 22 (10.48) | 185 (88.1) | 2 (0.95) | 208 | 8.21 (1.23) | 9 (8 to 9); (3, 9) |
| [3.4/M*] Summary of key findings from relevant nonclinical/pre-clinical research | | | | | | | | | |
| SPIRIT | 1 | 0 | 3 (1.46) | 47 (22.82) | 153 (74.27) | 3 (1.46) | 203 | 7.45 (1.49) | 8 (7 to 9); (1, 9) |
|  | 1* | 0 | 1 (0.68) | 32 (21.92) | 112 (76.71) | 1 (0.68) | 145 | 7.55 (1.31) | 8 (7 to 9); (3, 9) |
|  | 2 | 0 | 0 (0) | 19 (12.58) | 132 (87.42) | 0 (0) | 151 | 7.84 (1.14) | 8 (7 to 9); (4, 9) |
|  | 2* | 0 | 2 (0.95) | 34 (16.19) | 172 (81.9) | 2 (0.95) | 208 | 7.65 (1.4) | 8 (7 to 9); (1, 9) |
| CONSORT | 1 | 0 | 7 (3.4) | 77 (37.38) | 119 (57.77) | 3 (1.46) | 203 | 6.85 (1.73) | 7 (6 to 8); (1, 9) |
|  | 1* | 0 | 3 (2.05) | 55 (37.67) | 87 (59.59) | 1 (0.68) | 145 | 6.95 (1.59) | 7 (6 to 8); (1, 9) |
|  | 2 | 0 | 1 (0.66) | 43 (28.48) | 107 (70.86) | 0 (0) | 151 | 7.29 (1.33) | 7 (6 to 9); (3, 9) |
|  | 2* | 0 | 5 (2.38) | 65 (30.95) | 138 (65.71) | 2 (0.95) | 208 | 7.09 (1.58) | 7 (6 to 9); (1, 9) |
| [5.6/N*] Summary of findings from existing correlative biomarker, correlative and associated studies to support planned biomarker sub-study (if applicable) | | | | | | | | | |
| SPIRIT | 1 | 0 | 5 (2.43) | 79 (38.35) | 118 (57.28) | 4 (1.94) | 202 | 6.82 (1.64) | 7 (6 to 8); (1, 9) |
|  | 1* | 0 | 2 (1.37) | 54 (36.99) | 88 (60.27) | 2 (1.37) | 144 | 6.93 (1.53) | 7 (6 to 8); (3, 9) |
|  | 2 | 0 | 0 (0) | 68 (45.03) | 82 (54.3) | 1 (0.66) | 150 | 6.88 (1.24) | 7 (6 to 8); (4, 9) |
|  | 2* | 0 | 3 (1.43) | 93 (44.29) | 111 (52.86) | 3 (1.43) | 207 | 6.79 (1.45) | 7 (6 to 8); (1, 9) |
| CONSORT | 1 | 0 | 12 (5.83) | 100 (48.54) | 90 (43.69) | 4 (1.94) | 202 | 6.34 (1.78) | 6 (5 to 8); (1, 9) |
|  | 1* | 0 | 8 (5.48) | 68 (46.58) | 68 (46.58) | 2 (1.37) | 144 | 6.4 (1.73) | 6 (5.75 to 8); (1, 9) |
|  | 2 | 0 | 0 (0) | 93 (61.59) | 57 (37.75) | 1 (0.66) | 150 | 6.45 (1.26) | 6 (6 to 7); (4, 9) |
|  | 2* | 0 | 4 (1.9) | 125 (59.52) | 78 (37.14) | 3 (1.43) | 207 | 6.38 (1.48) | 6 (6 to 7); (1, 9) |
| [7.8/M] Specific objectives (e.g., safety, activity, pharmacokinetics, pharmacodynamics) or hypotheses | | | | | | | | | |
| SPIRIT | 1 | 0 | 1 (0.49) | 5 (2.43) | 200 (97.09) | 0 (0) | 206 | 8.49 (0.97) | 9 (8 to 9); (1, 9) |
|  | 1* | 0 | 1 (0.68) | 2 (1.37) | 143 (97.95) | 0 (0) | 146 | 8.55 (0.95) | 9 (8 to 9); (1, 9) |
|  | 2 | 0 | 1 (0.66) | 0 (0) | 150 (99.34) | 0 (0) | 151 | 8.79 (0.77) | 9 (9 to 9); (1, 9) |
|  | 2* | 0 | 1 (0.48) | 3 (1.43) | 206 (98.1) | 0 (0) | 210 | 8.67 (0.87) | 9 (9 to 9); (1, 9) |
| CONSORT | 1 | 0 | 1 (0.49) | 6 (2.91) | 199 (96.6) | 0 (0) | 206 | 8.45 (1) | 9 (8 to 9); (1, 9) |
|  | 1* | 0 | 1 (0.68) | 2 (1.37) | 143 (97.95) | 0 (0) | 146 | 8.51 (0.96) | 9 (8 to 9); (1, 9) |
|  | 2 | 0 | 1 (0.66) | 0 (0) | 150 (99.34) | 0 (0) | 151 | 8.77 (0.78) | 9 (9 to 9); (1, 9) |
|  | 2* | 0 | 1 (0.48) | 4 (1.9) | 205 (97.62) | 0 (0) | 210 | 8.64 (0.9) | 9 (9 to 9); (1, 9) |
| [9.10/N] Trial design schema (to show flow of decision points, e.g., dose escalation to expansion) | | | | | | | | | |
| SPIRIT | 1 | 0 | 0 (0) | 20 (9.71) | 186 (90.29) | 0 (0) | 206 | 8.09 (1.15) | 8.5 (7 to 9); (4, 9) |
|  | 1* | 0 | 0 (0) | 17 (11.64) | 129 (88.36) | 0 (0) | 146 | 8.07 (1.2) | 9 (7 to 9); (4, 9) |
|  | 2 | 0 | 0 (0) | 6 (3.97) | 145 (96.03) | 0 (0) | 151 | 8.51 (0.92) | 9 (8 to 9); (4, 9) |
|  | 2* | 0 | 0 (0) | 9 (4.29) | 201 (95.71) | 0 (0) | 210 | 8.4 (0.97) | 9 (8 to 9); (4, 9) |
| CONSORT | 1 | 0 | 1 (0.49) | 27 (13.11) | 178 (86.41) | 0 (0) | 206 | 7.83 (1.36) | 8 (7 to 9); (2, 9) |
|  | 1* | 0 | 1 (0.68) | 22 (15.07) | 123 (84.25) | 0 (0) | 146 | 7.75 (1.42) | 8 (7 to 9); (2, 9) |
|  | 2 | 0 | 0 (0) | 10 (6.62) | 141 (93.38) | 0 (0) | 151 | 8.32 (1.13) | 9 (8 to 9); (4, 9) |
|  | 2* | 0 | 0 (0) | 15 (7.14) | 195 (92.86) | 0 (0) | 210 | 8.23 (1.15) | 9 (8 to 9); (4, 9) |
| [11.12/N*] Statistical methodology or rationale underpinning the trial design | | | | | | | | | |
| SPIRIT | 1 | 0 | 1 (0.49) | 27 (13.11) | 177 (85.92) | 1 (0.49) | 205 | 7.92 (1.36) | 8 (7 to 9); (1, 9) |
|  | 1* | 0 | 1 (0.68) | 16 (10.96) | 129 (88.36) | 0 (0) | 146 | 8.03 (1.3) | 8 (8 to 9); (1, 9) |
|  | 2 | 0 | 1 (0.66) | 4 (2.65) | 146 (96.69) | 0 (0) | 151 | 8.51 (1.01) | 9 (8 to 9); (1, 9) |
|  | 2* | 0 | 1 (0.48) | 15 (7.14) | 193 (91.9) | 1 (0.48) | 209 | 8.26 (1.22) | 9 (8 to 9); (1, 9) |
| CONSORT | 1 | 0 | 1 (0.49) | 34 (16.5) | 170 (82.52) | 1 (0.49) | 205 | 7.7 (1.39) | 8 (7 to 9); (1, 9) |
|  | 1* | 0 | 1 (0.68) | 23 (15.75) | 122 (83.56) | 0 (0) | 146 | 7.79 (1.38) | 8 (7 to 9); (1, 9) |
|  | 2 | 0 | 1 (0.66) | 9 (5.96) | 141 (93.38) | 0 (0) | 151 | 8.36 (1.1) | 9 (8 to 9); (1, 9) |
|  | 2* | 0 | 1 (0.48) | 20 (9.52) | 188 (89.52) | 1 (0.48) | 209 | 8.11 (1.26) | 9 (8 to 9); (1, 9) |
| [13.14/N] Starting dose(s) specification with rationale | | | | | | | | | |
| SPIRIT | 1 | 0 | 0 (0) | 14 (6.8) | 191 (92.72) | 1 (0.49) | 205 | 8.22 (1.06) | 9 (8 to 9); (4, 9) |
|  | 1* | 0 | 0 (0) | 9 (6.16) | 137 (93.84) | 0 (0) | 146 | 8.28 (1.03) | 9 (8 to 9); (4, 9) |
|  | 2 | 0 | 0 (0) | 2 (1.32) | 149 (98.68) | 0 (0) | 151 | 8.61 (0.76) | 9 (8.5 to 9); (5, 9) |
|  | 2* | 0 | 0 (0) | 7 (3.33) | 202 (96.19) | 1 (0.48) | 209 | 8.45 (0.91) | 9 (8 to 9); (5, 9) |
| CONSORT | 1 | 0 | 4 (1.94) | 24 (11.65) | 177 (85.92) | 1 (0.49) | 205 | 7.77 (1.45) | 8 (7 to 9); (1, 9) |
|  | 1* | 0 | 2 (1.37) | 17 (11.64) | 127 (86.99) | 0 (0) | 146 | 7.86 (1.4) | 8 (7 to 9); (1, 9) |
|  | 2 | 0 | 1 (0.66) | 7 (4.64) | 143 (94.7) | 0 (0) | 151 | 8.31 (1.11) | 9 (8 to 9); (2, 9) |
|  | 2* | 0 | 3 (1.43) | 14 (6.67) | 192 (91.43) | 1 (0.48) | 209 | 8.09 (1.3) | 9 (7 to 9); (2, 9) |
| [15.16/N*] Dosing regimens (e.g., doses/schedules or intensity of fractionation) considered with rationale | | | | | | | | | |
| SPIRIT | 1 | 0 | 0 (0) | 20 (9.71) | 186 (90.29) | 0 (0) | 206 | 8.05 (1.1) | 8 (7 to 9); (4, 9) |
|  | 1* | 0 | 0 (0) | 14 (9.59) | 132 (90.41) | 0 (0) | 146 | 8.12 (1.08) | 8.5 (7 to 9); (4, 9) |
|  | 2 | 0 | 0 (0) | 6 (3.97) | 145 (96.03) | 0 (0) | 151 | 8.42 (0.95) | 9 (8 to 9); (4, 9) |
|  | 2* | 0 | 0 (0) | 12 (5.71) | 198 (94.29) | 0 (0) | 210 | 8.26 (1.03) | 9 (7 to 9); (4, 9) |
| CONSORT | 1 | 0 | 3 (1.46) | 31 (15.05) | 172 (83.5) | 0 (0) | 206 | 7.64 (1.44) | 8 (7 to 9); (1, 9) |
|  | 1* | 0 | 2 (1.37) | 21 (14.38) | 123 (84.25) | 0 (0) | 146 | 7.71 (1.43) | 8 (7 to 9); (1, 9) |
|  | 2 | 0 | 1 (0.66) | 8 (5.3) | 142 (94.04) | 0 (0) | 151 | 8.15 (1.13) | 9 (7 to 9); (2, 9) |
|  | 2* | 0 | 2 (0.95) | 18 (8.57) | 190 (90.48) | 0 (0) | 210 | 7.95 (1.27) | 8 (7 to 9); (2, 9) |
| [17.18/N*] Planned (and delivered) dosing regimens presented as a diagram or table, where applicable | | | | | | | | | |
| SPIRIT | 1 | 0 | 6 (2.91) | 81 (39.32) | 117 (56.8) | 2 (0.97) | 204 | 6.88 (1.67) | 7 (6 to 8); (1, 9) |
|  | 1* | 0 | 5 (3.42) | 58 (39.73) | 82 (56.16) | 1 (0.68) | 145 | 6.91 (1.67) | 7 (6 to 8); (1, 9) |
|  | 2 | 0 | 3 (1.99) | 52 (34.44) | 96 (63.58) | 0 (0) | 151 | 7.17 (1.6) | 7 (6 to 9); (1, 9) |
|  | 2* | 0 | 4 (1.9) | 75 (35.71) | 130 (61.9) | 1 (0.48) | 209 | 7.07 (1.63) | 7 (6 to 9); (1, 9) |
| CONSORT | 1 | 0 | 8 (3.88) | 84 (40.78) | 112 (54.37) | 2 (0.97) | 204 | 6.7 (1.72) | 7 (5 to 8); (1, 9) |
|  | 1* | 0 | 7 (4.79) | 62 (42.47) | 76 (52.05) | 1 (0.68) | 145 | 6.68 (1.76) | 7 (5 to 8); (1, 9) |
|  | 2 | 0 | 3 (1.99) | 57 (37.75) | 91 (60.26) | 0 (0) | 151 | 6.97 (1.6) | 7 (6 to 8); (1, 9) |
|  | 2* | 0 | 4 (1.9) | 79 (37.62) | 126 (60) | 1 (0.48) | 209 | 6.91 (1.62) | 7 (6 to 8); (1, 9) |
| [19.20/N*] Specify whether skipping of dose level is permissible, if applicable | | | | | | | | | |
| SPIRIT | 1 | 0 | 5 (2.43) | 46 (22.33) | 145 (70.39) | 10 (4.85) | 196 | 7.37 (1.59) | 7.5 (6 to 9); (1, 9) |
|  | 1* | 0 | 3 (2.05) | 30 (20.55) | 107 (73.29) | 6 (4.11) | 140 | 7.44 (1.57) | 8 (7 to 9); (1, 9) |
|  | 2 | 0 | 1 (0.66) | 18 (11.92) | 130 (86.09) | 2 (1.32) | 149 | 7.89 (1.32) | 8 (7 to 9); (2, 9) |
|  | 2* | 0 | 3 (1.43) | 34 (16.19) | 167 (79.52) | 6 (2.86) | 204 | 7.7 (1.45) | 8 (7 to 9); (2, 9) |
| CONSORT | 1 | 0 | 9 (4.37) | 57 (27.67) | 130 (63.11) | 10 (4.85) | 196 | 7.01 (1.72) | 7 (6 to 9); (1, 9) |
|  | 1* | 0 | 7 (4.79) | 37 (25.34) | 96 (65.75) | 6 (4.11) | 140 | 7.04 (1.75) | 7 (6 to 9); (1, 9) |
|  | 2 | 0 | 4 (2.65) | 26 (17.22) | 119 (78.81) | 2 (1.32) | 149 | 7.42 (1.59) | 7 (7 to 9); (2, 9) |
|  | 2* | 0 | 6 (2.86) | 46 (21.9) | 152 (72.38) | 6 (2.86) | 204 | 7.28 (1.62) | 7 (6 to 9); (2, 9) |
| [21.22/N] Planned cohort size(s) (fixed or flexible) | | | | | | | | | |
| SPIRIT | 1 | 0 | 1 (0.49) | 29 (14.08) | 176 (85.44) | 0 (0) | 206 | 7.83 (1.27) | 8 (7 to 9); (3, 9) |
|  | 1* | 0 | 1 (0.68) | 19 (13.01) | 126 (86.3) | 0 (0) | 146 | 7.88 (1.29) | 8 (7 to 9); (3, 9) |
|  | 2 | 0 | 1 (0.66) | 9 (5.96) | 141 (93.38) | 0 (0) | 151 | 8.29 (1.1) | 9 (7 to 9); (3, 9) |
|  | 2* | 0 | 1 (0.48) | 19 (9.05) | 190 (90.48) | 0 (0) | 210 | 8.12 (1.17) | 9 (7 to 9); (3, 9) |
| CONSORT | 1 | 0 | 3 (1.46) | 38 (18.45) | 165 (80.1) | 0 (0) | 206 | 7.54 (1.43) | 8 (7 to 9); (2, 9) |
|  | 1* | 0 | 3 (2.05) | 22 (15.07) | 121 (82.88) | 0 (0) | 146 | 7.58 (1.44) | 8 (7 to 9); (2, 9) |
|  | 2 | 0 | 2 (1.32) | 11 (7.28) | 138 (91.39) | 0 (0) | 151 | 8.01 (1.3) | 9 (7 to 9); (2, 9) |
|  | 2* | 0 | 2 (0.95) | 27 (12.86) | 181 (86.19) | 0 (0) | 210 | 7.85 (1.35) | 8 (7 to 9); (2, 9) |
| [23.24/N] Sequence and interval between dosing of participants | | | | | | | | | |
| SPIRIT | 1 | 0 | 1 (0.49) | 38 (18.45) | 166 (80.58) | 1 (0.49) | 205 | 7.7 (1.41) | 8 (7 to 9); (3, 9) |
|  | 1* | 0 | 1 (0.68) | 27 (18.49) | 117 (80.14) | 1 (0.68) | 145 | 7.79 (1.42) | 8 (7 to 9); (3, 9) |
|  | 2 | 0 | 1 (0.66) | 12 (7.95) | 137 (90.73) | 1 (0.66) | 150 | 8.24 (1.15) | 9 (8 to 9); (3, 9) |
|  | 2* | 0 | 1 (0.48) | 23 (10.95) | 185 (88.1) | 1 (0.48) | 209 | 8.03 (1.26) | 9 (7 to 9); (3, 9) |
| CONSORT | 1 | 0 | 3 (1.46) | 64 (31.07) | 138 (66.99) | 1 (0.49) | 205 | 7.2 (1.56) | 7 (6 to 9); (2, 9) |
|  | 1* | 0 | 3 (2.05) | 46 (31.51) | 96 (65.75) | 1 (0.68) | 145 | 7.21 (1.6) | 8 (6 to 9); (2, 9) |
|  | 2 | 0 | 2 (1.32) | 25 (16.56) | 123 (81.46) | 1 (0.66) | 150 | 7.8 (1.37) | 8 (7 to 9); (2, 9) |
|  | 2* | 0 | 2 (0.95) | 43 (20.48) | 164 (78.1) | 1 (0.48) | 209 | 7.63 (1.43) | 8 (7 to 9); (2, 9) |
| [25.26/N*] Pre-planned interim decision-making criteria/rules to guide the trial adaptation process (e.g., dosing decision to [de-]escalate), pre-planned timing and frequency of interim data looks and the information to inform the adaptations. Alternatively, an explanation of why they are not pre-specified | | | | | | | | | |
| SPIRIT | 1 | 0 | 1 (0.49) | 23 (11.17) | 182 (88.35) | 0 (0) | 206 | 8.03 (1.3) | 8 (7 to 9); (1, 9) |
|  | 1* | 0 | 0 (0) | 12 (8.22) | 134 (91.78) | 0 (0) | 146 | 8.21 (1.1) | 9 (8 to 9); (4, 9) |
|  | 2 | 0 | 0 (0) | 3 (1.99) | 148 (98.01) | 0 (0) | 151 | 8.58 (0.83) | 9 (8 to 9); (4, 9) |
|  | 2* | 0 | 1 (0.48) | 14 (6.67) | 195 (92.86) | 0 (0) | 210 | 8.29 (1.19) | 9 (8 to 9); (1, 9) |
| CONSORT | 1 | 0 | 5 (2.43) | 45 (21.84) | 156 (75.73) | 0 (0) | 206 | 7.51 (1.65) | 8 (7 to 9); (1, 9) |
|  | 1* | 0 | 3 (2.05) | 29 (19.86) | 114 (78.08) | 0 (0) | 146 | 7.67 (1.57) | 8 (7 to 9); (2, 9) |
|  | 2 | 0 | 2 (1.32) | 8 (5.3) | 141 (93.38) | 0 (0) | 151 | 8.28 (1.2) | 9 (8 to 9); (2, 9) |
|  | 2* | 0 | 4 (1.9) | 24 (11.43) | 182 (86.67) | 0 (0) | 210 | 7.94 (1.48) | 9 (7 to 9); (1, 9) |
| [27.28/N] Trial stopping criteria and consequences | | | | | | | | | |
| SPIRIT | 1 | 0 | 2 (0.97) | 19 (9.22) | 185 (89.81) | 0 (0) | 206 | 8.11 (1.28) | 9 (8 to 9); (1, 9) |
|  | 1* | 0 | 1 (0.68) | 11 (7.53) | 134 (91.78) | 0 (0) | 146 | 8.25 (1.21) | 9 (8 to 9); (1, 9) |
|  | 2 | 0 | 1 (0.66) | 5 (3.31) | 145 (96.03) | 0 (0) | 151 | 8.58 (1.01) | 9 (8.5 to 9); (1, 9) |
|  | 2* | 0 | 2 (0.95) | 13 (6.19) | 195 (92.86) | 0 (0) | 210 | 8.34 (1.19) | 9 (8 to 9); (1, 9) |
| CONSORT | 1 | 0 | 4 (1.94) | 38 (18.45) | 164 (79.61) | 0 (0) | 206 | 7.62 (1.54) | 8 (7 to 9); (1, 9) |
|  | 1* | 0 | 3 (2.05) | 24 (16.44) | 119 (81.51) | 0 (0) | 146 | 7.71 (1.53) | 8 (7 to 9); (1, 9) |
|  | 2 | 0 | 1 (0.66) | 9 (5.96) | 141 (93.38) | 0 (0) | 151 | 8.26 (1.2) | 9 (8 to 9); (1, 9) |
|  | 2* | 0 | 2 (0.95) | 23 (10.95) | 185 (88.1) | 0 (0) | 210 | 8.01 (1.37) | 9 (7 to 9); (1, 9) |
| [29.30/N*] Dose expansion cohort(s), if applicable | | | | | | | | | |
| SPIRIT | 1 | 0 | 1 (0.49) | 41 (19.9) | 159 (77.18) | 5 (2.43) | 201 | 7.67 (1.4) | 8 (7 to 9); (3, 9) |
|  | 1* | 0 | 0 (0) | 27 (18.49) | 116 (79.45) | 3 (2.05) | 143 | 7.76 (1.35) | 8 (7 to 9); (4, 9) |
|  | 2 | 0 | 0 (0) | 14 (9.27) | 135 (89.4) | 2 (1.32) | 149 | 8.15 (1.13) | 9 (8 to 9); (4, 9) |
|  | 2* | 0 | 1 (0.48) | 28 (13.33) | 177 (84.29) | 4 (1.9) | 206 | 7.96 (1.27) | 8 (7 to 9); (3, 9) |
| CONSORT | 1 | 0 | 1 (0.49) | 52 (25.24) | 148 (71.84) | 5 (2.43) | 201 | 7.41 (1.39) | 8 (6 to 9); (3, 9) |
|  | 1* | 0 | 0 (0) | 37 (25.34) | 106 (72.6) | 3 (2.05) | 143 | 7.45 (1.39) | 8 (6 to 9); (4, 9) |
|  | 2 | 0 | 0 (0) | 19 (12.58) | 130 (86.09) | 2 (1.32) | 149 | 7.94 (1.18) | 8 (7 to 9); (4, 9) |
|  | 2* | 0 | 1 (0.48) | 34 (16.19) | 171 (81.43) | 4 (1.9) | 206 | 7.76 (1.27) | 8 (7 to 9); (3, 9) |
| [31/M] Important changes to the design or methods after trial commencement that are relevant for dose determination outside the scope of the pre-planned adaptive design features, with reasons | | | | | | | | | |
| CONSORT | 1 | 0 | 2 (0.97) | 24 (11.65) | 178 (86.41) | 2 (0.97) | 204 | 7.98 (1.28) | 8 (7 to 9); (3, 9) |
|  | 1* | 0 | 0 (0) | 13 (8.9) | 131 (89.73) | 2 (1.37) | 144 | 8.12 (1.08) | 8 (8 to 9); (5, 9) |
|  | 2 | 0 | 0 (0) | 4 (2.65) | 146 (96.69) | 1 (0.66) | 150 | 8.55 (0.8) | 9 (8 to 9); (5, 9) |
|  | 2* | 0 | 2 (0.95) | 15 (7.14) | 192 (91.43) | 1 (0.48) | 209 | 8.28 (1.18) | 9 (8 to 9); (3, 9) |
| [32.33/M] The interventions for each dose level (and within each treatment group for randomised early phase trials, if applicable) | | | | | | | | | |
| SPIRIT | 1 | 9 | 1 (0.51) | 25 (12.69) | 170 (86.29) | 1 (0.51) | 196 | 7.87 (1.29) | 8 (7 to 9); (3, 9) |
|  | 1* | 2 | 1 (0.69) | 17 (11.81) | 125 (86.81) | 1 (0.69) | 143 | 7.94 (1.27) | 8 (7 to 9); (3, 9) |
|  | 2 | 1 | 0 (0) | 6 (4) | 143 (95.33) | 1 (0.67) | 149 | 8.36 (0.99) | 9 (8 to 9); (4, 9) |
|  | 2* | 7 | 0 (0) | 14 (6.9) | 188 (92.61) | 1 (0.49) | 202 | 8.17 (1.13) | 9 (7 to 9); (4, 9) |
| CONSORT | 1 | 9 | 3 (1.52) | 38 (19.29) | 155 (78.68) | 1 (0.51) | 196 | 7.5 (1.48) | 8 (7 to 9); (3, 9) |
|  | 1* | 2 | 3 (2.08) | 25 (17.36) | 115 (79.86) | 1 (0.69) | 143 | 7.58 (1.48) | 8 (7 to 9); (3, 9) |
|  | 2 | 1 | 1 (0.67) | 8 (5.33) | 140 (93.33) | 1 (0.67) | 149 | 8.16 (1.13) | 9 (7 to 9); (3, 9) |
|  | 2* | 7 | 1 (0.49) | 22 (10.84) | 179 (88.18) | 1 (0.49) | 202 | 7.92 (1.29) | 8 (7 to 9); (3, 9) |
| [34.35/M] Criteria for discontinuing, dose modifications and dosing delays of allocated interventions for a given trial participant | | | | | | | | | |
| SPIRIT | 1 | 9 | 1 (0.51) | 26 (13.2) | 170 (86.29) | 0 (0) | 197 | 7.93 (1.23) | 8 (7 to 9); (2, 9) |
|  | 1* | 2 | 1 (0.69) | 17 (11.81) | 126 (87.5) | 0 (0) | 144 | 7.97 (1.27) | 8 (7 to 9); (2, 9) |
|  | 2 | 1 | 1 (0.67) | 5 (3.33) | 144 (96) | 0 (0) | 150 | 8.39 (1.06) | 9 (8 to 9); (2, 9) |
|  | 2* | 7 | 1 (0.49) | 14 (6.9) | 188 (92.61) | 0 (0) | 203 | 8.24 (1.1) | 9 (8 to 9); (2, 9) |
| CONSORT | 1 | 9 | 2 (1.02) | 59 (29.95) | 136 (69.04) | 0 (0) | 197 | 7.29 (1.48) | 7 (6 to 9); (3, 9) |
|  | 1* | 2 | 2 (1.39) | 39 (27.08) | 103 (71.53) | 0 (0) | 144 | 7.35 (1.52) | 7 (6 to 9); (3, 9) |
|  | 2 | 1 | 2 (1.33) | 15 (10) | 133 (88.67) | 0 (0) | 150 | 7.92 (1.29) | 8 (7 to 9); (3, 9) |
|  | 2* | 7 | 2 (0.99) | 36 (17.73) | 165 (81.28) | 0 (0) | 203 | 7.7 (1.35) | 8 (7 to 9); (3, 9) |
| [36.37/M] Define pre-specified primary and secondary outcome measures, including how and when they were assessed. Any other outcome measures used to inform pre-planned adaptations should be described with the rationale | | | | | | | | | |
| SPIRIT | 1 | 9 | 1 (0.51) | 20 (10.15) | 176 (89.34) | 0 (0) | 197 | 8.11 (1.22) | 9 (8 to 9); (3, 9) |
|  | 1* | 2 | 1 (0.69) | 14 (9.72) | 129 (89.58) | 0 (0) | 144 | 8.17 (1.25) | 9 (8 to 9); (3, 9) |
|  | 2 | 1 | 1 (0.67) | 3 (2) | 146 (97.33) | 0 (0) | 150 | 8.63 (0.89) | 9 (9 to 9); (3, 9) |
|  | 2* | 7 | 1 (0.49) | 9 (4.43) | 193 (95.07) | 0 (0) | 203 | 8.46 (1.01) | 9 (8 to 9); (3, 9) |
| CONSORT | 1 | 9 | 2 (1.02) | 26 (13.2) | 169 (85.79) | 0 (0) | 197 | 7.88 (1.37) | 8 (7 to 9); (3, 9) |
|  | 1* | 2 | 2 (1.39) | 15 (10.42) | 127 (88.19) | 0 (0) | 144 | 7.98 (1.36) | 8 (7 to 9); (3, 9) |
|  | 2 | 1 | 2 (1.33) | 4 (2.67) | 144 (96) | 0 (0) | 150 | 8.51 (1.04) | 9 (8 to 9); (3, 9) |
|  | 2* | 7 | 2 (0.99) | 15 (7.39) | 186 (91.63) | 0 (0) | 203 | 8.27 (1.21) | 9 (8 to 9); (3, 9) |
| [38.39/M*] Estimated number of participants (minimum/lower bound, maximum or expected range) needed to address study objectives and how it was determined, including clinical and statistical assumptions supporting any sample size AND operating characteristics | | | | | | | | | |
| SPIRIT | 1 | 9 | 5 (2.54) | 34 (17.26) | 158 (80.2) | 0 (0) | 197 | 7.64 (1.58) | 8 (7 to 9); (1, 9) |
|  | 1* | 2 | 3 (2.08) | 22 (15.28) | 119 (82.64) | 0 (0) | 144 | 7.76 (1.57) | 8 (7 to 9); (1, 9) |
|  | 2 | 1 | 1 (0.67) | 12 (8) | 137 (91.33) | 0 (0) | 150 | 8.27 (1.23) | 9 (8 to 9); (1, 9) |
|  | 2* | 7 | 3 (1.48) | 24 (11.82) | 176 (86.7) | 0 (0) | 203 | 8.02 (1.38) | 9 (7 to 9); (1, 9) |
| CONSORT | 1 | 9 | 7 (3.55) | 51 (25.89) | 139 (70.56) | 0 (0) | 197 | 7.2 (1.72) | 7 (6 to 9); (1, 9) |
|  | 1* | 2 | 4 (2.78) | 36 (25) | 104 (72.22) | 0 (0) | 144 | 7.31 (1.7) | 8 (6 to 9); (1, 9) |
|  | 2 | 1 | 2 (1.33) | 22 (14.67) | 126 (84) | 0 (0) | 150 | 7.89 (1.43) | 8 (7 to 9); (1, 9) |
|  | 2* | 7 | 5 (2.46) | 38 (18.72) | 160 (78.82) | 0 (0) | 203 | 7.62 (1.57) | 8 (7 to 9); (1, 9) |
| [40/M] Time schedule of enrolment, interventions (including any run-ins and washouts), assessments, and visits for participants (including in-house stay or out-patient follow-up period where applicable). A schematic diagram is highly recommended | | | | | | | | | |
| SPIRIT | 1 | 9 | 6 (3.05) | 40 (20.3) | 151 (76.65) | 0 (0) | 197 | 7.44 (1.56) | 8 (7 to 9); (2, 9) |
|  | 1* | 2 | 3 (2.08) | 30 (20.83) | 111 (77.08) | 0 (0) | 144 | 7.49 (1.48) | 8 (7 to 9); (2, 9) |
|  | 2 | 1 | 2 (1.33) | 15 (10) | 133 (88.67) | 0 (0) | 150 | 7.93 (1.31) | 8 (7 to 9); (2, 9) |
|  | 2* | 7 | 5 (2.46) | 25 (12.32) | 173 (85.22) | 0 (0) | 203 | 7.77 (1.46) | 8 (7 to 9); (2, 9) |
| [41/N] Plans for recruitment/screening slots for sequential cohorts of participants | | | | | | | | | |
| SPIRIT | 1 | 9 | 20 (10.15) | 92 (46.7) | 81 (41.12) | 4 (2.03) | 193 | 6.11 (1.78) | 6 (5 to 7); (1, 9) |
|  | 1* | 2 | 10 (6.94) | 64 (44.44) | 67 (46.53) | 3 (2.08) | 141 | 6.3 (1.68) | 6 (5 to 7); (1, 9) |
|  | 2 | 1 | 7 (4.67) | 84 (56) | 56 (37.33) | 3 (2) | 147 | 6.24 (1.32) | 6 (6 to 7); (2, 9) |
|  | 2* | 7 | 17 (8.37) | 111 (54.68) | 71 (34.98) | 4 (1.97) | 199 | 6.09 (1.55) | 6 (6 to 7); (1, 9) |
| [42.43/N] Any pre-planned allocation rule or algorithm to update randomisation with timing and frequency of updates | | | | | | | | | |
| SPIRIT | 1 | 9 | 5 (2.54) | 43 (21.83) | 143 (72.59) | 6 (3.05) | 191 | 7.35 (1.57) | 8 (6.5 to 9); (2, 9) |
|  | 1* | 2 | 1 (0.69) | 28 (19.44) | 112 (77.78) | 3 (2.08) | 141 | 7.52 (1.44) | 8 (7 to 9); (3, 9) |
|  | 2 | 1 | 0 (0) | 20 (13.33) | 129 (86) | 1 (0.67) | 149 | 7.82 (1.3) | 8 (7 to 9); (4, 9) |
|  | 2* | 7 | 4 (1.97) | 35 (17.24) | 160 (78.82) | 4 (1.97) | 199 | 7.58 (1.5) | 8 (7 to 9); (2, 9) |
| CONSORT | 1 | 9 | 8 (4.06) | 62 (31.47) | 122 (61.93) | 5 (2.54) | 192 | 6.94 (1.64) | 7 (6 to 8); (2, 9) |
|  | 1* | 2 | 3 (2.08) | 44 (30.56) | 95 (65.97) | 2 (1.39) | 142 | 7.07 (1.52) | 7 (6 to 8); (3, 9) |
|  | 2 | 1 | 2 (1.33) | 25 (16.67) | 122 (81.33) | 1 (0.67) | 149 | 7.4 (1.33) | 7 (7 to 9); (3, 9) |
|  | 2* | 7 | 7 (3.45) | 43 (21.18) | 149 (73.4) | 4 (1.97) | 199 | 7.19 (1.53) | 7 (6.5 to 8); (2, 9) |
| [44/N] Any changes to the allocation rule after trial adaptation decisions | | | | | | | | | |
| CONSORT | 1 | 9 | 5 (2.54) | 42 (21.32) | 145 (73.6) | 5 (2.54) | 192 | 7.44 (1.53) | 8 (7 to 9); (2, 9) |
|  | 1* | 2 | 1 (0.69) | 31 (21.53) | 109 (75.69) | 3 (2.08) | 141 | 7.51 (1.41) | 8 (7 to 9); (3, 9) |
|  | 2 | 1 | 1 (0.67) | 17 (11.33) | 131 (87.33) | 1 (0.67) | 149 | 7.95 (1.26) | 9 (7 to 9); (3, 9) |
|  | 2* | 7 | 5 (2.46) | 28 (13.79) | 167 (82.27) | 3 (1.48) | 200 | 7.76 (1.46) | 8 (7 to 9); (2, 9) |
| [45/M] Specify if the data management plans in the initial dose-finding component are different from subsequent stages (e.g., expansion cohort(s) or Phase II) of the trial | | | | | | | | | |
| SPIRIT | 1 | 9 | 21 (10.66) | 89 (45.18) | 83 (42.13) | 4 (2.03) | 193 | 6.05 (1.95) | 6 (5 to 7); (1, 9) |
|  | 1* | 2 | 10 (6.94) | 69 (47.92) | 61 (42.36) | 4 (2.78) | 140 | 6.14 (1.82) | 6 (5 to 7); (1, 9) |
|  | 2 | 1 | 9 (6) | 83 (55.33) | 57 (38) | 1 (0.67) | 149 | 6.1 (1.58) | 6 (5 to 7); (1, 9) |
|  | 2* | 7 | 20 (9.85) | 104 (51.23) | 78 (38.42) | 1 (0.49) | 202 | 6 (1.78) | 6 (5 to 7); (1, 9) |
| [46.47/M*] For the proposed adaptive design features, statistical methods used to estimate key endpoints (e.g., safety, dose(s), treatment effects) | | | | | | | | | |
| SPIRIT | 1 | 9 | 2 (1.02) | 32 (16.24) | 162 (82.23) | 1 (0.51) | 196 | 7.61 (1.48) | 8 (7 to 9); (2, 9) |
|  | 1* | 2 | 0 (0) | 18 (12.5) | 126 (87.5) | 0 (0) | 144 | 7.83 (1.31) | 8 (7 to 9); (4, 9) |
|  | 2 | 1 | 0 (0) | 6 (4) | 144 (96) | 0 (0) | 150 | 8.28 (1.02) | 9 (8 to 9); (4, 9) |
|  | 2* | 7 | 2 (0.99) | 20 (9.85) | 180 (88.67) | 1 (0.49) | 202 | 7.96 (1.36) | 9 (7 to 9); (2, 9) |
| CONSORT | 1 | 9 | 3 (1.52) | 32 (16.24) | 161 (81.73) | 1 (0.51) | 196 | 7.56 (1.41) | 8 (7 to 9); (2, 9) |
|  | 1* | 2 | 0 (0) | 19 (13.19) | 125 (86.81) | 0 (0) | 144 | 7.75 (1.25) | 8 (7 to 9); (4, 9) |
|  | 2 | 1 | 0 (0) | 7 (4.67) | 143 (95.33) | 0 (0) | 150 | 8.15 (1.07) | 9 (7 to 9); (4, 9) |
|  | 2* | 7 | 3 (1.48) | 20 (9.85) | 179 (88.18) | 1 (0.49) | 202 | 7.87 (1.35) | 8 (7 to 9); (2, 9) |
| [48.49/M] Statistical methods for additional analyses (e.g., subgroup and adjusted analyses, PK/PD, biomarker correlative analyses) | | | | | | | | | |
| SPIRIT | 1 | 9 | 8 (4.06) | 80 (40.61) | 106 (53.81) | 3 (1.52) | 194 | 6.59 (1.64) | 7 (6 to 8); (1, 9) |
|  | 1* | 2 | 4 (2.78) | 52 (36.11) | 86 (59.72) | 2 (1.39) | 142 | 6.77 (1.58) | 7 (6 to 8); (1, 9) |
|  | 2 | 1 | 1 (0.67) | 45 (30) | 104 (69.33) | 0 (0) | 150 | 6.9 (1.2) | 7 (6 to 7); (1, 9) |
|  | 2* | 7 | 5 (2.46) | 73 (35.96) | 123 (60.59) | 2 (0.99) | 201 | 6.68 (1.38) | 7 (6 to 7); (1, 9) |
| CONSORT | 1 | 9 | 8 (4.06) | 71 (36.04) | 115 (58.38) | 3 (1.52) | 194 | 6.65 (1.57) | 7 (6 to 8); (1, 9) |
|  | 1* | 2 | 4 (2.78) | 49 (34.03) | 89 (61.81) | 2 (1.39) | 142 | 6.82 (1.56) | 7 (6 to 8); (1, 9) |
|  | 2 | 1 | 1 (0.67) | 43 (28.67) | 106 (70.67) | 0 (0) | 150 | 6.89 (1.16) | 7 (6 to 7); (1, 9) |
|  | 2* | 7 | 5 (2.46) | 65 (32.02) | 131 (64.53) | 2 (0.99) | 201 | 6.7 (1.29) | 7 (6 to 7); (1, 9) |
| [50.51/M] Clearly-defined analysis population (e.g., evaluable population for dose determination, safety and key outcomes) | | | | | | | | | |
| SPIRIT | 1 | 9 | 1 (0.51) | 36 (18.27) | 159 (80.71) | 1 (0.51) | 196 | 7.75 (1.42) | 8 (7 to 9); (2, 9) |
|  | 1* | 2 | 1 (0.69) | 21 (14.58) | 122 (84.72) | 0 (0) | 144 | 7.88 (1.38) | 8 (7 to 9); (2, 9) |
|  | 2 | 1 | 1 (0.67) | 6 (4) | 143 (95.33) | 0 (0) | 150 | 8.4 (1.02) | 9 (8 to 9); (2, 9) |
|  | 2* | 7 | 1 (0.49) | 21 (10.34) | 180 (88.67) | 1 (0.49) | 202 | 8.14 (1.23) | 9 (7 to 9); (2, 9) |
| CONSORT | 1 | 9 | 0 (0) | 29 (14.72) | 167 (84.77) | 1 (0.51) | 196 | 7.85 (1.29) | 8 (7 to 9); (4, 9) |
|  | 1* | 2 | 0 (0) | 19 (13.19) | 125 (86.81) | 0 (0) | 144 | 7.91 (1.26) | 8 (7 to 9); (4, 9) |
|  | 2 | 1 | 0 (0) | 7 (4.67) | 143 (95.33) | 0 (0) | 150 | 8.35 (0.96) | 9 (8 to 9); (4, 9) |
|  | 2* | 7 | 0 (0) | 17 (8.37) | 185 (91.13) | 1 (0.49) | 202 | 8.18 (1.12) | 9 (8 to 9); (4, 9) |
| [52.53/N] Pre-specify handling strategies of events occurring after treatment initiation (e.g., how dosing delays will be handled) that affect either the interpretation or the existence of the measurements associated with the clinical question of interest | | | | | | | | | |
| SPIRIT | 1 | 9 | 4 (2.03) | 46 (23.35) | 146 (74.11) | 1 (0.51) | 196 | 7.26 (1.61) | 7 (6 to 9); (1, 9) |
|  | 1* | 2 | 2 (1.39) | 32 (22.22) | 110 (76.39) | 0 (0) | 144 | 7.33 (1.56) | 7 (7 to 9); (1, 9) |
|  | 2 | 1 | 2 (1.33) | 19 (12.67) | 129 (86) | 0 (0) | 150 | 7.69 (1.38) | 8 (7 to 9); (1, 9) |
|  | 2* | 7 | 4 (1.97) | 33 (16.26) | 165 (81.28) | 1 (0.49) | 202 | 7.52 (1.5) | 8 (7 to 9); (1, 9) |
| CONSORT | 1 | 9 | 5 (2.54) | 68 (34.52) | 123 (62.44) | 1 (0.51) | 196 | 6.84 (1.67) | 7 (6 to 8); (1, 9) |
|  | 1* | 2 | 3 (2.08) | 50 (34.72) | 91 (63.19) | 0 (0) | 144 | 6.9 (1.66) | 7 (6 to 8); (1, 9) |
|  | 2 | 1 | 3 (2) | 35 (23.33) | 112 (74.67) | 0 (0) | 150 | 7.15 (1.47) | 7 (6.25 to 8); (1, 9) |
|  | 2* | 7 | 5 (2.46) | 54 (26.6) | 143 (70.44) | 1 (0.49) | 202 | 7 (1.54) | 7 (6 to 8); (1, 9) |
| [54.55/N] Statistical software and packages used for design (e.g., simulation) and to be used for planned analyses | | | | | | | | | |
| SPIRIT | 1 | 9 | 30 (15.23) | 84 (42.64) | 82 (41.62) | 1 (0.51) | 196 | 5.9 (2.1) | 6 (4 to 7); (1, 9) |
|  | 1* | 2 | 18 (12.5) | 63 (43.75) | 63 (43.75) | 0 (0) | 144 | 6.1 (1.98) | 6 (5 to 8); (1, 9) |
|  | 2 | 1 | 12 (8) | 78 (52) | 60 (40) | 0 (0) | 150 | 6.15 (1.63) | 6 (6 to 7); (1, 9) |
|  | 2* | 7 | 25 (12.32) | 99 (48.77) | 78 (38.42) | 1 (0.49) | 202 | 5.92 (1.87) | 6 (5 to 7); (1, 9) |
| CONSORT | 1 | 9 | 23 (11.68) | 85 (43.15) | 88 (44.67) | 1 (0.51) | 196 | 6.1 (2.04) | 6 (5 to 8); (1, 9) |
|  | 1* | 2 | 10 (6.94) | 64 (44.44) | 70 (48.61) | 0 (0) | 144 | 6.38 (1.87) | 6 (5 to 8); (1, 9) |
|  | 2 | 1 | 7 (4.67) | 78 (52) | 65 (43.33) | 0 (0) | 150 | 6.32 (1.57) | 6 (6 to 7); (1, 9) |
|  | 2* | 7 | 20 (9.85) | 100 (49.26) | 82 (40.39) | 1 (0.49) | 202 | 6.05 (1.83) | 6 (5 to 7); (1, 9) |
| [56.57/M] Any decision-making group or safety review committee, alternatively, an explanation of why such a committee is not needed | | | | | | | | | |
| SPIRIT | 1 | 12 | 5 (2.58) | 40 (20.62) | 147 (75.77) | 2 (1.03) | 192 | 7.48 (1.61) | 8 (7 to 9); (1, 9) |
|  | 1* | 2 | 4 (2.78) | 24 (16.67) | 115 (79.86) | 1 (0.69) | 143 | 7.55 (1.59) | 8 (7 to 9); (1, 9) |
|  | 2 | 2 | 2 (1.34) | 14 (9.4) | 133 (89.26) | 0 (0) | 149 | 8.01 (1.38) | 9 (7 to 9); (1, 9) |
|  | 2* | 10 | 3 (1.5) | 30 (15) | 166 (83) | 1 (0.5) | 199 | 7.84 (1.48) | 9 (7 to 9); (1, 9) |
| CONSORT | 1 | 12 | 10 (5.15) | 47 (24.23) | 136 (70.1) | 1 (0.52) | 193 | 7.05 (1.74) | 7 (6 to 9); (1, 9) |
|  | 1* | 2 | 8 (5.56) | 29 (20.14) | 106 (73.61) | 1 (0.69) | 143 | 7.11 (1.74) | 7 (6 to 9); (1, 9) |
|  | 2 | 2 | 3 (2.01) | 18 (12.08) | 128 (85.91) | 0 (0) | 149 | 7.43 (1.5) | 7 (7 to 9); (1, 9) |
|  | 2* | 10 | 5 (2.5) | 36 (18) | 159 (79.5) | 0 (0) | 200 | 7.3 (1.58) | 7 (7 to 9); (1, 9) |
| [58.59/M] Description of the plans for any interim data review (including data to be used for decision-making) and interim statistical analyses (e.g., safety/toxicity, dose [de-]escalation decisions) and stopping guidelines | | | | | | | | | |
| SPIRIT | 1 | 12 | 3 (1.55) | 30 (15.46) | 161 (82.99) | 0 (0) | 194 | 7.65 (1.43) | 8 (7 to 9); (1, 9) |
|  | 1* | 2 | 2 (1.39) | 20 (13.89) | 122 (84.72) | 0 (0) | 144 | 7.7 (1.43) | 8 (7 to 9); (1, 9) |
|  | 2 | 2 | 2 (1.34) | 8 (5.37) | 139 (93.29) | 0 (0) | 149 | 8.13 (1.31) | 9 (8 to 9); (1, 9) |
|  | 2* | 10 | 3 (1.5) | 19 (9.5) | 178 (89) | 0 (0) | 200 | 7.97 (1.37) | 8 (7 to 9); (1, 9) |
| CONSORT | 1 | 12 | 7 (3.61) | 44 (22.68) | 143 (73.71) | 0 (0) | 194 | 7.23 (1.64) | 7 (6 to 9); (1, 9) |
|  | 1* | 2 | 5 (3.47) | 28 (19.44) | 111 (77.08) | 0 (0) | 144 | 7.35 (1.61) | 7 (7 to 9); (1, 9) |
|  | 2 | 2 | 3 (2.01) | 10 (6.71) | 136 (91.28) | 0 (0) | 149 | 7.77 (1.41) | 8 (7 to 9); (1, 9) |
|  | 2* | 10 | 5 (2.5) | 27 (13.5) | 168 (84) | 0 (0) | 200 | 7.54 (1.53) | 8 (7 to 9); (1, 9) |
| [60/M] For each group, the number of participants who were assigned to each dose level at each interim analysis (e.g., for dosing decisions), received intended treatment, and were analysed for the primary outcome and, if applicable, any other outcomes used to inform pre-planned adaptations | | | | | | | | | |
| CONSORT | 1 | 12 | 4 (2.06) | 26 (13.4) | 163 (84.02) | 1 (0.52) | 193 | 7.7 (1.52) | 8 (7 to 9); (1, 9) |
|  | 1* | 2 | 2 (1.39) | 19 (13.19) | 123 (85.42) | 0 (0) | 144 | 7.81 (1.46) | 8 (7 to 9); (1, 9) |
|  | 2 | 2 | 2 (1.34) | 6 (4.03) | 141 (94.63) | 0 (0) | 149 | 8.24 (1.25) | 9 (8 to 9); (1, 9) |
|  | 2* | 10 | 4 (2) | 12 (6) | 183 (91.5) | 1 (0.5) | 199 | 8.04 (1.4) | 9 (7 to 9); (1, 9) |
| [61/N] Trial adaptation decisions made in light of the pre-planned decision-making criteria and observed accrued data | | | | | | | | | |
| CONSORT | 1 | 12 | 2 (1.03) | 34 (17.53) | 158 (81.44) | 0 (0) | 194 | 7.64 (1.36) | 8 (7 to 9); (3, 9) |
|  | 1* | 2 | 1 (0.69) | 22 (15.28) | 121 (84.03) | 0 (0) | 144 | 7.69 (1.33) | 8 (7 to 9); (3, 9) |
|  | 2 | 2 | 1 (0.67) | 13 (8.72) | 135 (90.6) | 0 (0) | 149 | 8.11 (1.21) | 9 (7 to 9); (3, 9) |
|  | 2* | 10 | 2 (1) | 24 (12) | 174 (87) | 0 (0) | 200 | 7.97 (1.28) | 8 (7 to 9); (3, 9) |
| [62/M*] Baseline demographic and clinical characteristics across each dose level within each group, where appropriate | | | | | | | | | |
| CONSORT | 1 | 12 | 3 (1.55) | 44 (22.68) | 144 (74.23) | 3 (1.55) | 191 | 7.39 (1.52) | 8 (7 to 9); (3, 9) |
|  | 1* | 2 | 2 (1.39) | 32 (22.22) | 108 (75) | 2 (1.39) | 142 | 7.41 (1.48) | 8 (7 to 9); (3, 9) |
|  | 2 | 2 | 2 (1.34) | 14 (9.4) | 132 (88.59) | 1 (0.67) | 148 | 8.02 (1.34) | 9 (7 to 9); (2, 9) |
|  | 2* | 10 | 3 (1.5) | 26 (13) | 169 (84.5) | 2 (1) | 198 | 7.85 (1.45) | 8 (7 to 9); (2, 9) |
| [63/M] For each group, the number of participants (denominator) included in each (interim/final) analysis across each dose level, and whether the analysis was by original assigned interventions | | | | | | | | | |
| CONSORT | 1 | 12 | 0 (0) | 32 (16.49) | 161 (82.99) | 1 (0.52) | 193 | 7.82 (1.34) | 8 (7 to 9); (4, 9) |
|  | 1* | 2 | 0 (0) | 22 (15.28) | 122 (84.72) | 0 (0) | 144 | 7.87 (1.28) | 8 (7 to 9); (4, 9) |
|  | 2 | 2 | 0 (0) | 7 (4.7) | 141 (94.63) | 1 (0.67) | 148 | 8.45 (0.9) | 9 (8 to 9); (5, 9) |
|  | 2* | 10 | 0 (0) | 17 (8.5) | 181 (90.5) | 2 (1) | 198 | 8.25 (1.13) | 9 (8 to 9); (4, 9) |
| [64/M] For each primary and secondary outcome, results for each dose level within each group, and the estimated effect size and its precision if applicable | | | | | | | | | |
| CONSORT | 1 | 12 | 11 (5.67) | 27 (13.92) | 155 (79.9) | 1 (0.52) | 193 | 7.47 (1.74) | 8 (7 to 9); (1, 9) |
|  | 1* | 2 | 5 (3.47) | 17 (11.81) | 122 (84.72) | 0 (0) | 144 | 7.69 (1.46) | 8 (7 to 9); (3, 9) |
|  | 2 | 2 | 1 (0.67) | 5 (3.36) | 143 (95.97) | 0 (0) | 149 | 8.32 (1.01) | 9 (8 to 9); (3, 9) |
|  | 2* | 10 | 7 (3.5) | 15 (7.5) | 177 (88.5) | 1 (0.5) | 199 | 7.93 (1.56) | 9 (7 to 9); (1, 9) |
| [65/N] Report interim results used to inform interim decision-making such as dose escalation, de-escalation or staying at the same dose | | | | | | | | | |
| CONSORT | 1 | 12 | 9 (4.64) | 35 (18.04) | 149 (76.8) | 1 (0.52) | 193 | 7.24 (1.69) | 8 (7 to 9); (2, 9) |
|  | 1* | 2 | 6 (4.17) | 24 (16.67) | 114 (79.17) | 0 (0) | 144 | 7.37 (1.63) | 8 (7 to 9); (2, 9) |
|  | 2 | 2 | 2 (1.34) | 10 (6.71) | 137 (91.95) | 0 (0) | 149 | 7.83 (1.29) | 8 (7 to 9); (2, 9) |
|  | 2* | 10 | 5 (2.5) | 21 (10.5) | 173 (86.5) | 1 (0.5) | 199 | 7.6 (1.49) | 8 (7 to 9); (2, 9) |
| [66/M] Plans for collecting, assessing, reporting, and managing solicited and spontaneously reported adverse events and other unintended effects of trial interventions (e.g., prior to any planned next dosing) or trial conduct | | | | | | | | | |
| SPIRIT | 1 | 12 | 3 (1.55) | 21 (10.82) | 169 (87.11) | 1 (0.52) | 193 | 7.96 (1.37) | 8 (7 to 9); (1, 9) |
|  | 1* | 2 | 3 (2.08) | 12 (8.33) | 128 (88.89) | 1 (0.69) | 143 | 8.03 (1.38) | 9 (7 to 9); (1, 9) |
|  | 2 | 2 | 1 (0.67) | 1 (0.67) | 147 (98.66) | 0 (0) | 149 | 8.58 (0.96) | 9 (8 to 9); (1, 9) |
|  | 2* | 10 | 1 (0.5) | 10 (5) | 189 (94.5) | 0 (0) | 200 | 8.38 (1.12) | 9 (8 to 9); (1, 9) |
| [67/M] All important toxicities and adverse events reported by dose level in each group (for specific guidance see CONSORT for harms) | | | | | | | | | |
| CONSORT | 1 | 12 | 2 (1.03) | 12 (6.19) | 179 (92.27) | 1 (0.52) | 193 | 8.13 (1.22) | 9 (8 to 9); (1, 9) |
|  | 1* | 2 | 1 (0.69) | 7 (4.86) | 135 (93.75) | 1 (0.69) | 143 | 8.22 (1.15) | 9 (8 to 9); (1, 9) |
|  | 2 | 2 | 1 (0.67) | 1 (0.67) | 147 (98.66) | 0 (0) | 149 | 8.64 (0.91) | 9 (9 to 9); (1, 9) |
|  | 2* | 10 | 2 (1) | 6 (3) | 192 (96) | 0 (0) | 200 | 8.46 (1.09) | 9 (8 to 9); (1, 9) |
| [68.69/M*] Specify if and when results (e.g., safety/response outcomes) can be shared externally/were reported whilst the trial is still ongoing | | | | | | | | | |
| SPIRIT | 1 | 12 | 8 (4.12) | 64 (32.99) | 116 (59.79) | 6 (3.09) | 188 | 6.91 (1.71) | 7 (6 to 8); (2, 9) |
|  | 1* | 2 | 8 (5.56) | 43 (29.86) | 88 (61.11) | 5 (3.47) | 139 | 6.88 (1.78) | 7 (6 to 8); (2, 9) |
|  | 2 | 2 | 2 (1.34) | 37 (24.83) | 109 (73.15) | 1 (0.67) | 148 | 7.3 (1.47) | 7 (6 to 9); (3, 9) |
|  | 2* | 10 | 3 (1.5) | 58 (29) | 137 (68.5) | 2 (1) | 198 | 7.19 (1.5) | 7 (6 to 9); (3, 9) |
| CONSORT | 1 | 12 | 14 (7.22) | 66 (34.02) | 108 (55.67) | 6 (3.09) | 188 | 6.59 (1.87) | 7 (6 to 8); (1, 9) |
|  | 1* | 2 | 9 (6.25) | 47 (32.64) | 83 (57.64) | 5 (3.47) | 139 | 6.63 (1.87) | 7 (6 to 8); (1, 9) |
|  | 2 | 2 | 4 (2.68) | 42 (28.19) | 102 (68.46) | 1 (0.67) | 148 | 6.84 (1.5) | 7 (6 to 8); (1, 9) |
|  | 2* | 10 | 9 (4.5) | 62 (31) | 127 (63.5) | 2 (1) | 198 | 6.72 (1.61) | 7 (6 to 8); (1, 9) |
| [70/M] Where the full trial protocol or the redacted version, with amendments (if any), can be accessed | | | | | | | | | |
| CONSORT | 1 | 12 | 5 (2.58) | 78 (40.21) | 110 (56.7) | 1 (0.52) | 193 | 6.83 (1.74) | 7 (6 to 8); (1, 9) |
|  | 1* | 2 | 3 (2.08) | 52 (36.11) | 89 (61.81) | 0 (0) | 144 | 6.99 (1.75) | 7 (6 to 9); (1, 9) |
|  | 2 | 1 | 2 (1.33) | 58 (38.67) | 90 (60) | 0 (0) | 150 | 7.14 (1.7) | 7 (6 to 9); (1, 9) |
|  | 2* | 10 | 4 (2) | 85 (42.5) | 110 (55) | 1 (0.5) | 199 | 6.93 (1.71) | 7 (6 to 9); (1, 9) |
| [71/N] Where other relevant trial documents (Oversight Committee, Safety Review Charter, quality aspects of investigational medicinal product, investigators brochure, simulation report, this list is non-exhaustive) can be accessed | | | | | | | | | |
| SPIRIT | 1 | 12 | 12 (6.19) | 91 (46.91) | 89 (45.88) | 2 (1.03) | 192 | 6.31 (1.76) | 6 (5 to 7.25); (1, 9) |
|  | 1* | 2 | 8 (5.56) | 67 (46.53) | 69 (47.92) | 0 (0) | 144 | 6.38 (1.74) | 6 (5 to 8); (1, 9) |
|  | 2 | 1 | 4 (2.67) | 77 (51.33) | 69 (46) | 0 (0) | 150 | 6.43 (1.44) | 6 (6 to 7); (1, 9) |
|  | 2* | 10 | 8 (4) | 101 (50.5) | 89 (44.5) | 2 (1) | 198 | 6.34 (1.54) | 6 (5 to 7); (1, 9) |
| [72/N] Where the full statistical analysis plan and other relevant trial documents (Oversight Committee, Safety Review/Data Monitoring Committee Charter, quality aspects of investigational medicinal product, investigators brochure, simulation report, this list is non-exhaustive) can be accessed | | | | | | | | | |
| CONSORT | 1 | 12 | 10 (5.15) | 89 (45.88) | 94 (48.45) | 1 (0.52) | 193 | 6.47 (1.71) | 6 (5 to 8); (1, 9) |
|  | 1* | 2 | 7 (4.86) | 62 (43.06) | 75 (52.08) | 0 (0) | 144 | 6.56 (1.71) | 7 (5 to 8); (1, 9) |
|  | 2 | 1 | 2 (1.33) | 80 (53.33) | 68 (45.33) | 0 (0) | 150 | 6.53 (1.44) | 6 (6 to 7); (1, 9) |
|  | 2* | 10 | 5 (2.5) | 107 (53.5) | 87 (43.5) | 1 (0.5) | 199 | 6.44 (1.5) | 6 (6 to 7); (1, 9) |
| [73/N*] Dose transition pathways or dose decision paths (using, for example, a flow diagram or table) projecting in advance how a proposed dose-finding design will recommend doses based on participants' key outcomes (e.g., what the next dose would be if x out of y participants experience significant adverse events) | | | | | | | | | |
| SPIRIT | 1 | 12 | 15 (7.73) | 78 (40.21) | 94 (48.45) | 7 (3.61) | 187 | 6.42 (1.73) | 7 (5 to 8); (3, 9) |
|  | 1* | 2 | 13 (9.03) | 49 (34.03) | 77 (53.47) | 5 (3.47) | 139 | 6.51 (1.75) | 7 (5.5 to 8); (3, 9) |
|  | 2 | 1 | 8 (5.33) | 70 (46.67) | 67 (44.67) | 5 (3.33) | 145 | 6.46 (1.42) | 6 (6 to 7); (3, 9) |
|  | 2* | 10 | 10 (5) | 98 (49) | 85 (42.5) | 7 (3.5) | 193 | 6.39 (1.48) | 6 (6 to 7); (3, 9) |
| [74.75/N*] Involvement of patients, service users, their carers, members of public or patient advocates in any aspect of the trial. Or reason why their involvement is/was not necessary | | | | | | | | | |
| SPIRIT | 1 | 12 | 23 (11.86) | 79 (40.72) | 77 (39.69) | 15 (7.73) | 179 | 6.06 (1.99) | 6 (5 to 7.5); (1, 9) |
|  | 1* | 2 | 15 (10.42) | 61 (42.36) | 61 (42.36) | 7 (4.86) | 137 | 6.14 (1.95) | 6 (5 to 7); (1, 9) |
|  | 2 | 1 | 12 (8) | 79 (52.67) | 56 (37.33) | 3 (2) | 147 | 6.2 (1.68) | 6 (6 to 7); (1, 9) |
|  | 2* | 10 | 20 (10) | 96 (48) | 73 (36.5) | 11 (5.5) | 189 | 6.14 (1.8) | 6 (5 to 7); (1, 9) |
| CONSORT | 1 | 12 | 25 (12.89) | 80 (41.24) | 72 (37.11) | 17 (8.76) | 177 | 5.95 (2) | 6 (5 to 7); (1, 9) |
|  | 1* | 2 | 17 (11.81) | 60 (41.67) | 59 (40.97) | 8 (5.56) | 136 | 6.04 (1.97) | 6 (5 to 7); (1, 9) |
|  | 2 | 1 | 15 (10) | 85 (56.67) | 47 (31.33) | 3 (2) | 147 | 6.07 (1.73) | 6 (6 to 7); (1, 9) |
|  | 2* | 10 | 23 (11.5) | 104 (52) | 61 (30.5) | 12 (6) | 188 | 6 (1.84) | 6 (5 to 7); (1, 9) |
| [76.77/N*] Lay summary of the trial synopsis/results or where it can be accessed | | | | | | | | | |
| SPIRIT | 1 | 12 | 18 (9.28) | 93 (47.94) | 78 (40.21) | 5 (2.58) | 189 | 6.05 (1.98) | 6 (5 to 8); (1, 9) |
|  | 1* | 2 | 13 (9.03) | 66 (45.83) | 63 (43.75) | 2 (1.39) | 142 | 6.15 (1.92) | 6 (5 to 8); (1, 9) |
|  | 2 | 1 | 7 (4.67) | 86 (57.33) | 55 (36.67) | 2 (1.33) | 148 | 6.28 (1.67) | 6 (5 to 7); (1, 9) |
|  | 2* | 10 | 12 (6) | 112 (56) | 71 (35.5) | 5 (2.5) | 195 | 6.17 (1.82) | 6 (5 to 7.5); (1, 9) |
| CONSORT | 1 | 12 | 20 (10.31) | 89 (45.88) | 81 (41.75) | 4 (2.06) | 190 | 6.03 (1.99) | 6 (4.25 to 8); (1, 9) |
|  | 1* | 2 | 16 (11.11) | 63 (43.75) | 64 (44.44) | 1 (0.69) | 143 | 6.08 (1.94) | 6 (5 to 8); (1, 9) |
|  | 2 | 1 | 9 (6) | 79 (52.67) | 61 (40.67) | 1 (0.67) | 149 | 6.26 (1.69) | 6 (5 to 7); (1, 9) |
|  | 2* | 10 | 13 (6.5) | 104 (52) | 79 (39.5) | 4 (2) | 196 | 6.19 (1.81) | 6 (5 to 8); (1, 9) |
| [78/M] Identification as a dose-finding trial | | | | | | | | | |
| CONSORT | 1 | 12 | 4 (2.06) | 50 (25.77) | 137 (70.62) | 3 (1.55) | 191 | 7.34 (1.58) | 8 (6 to 9); (1, 9) |
|  | 1* | 2 | 1 (0.69) | 35 (24.31) | 108 (75) | 0 (0) | 144 | 7.49 (1.43) | 8 (6.75 to 9); (3, 9) |
|  | 2 | 1 | 0 (0) | 28 (18.67) | 122 (81.33) | 0 (0) | 150 | 7.88 (1.29) | 9 (7 to 9); (4, 9) |
|  | 2* | 10 | 3 (1.5) | 43 (21.5) | 151 (75.5) | 3 (1.5) | 197 | 7.63 (1.52) | 8 (7 to 9); (1, 9) |
| [79/N] Dose decisions/adaptations were made in light of pre-planned decision-making criteria and observed accrued data | | | | | | | | | |
| CONSORT | 1 | 12 | 10 (5.15) | 67 (34.54) | 113 (58.25) | 4 (2.06) | 190 | 6.78 (1.76) | 7 (6 to 8); (1, 9) |
|  | 1* | 2 | 6 (4.17) | 51 (35.42) | 86 (59.72) | 1 (0.69) | 143 | 6.83 (1.67) | 7 (6 to 8); (2, 9) |
|  | 2 | 1 | 4 (2.67) | 43 (28.67) | 103 (68.67) | 0 (0) | 150 | 7.13 (1.49) | 7 (6 to 9); (2, 9) |
|  | 2* | 10 | 8 (4) | 59 (29.5) | 130 (65) | 3 (1.5) | 197 | 7 (1.63) | 7 (6 to 9); (1, 9) |
| [80/ADD] Access (or link to) to code/functions used for simulation studies | | | | | | | | | |
| CONSORT | 2 | 1 | 9 (6) | 87 (58) | 46 (30.67) | 8 (5.33) | 142 | 5.96 (1.62) | 6 (5 to 7); (1, 9) |

*
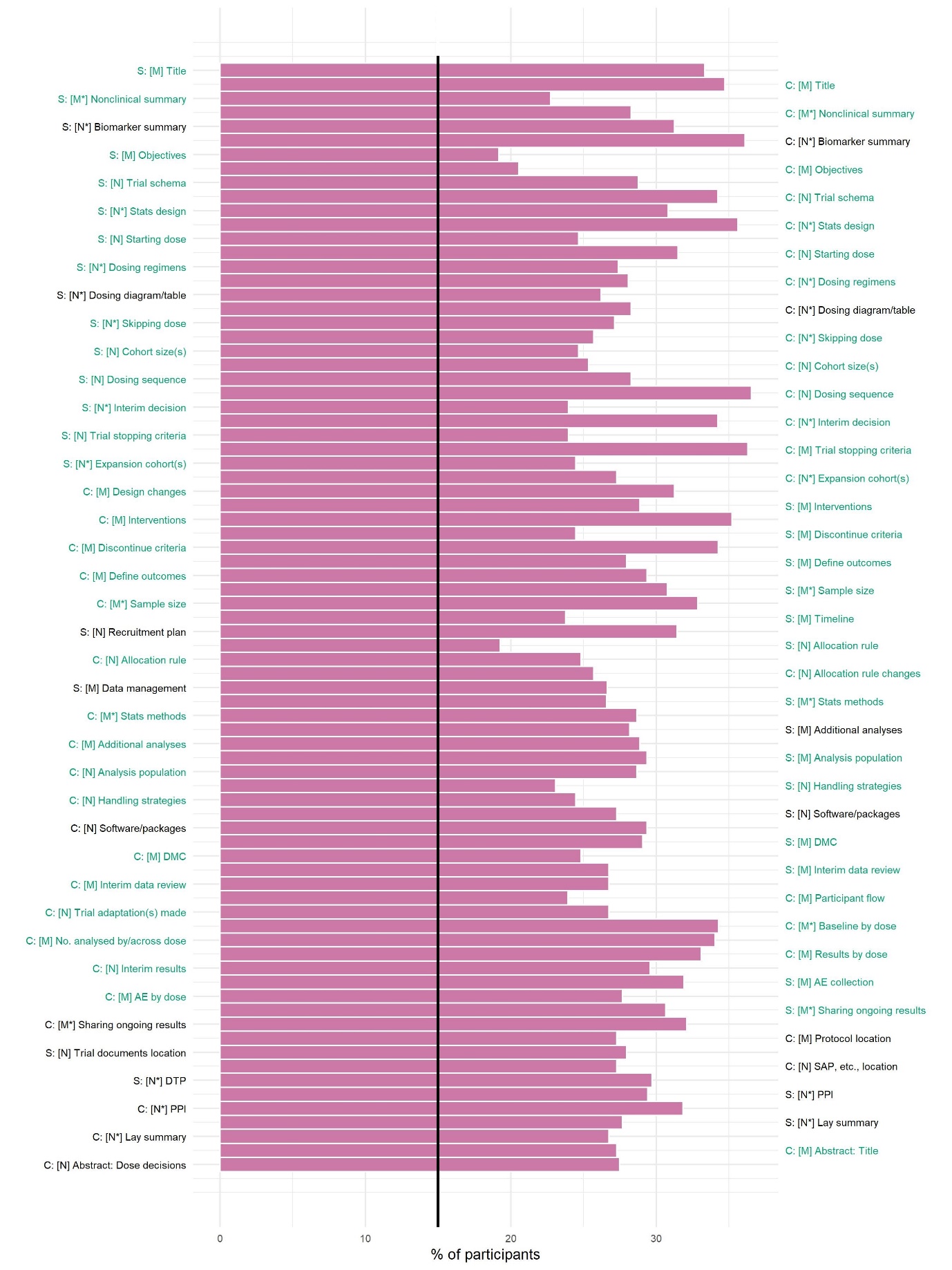
Figure A9-1. Percentage of participants changing their numerical scores at Round Two. Items with asterisk (*) are items with wordings modified based on comments from Round One.*

*
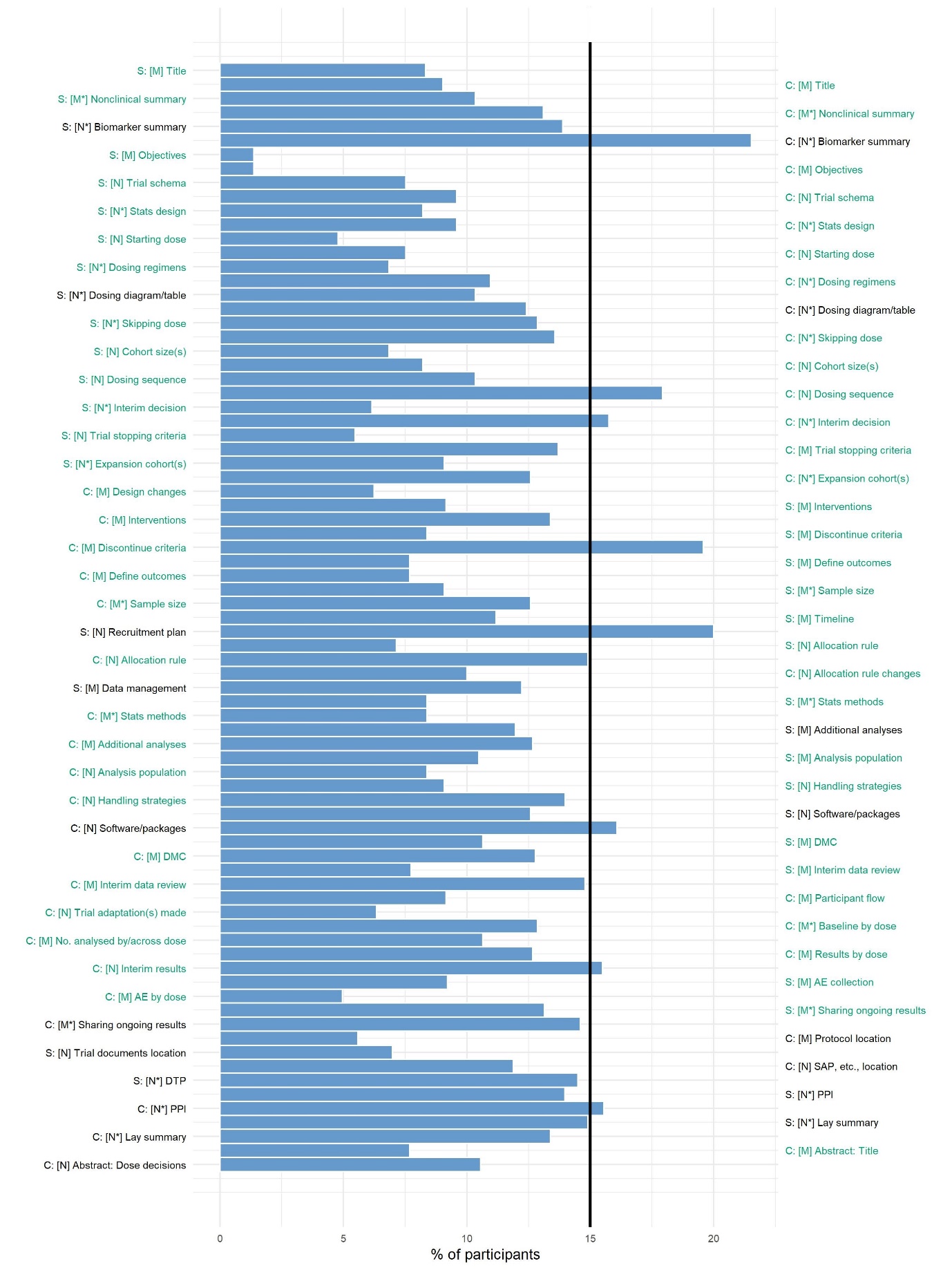
*

*Figure A9-2. Percentage of participants changing their categorical scores at Round Two. Items with asterisk (*) are items with wordings modified based on comments from Round One.*

*Table A9-2. Number of participants changing their ratings between Round One and Round Two.*

| Item | (Unable to rate) to Important but not critical) | To higher rating | | | To lower rating | |
| --- | --- | --- | --- | --- | --- | --- |
|  |  | (Not important) to (Important but not critical) | (Not important) to (Critical) | (Important but not critical) to (Critical) | (Critical) to (Important but not critical) | (Important but not critical) to (Not important) |
| [01/M] SPIRIT: Identification as a first-in-human or early phase dose-finding trial | 0 | 0 | 0 | 2 | 1 | 0 |
| [02/M] CONSORT: Identification as a first-in-human or early phase dose-finding trial | 0 | 0 | 0 | 3 | 1 | 0 |
| [03/M*] SPIRIT: Summary of key findings from relevant nonclinical/pre-clinical research | 0 | 1 | 0 | 5 | 0 | 0 |
| [04/M*] CONSORT: Summary of key findings from relevant nonclinical/pre-clinical research | 0 | 1 | 0 | 8 | 0 | 0 |
| [05/N*] SPIRIT: Summary of findings from existing correlative biomarker, correlative and associated studies to support planned biomarker sub-study (if applicable) | 0 | 1 | 0 | 2 | 6 | 0 |
| [06/N*] CONSORT: Summary of findings from existing correlative biomarker, correlative and associated studies to support planned biomarker sub-study (if applicable) | 0 | 4 | 0 | 1 | 9 | 0 |
| [08/M] CONSORT: Specific objectives (e.g., safety, activity, pharmacokinetics, pharmacodynamics) or hypotheses | 0 | 0 | 0 | 1 | 0 | 0 |
| [09/N] SPIRIT: Trial design schema (to show flow of decision points, e.g., dose escalation to expansion) | 0 | 0 | 0 | 5 | 0 | 0 |
| [10/N] CONSORT: Trial design schema (to show flow of decision points, e.g., dose escalation to expansion) | 0 | 0 | 0 | 4 | 0 | 0 |
| [11/N*] SPIRIT: Statistical methodology or rationale underpinning the trial design | 0 | 0 | 0 | 3 | 0 | 0 |
| [12/N*] CONSORT: Statistical methodology or rationale underpinning the trial design | 0 | 0 | 0 | 4 | 0 | 0 |
| [13/N] SPIRIT: Starting dose(s) specification with rationale | 0 | 0 | 0 | 3 | 0 | 0 |
| [14/N] CONSORT: Starting dose(s) specification with rationale | 0 | 0 | 0 | 4 | 0 | 0 |
| [15/N*] SPIRIT: Dosing regimens (e.g., doses/schedules or intensity of fractionation) considered with rationale | 0 | 0 | 0 | 5 | 1 | 0 |
| [16/N*] CONSORT: Dosing regimens (e.g., doses/schedules or intensity of fractionation) considered with rationale | 0 | 0 | 0 | 5 | 1 | 0 |
| [17/N*] SPIRIT: Planned dosing regimens presented as a diagram or table, where applicable | 0 | 1 | 0 | 3 | 0 | 0 |
| [18/N*] CONSORT: Planned and delivered dosing regimens presented as a diagram or table, where applicable | 0 | 1 | 0 | 4 | 0 | 0 |
| [19/N*] SPIRIT: Specify whether skipping of dose level is permissible, if applicable | 0 | 1 | 0 | 7 | 0 | 0 |
| [20/N*] CONSORT: Specify whether skipping of dose level is permissible, if applicable | 0 | 0 | 1 | 7 | 0 | 0 |
| [21/N] SPIRIT: Planned cohort size(s) (fixed or flexible) | 0 | 0 | 0 | 1 | 0 | 0 |
| [22/N] CONSORT: Planned cohort size(s) (fixed or flexible) | 0 | 0 | 0 | 2 | 0 | 0 |
| [23/N] SPIRIT: Sequence and interval between dosing of participants | 0 | 0 | 0 | 6 | 0 | 0 |
| [24/N] CONSORT: Sequence and interval between dosing of participants | 0 | 0 | 0 | 10 | 0 | 0 |
| [25/N*] SPIRIT: Pre-planned interim decision-making criteria/rules to guide the trial adaptation process (e.g., dosing decision to [de-]escalate), pre-planned timing and frequency of interim data looks and the information to inform the adaptations. Alternatively, an explanation of why they are not pre-specified | 0 | 0 | 0 | 1 | 0 | 0 |
| [26/N*] CONSORT: Pre-planned interim decision-making criteria/rules to guide the trial adaptation process (e.g., dosing decision to [de-]escalate), pre-planned timing and frequency of interim data looks and the information to inform the adaptations. Alternatively, an explanation of why they are not pre-specified | 0 | 0 | 0 | 3 | 0 | 0 |
| [27/N] SPIRIT: Trial stopping criteria and consequences | 0 | 0 | 0 | 0 | 1 | 0 |
| [28/M] CONSORT: Trial stopping criteria and consequences | 0 | 0 | 0 | 1 | 1 | 0 |
| [29/N*] SPIRIT: Dose expansion cohort(s), if applicable | 0 | 0 | 0 | 3 | 0 | 0 |
| [30/N*] CONSORT: Dose expansion cohort(s), if applicable | 0 | 0 | 0 | 5 | 0 | 0 |
| [31/M] CONSORT: Important changes to the design or methods after trial commencement that are relevant for dose determination outside the scope of the pre-planned adaptive design features, with reasons | 0 | 0 | 0 | 1 | 0 | 0 |
| [32/M] SPIRIT: The interventions for each dose level (and within each treatment group for randomised early phase trials, if applicable) | 0 | 0 | 0 | 3 | 0 | 0 |
| [33/M] CONSORT: The interventions for each dose level (and within each treatment group for randomised early phase trials, if applicable) | 0 | 0 | 0 | 2 | 0 | 0 |
| [34/M] SPIRIT: Criteria for discontinuing, dose modifications and dosing delays of allocated interventions for a given trial participant | 0 | 0 | 0 | 2 | 0 | 0 |
| [35/M] CONSORT: Criteria for discontinuing, dose modifications and dosing delays of allocated interventions for a given trial participant | 0 | 0 | 0 | 4 | 1 | 1 |
| [36/M] SPIRIT: Define pre-specified primary and secondary outcome measures, including how and when they were assessed. Any other outcome measures used to inform pre-planned adaptations should be described with the rationale | 0 | 0 | 0 | 1 | 0 | 0 |
| [37/M] CONSORT: Define pre-specified primary and secondary outcome measures, including how and when they were assessed. Any other outcome measures used to inform pre-planned adaptations should be described with the rationale | 0 | 0 | 0 | 1 | 0 | 0 |
| [38/M*] SPIRIT: Estimated number of participants (minimum/lower bound, maximum or expected range) needed to address study objectives and how it was determined, including clinical and statistical assumptions supporting any sample size AND operating characteristics | 0 | 0 | 0 | 5 | 0 | 0 |
| [39/M*] CONSORT: Estimated number of participants (minimum/lower bound, maximum or expected range) needed to address study objectives and how it was determined, including clinical and statistical assumptions supporting any sample size AND operating characteristics | 0 | 0 | 0 | 3 | 0 | 0 |
| [40/M] SPIRIT: Time schedule of enrolment, interventions (including any run-ins and washouts), assessments, and visits for participants (including in-house stay or out-patient follow-up period where applicable). A schematic diagram is highly recommended | 0 | 0 | 1 | 3 | 0 | 0 |
| [41/N] SPIRIT: Plans for recruitment/screening slots for sequential cohorts of participants | 0 | 1 | 0 | 3 | 6 | 0 |
| [42/N] SPIRIT: Any pre-planned allocation rule or algorithm to update randomisation with timing and frequency of updates | 0 | 0 | 0 | 3 | 0 | 0 |
| [43/N] CONSORT: Any pre-planned allocation rule or algorithm to update randomisation with timing and frequency of updates | 0 | 0 | 0 | 6 | 0 | 0 |
| [44/N] CONSORT: Any changes to the allocation rule after trial adaptation decisions | 0 | 0 | 0 | 5 | 0 | 0 |
| [45/M] SPIRIT: Specify if the data management plans in the initial dose-finding component are different from subsequent stages (e.g., expansion cohort(s) or Phase II) of the trial | 1 | 0 | 0 | 0 | 4 | 0 |
| [46/M*] SPIRIT: For the proposed adaptive design features, statistical methods used to estimate key endpoints (e.g., safety, dose(s), treatment effects) | 0 | 0 | 0 | 5 | 0 | 0 |
| [47/M*] CONSORT: For the proposed adaptive design features, statistical methods used to estimate key endpoints (e.g., safety, dose(s), treatment effects) | 0 | 0 | 0 | 2 | 0 | 0 |
| [48/M] SPIRIT: Statistical methods for additional analyses (e.g., subgroup and adjusted analyses, PK/PD, biomarker correlative analyses) | 0 | 0 | 0 | 4 | 0 | 0 |
| [49/M] CONSORT: Statistical methods for additional analyses (e.g., subgroup and adjusted analyses, PK/PD, biomarker correlative analyses) | 0 | 0 | 0 | 1 | 3 | 0 |
| [50/M] SPIRIT: Clearly-defined analysis population (e.g., evaluable population for dose determination, safety and key outcomes) | 0 | 0 | 0 | 6 | 0 | 0 |
| [51/N] CONSORT: Clearly-defined analysis population (e.g., evaluable population for dose determination, safety and key outcomes) | 0 | 0 | 0 | 2 | 0 | 0 |
| [52/N] SPIRIT: Pre-specify handling strategies of events occurring after treatment initiation (e.g., how dosing delays will be handled) that affect either the interpretation or the existence of the measurements associated with the clinical question of interest | 0 | 0 | 0 | 3 | 0 | 0 |
| [53/N] CONSORT: Pre-specify handling strategies of events occurring after treatment initiation (e.g., how dosing delays will be handled) that affect either the interpretation or the existence of the measurements associated with the clinical question of interest | 0 | 0 | 0 | 2 | 1 | 0 |
| [54/N] SPIRIT: Statistical software and packages used for design (e.g., simulation) and to be used for planned analyses | 0 | 1 | 0 | 1 | 5 | 1 |
| [55/N] CONSORT: Statistical software and packages used | 0 | 0 | 0 | 1 | 8 | 1 |
| [56/M] SPIRIT: Any decision-making group or safety review committee, alternatively, an explanation of why such a committee is not needed | 0 | 1 | 0 | 4 | 0 | 0 |
| [57/M] CONSORT: Any decision-making group or safety review committee | 0 | 1 | 0 | 6 | 0 | 0 |
| [58/M] SPIRIT: Description of the plans for any interim data review (including data to be used for decision-making) and interim statistical analyses (e.g., safety/toxicity, dose [de-]escalation decisions) and stopping guidelines | 0 | 0 | 0 | 1 | 0 | 0 |
| [59/M] CONSORT: Description of the plans for any interim data review (including data to be used for decision-making) and interim statistical analyses (e.g., safety/toxicity, dose [de-]escalation decisions) and stopping guidelines | 0 | 0 | 0 | 5 | 0 | 0 |
| [60/M] CONSORT: For each group, the number of participants who were assigned to each dose level at each interim analysis (e.g., for dosing decisions), received intended treatment, and were analysed for the primary outcome and, if applicable, any other outcomes used to inform pre-planned adaptations | 0 | 0 | 0 | 2 | 0 | 0 |
| [61/N] CONSORT: Trial adaptation decisions made in light of the pre-planned decision-making criteria and observed accrued data | 0 | 0 | 0 | 2 | 0 | 0 |
| [62/M*] CONSORT: Baseline demographic and clinical characteristics across each dose level within each group, where appropriate | 0 | 0 | 0 | 4 | 0 | 0 |
| [63/M] CONSORT: For each group, the number of participants (denominator) included in each (interim/final) analysis across each dose level, and whether the analysis was by original assigned interventions | 0 | 0 | 0 | 2 | 0 | 0 |
| [64/M] CONSORT: For each primary and secondary outcome, results for each dose level within each group, and the estimated effect size and its precision if applicable | 0 | 1 | 1 | 3 | 0 | 0 |
| [65/N] CONSORT: Report interim results used to inform interim decision-making such as dose escalation, de-escalation or staying at the same dose | 0 | 2 | 0 | 4 | 0 | 0 |
| [66/M] SPIRIT: Plans for collecting, assessing, reporting, and managing solicited and spontaneously reported adverse events and other unintended effects of trial interventions (e.g., prior to any planned next dosing) or trial conduct | 0 | 0 | 0 | 4 | 0 | 0 |
| [67/M] CONSORT: All important toxicities and adverse events reported by dose level in each group (for specific guidance see CONSORT for harms) | 0 | 0 | 0 | 3 | 0 | 0 |
| [68/M*] SPIRIT: Specify if and when results (e.g., safety/response outcomes) can be shared externally whilst the trial is still ongoing | 0 | 1 | 0 | 5 | 0 | 0 |
| [69/M*] CONSORT: Specify if and when results (e.g., safety/response outcomes) were reported whilst the trial was still ongoing | 0 | 1 | 0 | 5 | 0 | 0 |
| [70/M] CONSORT: Where the full trial protocol or the redacted version, with amendments (if any), can be accessed | 0 | 0 | 0 | 1 | 1 | 0 |
| [71/N] SPIRIT: Where other relevant trial documents (Oversight Committee, Safety Review Charter, quality aspects of investigational medicinal product, investigators brochure, simulation report, this list is non-exhaustive) can be accessed | 0 | 2 | 0 | 0 | 1 | 0 |
| [72/N] CONSORT: Where the full statistical analysis plan and other relevant trial documents (Oversight Committee, Safety Review/Data Monitoring Committee Charter, quality aspects of investigational medicinal product, investigators brochure, simulation report, this list is non-exhaustive) can be accessed | 0 | 2 | 0 | 1 | 1 | 0 |
| [73/N*] SPIRIT: Dose transition pathways or dose decision paths (using, for example, a flow diagram or table) projecting in advance how a proposed dose-finding design will recommend doses based on participants’ key outcomes (e.g., what the next dose would be if x out of y participants experience significant adverse events). | 0 | 1 | 0 | 1 | 1 | 0 |
| [74/N*] SPIRIT: Involvement of patients, service users, their carers, members of public or patient advocates in any aspect of the trial. Or reason why their involvement is not necessary | 2 | 0 | 0 | 2 | 6 | 0 |
| [75/N*] CONSORT: Involvement of patients, service users, their carers, members of public or patient advocates in any aspect of the trial. Or reason why their involvement was not necessary | 2 | 0 | 0 | 2 | 7 | 0 |
| [76/N*] SPIRIT: Lay summary of the trial synopsis or where it can be accessed | 0 | 0 | 1 | 0 | 8 | 1 |
| [77/N*] CONSORT: Lay summary of the trial results or where it can be accessed | 0 | 0 | 1 | 0 | 5 | 1 |
| [78/M] CONSORT-abstract: Identification as a dose-finding trial | 0 | 0 | 0 | 3 | 0 | 0 |
| [79/N] CONSORT-abstract: Dose decisions/adaptations were made in light of pre-planned decision-making criteria and observed accrued data | 0 | 0 | 0 | 3 | 0 | 0 |

*Table A9-3. Frequency (n/N) and percentage (%) of perfect agreement with its 95% confidence interval (CI), and weighted Cohen’s kappa coefficient (k) of agreement between rounds one and two and its 95% CI.*

| Guidance | Categorical rating | | Numerical rating | |
| --- | --- | --- | --- | --- |
|  | n/N; % (95% CI) | κ (95% CI) | n/N; % (95% CI) | κ (95% CI) |
| [1.2/M] Identification as a first-in-human or early phase dose-finding trial | | | |  |
| SPIRIT | 132/144; 91.67 (85.9 to 95.62) | 0.5 (0.25 to 0.72) | 96/144; 66.67 (58.34 to 74.3) | 0.55 (0.44 to 0.66) |
| CONSORT | 131/144; 90.97 (85.06 to 95.11) | 0.48 (0.24 to 0.7) | 94/144; 65.28 (56.9 to 73.01) | 0.52 (0.41 to 0.63) |
| [3.4/M*] Summary of key findings from relevant nonclinical/pre-clinical research | | | |  |
| SPIRIT | 130/145; 89.66 (83.51 to 94.09) | 0.66 (0.49 to 0.8) | 112/145; 77.24 (69.55 to 83.79) | 0.77 (0.68 to 0.84) |
| CONSORT | 126/145; 86.9 (80.3 to 91.92) | 0.72 (0.58 to 0.82) | 104/145; 71.72 (63.65 to 78.88) | 0.72 (0.63 to 0.8) |
| [5.6/N*] Summary of findings from existing correlative biomarker, correlative and associated studies to support planned biomarker sub-study (if applicable) | | | | |
| SPIRIT | 124/144; 86.11 (79.37 to 91.31) | 0.72 (0.61 to 0.82) | 99/144; 68.75 (60.5 to 76.21) | 0.7 (0.61 to 0.78) |
| CONSORT | 113/144; 78.47 (70.86 to 84.88) | 0.6 (0.46 to 0.71) | 92/144; 63.89 (55.47 to 71.72) | 0.62 (0.51 to 0.71) |
| [7.8/M] Specific objectives (e.g., safety, activity, pharmacokinetics, pharmacodynamics) or hypotheses | | | | |
| SPIRIT | 144/146; 98.63 (95.14 to 99.83) | 0.66 (0 to 1) | 118/146; 80.82 (73.49 to 86.86) | 0.55 (0.4 to 0.71) |
| CONSORT | 144/146; 98.63 (95.14 to 99.83) | 0.66 (0 to 1) | 116/146; 79.45 (71.98 to 85.69) | 0.54 (0.41 to 0.69) |
| [9.10/N] Trial design schema (to show flow of decision points, e.g., dose escalation to expansion) | | | | |
| SPIRIT | 135/146; 92.47 (86.92 to 96.18) | 0.49 (0.24 to 0.72) | 104/146; 71.23 (63.17 to 78.42) | 0.6 (0.48 to 0.71) |
| CONSORT | 132/146; 90.41 (84.43 to 94.66) | 0.55 (0.35 to 0.73) | 96/146; 65.75 (57.45 to 73.4) | 0.58 (0.48 to 0.68) |
| [11.12/N*] Statistical methodology or rationale underpinning the trial design | | | |  |
| SPIRIT | 134/146; 91.78 (86.08 to 95.68) | 0.47 (0.18 to 0.75) | 101/146; 69.18 (61.01 to 76.55) | 0.57 (0.46 to 0.69) |
| CONSORT | 132/146; 90.41 (84.43 to 94.66) | 0.57 (0.36 to 0.79) | 94/146; 64.38 (56.04 to 72.13) | 0.56 (0.45 to 0.66) |
| [13.14/N] Starting dose(s) specification with rationale | | |  |  |
| SPIRIT | 139/146; 95.21 (90.37 to 98.05) | 0.35 (0 to 0.73) | 110/146; 75.34 (67.53 to 82.09) | 0.63 (0.52 to 0.73) |
| CONSORT | 135/146; 92.47 (86.92 to 96.18) | 0.57 (0.33 to 0.8) | 100/146; 68.49 (60.29 to 75.92) | 0.61 (0.51 to 0.71) |
| [15.16/N*] Dosing regimens (e.g., doses/schedules or intensity of fractionation) considered with rationale | | | | |
| SPIRIT | 136/146; 93.15 (87.76 to 96.67) | 0.47 (0.17 to 0.75) | 106/146; 72.6 (64.61 to 79.65) | 0.66 (0.56 to 0.74) |
| CONSORT | 130/146; 89.04 (82.81 to 93.6) | 0.47 (0.24 to 0.71) | 105/146; 71.92 (63.89 to 79.03) | 0.65 (0.55 to 0.74) |
| [17.18/N*] Planned (and delivered) dosing regimens presented as a diagram or table, where applicable | | | | |
| SPIRIT | 130/145; 89.66 (83.51 to 94.09) | 0.79 (0.68 to 0.88) | 107/145; 73.79 (65.85 to 80.74) | 0.79 (0.71 to 0.85) |
| CONSORT | 127/145; 87.59 (81.09 to 92.47) | 0.78 (0.69 to 0.87) | 104/145; 71.72 (63.65 to 78.88) | 0.76 (0.67 to 0.83) |
| [19.20/N*] Specify whether skipping of dose level is permissible, if applicable | | | |  |
| SPIRIT | 122/140; 87.14 (80.44 to 92.2) | 0.59 (0.43 to 0.75) | 102/140; 72.86 (64.7 to 80.02) | 0.69 (0.6 to 0.79) |
| CONSORT | 121/140; 86.43 (79.62 to 91.63) | 0.68 (0.51 to 0.8) | 104/140; 74.29 (66.22 to 81.29) | 0.74 (0.63 to 0.82) |
| [21.22/N] Planned cohort size(s) (fixed or flexible) | | |  |  |
| SPIRIT | 136/146; 93.15 (87.76 to 96.67) | 0.66 (0.39 to 0.83) | 110/146; 75.34 (67.53 to 82.09) | 0.67 (0.56 to 0.77) |
| CONSORT | 134/146; 91.78 (86.08 to 95.68) | 0.66 (0.45 to 0.83) | 109/146; 74.66 (66.8 to 81.49) | 0.69 (0.58 to 0.79) |
| [23.24/N] Sequence and interval between dosing of participants | | | |  |
| SPIRIT | 130/145; 89.66 (83.51 to 94.09) | 0.6 (0.41 to 0.79) | 104/145; 71.72 (63.65 to 78.88) | 0.67 (0.57 to 0.76) |
| CONSORT | 119/145; 82.07 (74.84 to 87.94) | 0.57 (0.41 to 0.71) | 92/145; 63.45 (55.05 to 71.28) | 0.6 (0.5 to 0.71) |
| [25.26/N*] Pre-planned interim decision-making criteria/rules to guide the trial adaptation process (e.g., dosing decision to [de-]escalate), pre-planned timing and frequency of interim data looks and the information to inform the adaptations. Alternatively, an explanation of why they are not pre-specified | | | | |
| SPIRIT | 137/146; 93.84 (88.62 to 97.14) | 0.38 (0.07 to 0.74) | 111/146; 76.03 (68.27 to 82.7) | 0.62 (0.49 to 0.75) |
| CONSORT | 123/146; 84.25 (77.31 to 89.74) | 0.41 (0.22 to 0.6) | 96/146; 65.75 (57.45 to 73.4) | 0.57 (0.45 to 0.67) |
| [27.28/N] Trial stopping criteria and consequences | | |  |  |
| SPIRIT | 138/146; 94.52 (89.49 to 97.6) | 0.58 (0.2 to 0.82) | 111/146; 76.03 (68.27 to 82.7) | 0.63 (0.51 to 0.75) |
| CONSORT | 126/146; 86.3 (79.64 to 91.43) | 0.44 (0.26 to 0.67) | 93/146; 63.7 (55.34 to 71.49) | 0.57 (0.47 to 0.67) |
| [29.30/N*] Dose expansion cohort(s), if applicable | | |  |  |
| SPIRIT | 130/143; 90.91 (84.96 to 95.07) | 0.64 (0.45 to 0.8) | 108/143; 75.52 (67.64 to 82.32) | 0.7 (0.61 to 0.79) |
| CONSORT | 125/143; 87.41 (80.84 to 92.37) | 0.61 (0.44 to 0.75) | 104/143; 72.73 (64.65 to 79.83) | 0.68 (0.58 to 0.77) |
| [31/M] Important changes to the design or methods after trial commencement that are relevant for dose determination outside the scope of the pre-planned adaptive design features, with reasons | | | | |
| CONSORT | 135/144; 93.75 (88.47 to 97.1) | 0.45 (0.17 to 0.74) | 99/144; 68.75 (60.5 to 76.21) | 0.56 (0.45 to 0.68) |
| [32.33/M] The interventions for each dose level (and within each treatment group for randomised early phase trials, if applicable) | | | | |
| SPIRIT | 129/142; 90.85 (84.85 to 95.03) | 0.45 (0.23 to 0.68) | 101/142; 71.13 (62.93 to 78.42) | 0.66 (0.56 to 0.74) |
| CONSORT | 123/142; 86.62 (79.9 to 91.75) | 0.45 (0.26 to 0.65) | 92/142; 64.79 (56.34 to 72.61) | 0.6 (0.49 to 0.69) |
| [34.35/M] Criteria for discontinuing, dose modifications and dosing delays of allocated interventions for a given trial participant | | | | |
| SPIRIT | 131/143; 91.61 (85.8 to 95.59) | 0.51 (0.25 to 0.76) | 108/143; 75.52 (67.64 to 82.32) | 0.64 (0.54 to 0.75) |
| CONSORT | 115/143; 80.42 (72.96 to 86.58) | 0.44 (0.28 to 0.61) | 94/143; 65.73 (57.34 to 73.46) | 0.59 (0.49 to 0.69) |
| [36.37/M] Define pre-specified primary and secondary outcome measures, including how and when they were assessed. Any other outcome measures used to inform pre-planned adaptations should be described with the rationale | | | | |
| SPIRIT | 132/143; 92.31 (86.65 to 96.1) | 0.45 (0.13 to 0.78) | 103/143; 72.03 (63.91 to 79.21) | 0.52 (0.39 to 0.67) |
| CONSORT | 132/143; 92.31 (86.65 to 96.1) | 0.57 (0.26 to 0.8) | 101/143; 70.63 (62.44 to 77.94) | 0.56 (0.42 to 0.68) |
| [38.39/M*] Estimated number of participants (minimum/lower bound, maximum or expected range) needed to address study objectives and how it was determined, including clinical and statistical assumptions supporting any sample size AND operating characteristics | | | | |
| SPIRIT | 130/143; 90.91 (84.96 to 95.07) | 0.63 (0.42 to 0.79) | 99/143; 69.23 (60.97 to 76.67) | 0.64 (0.54 to 0.73) |
| CONSORT | 125/143; 87.41 (80.84 to 92.37) | 0.67 (0.54 to 0.8) | 96/143; 67.13 (58.79 to 74.75) | 0.65 (0.56 to 0.74) |
| [40/M] Time schedule of enrolment, interventions (including any run-ins and washouts), assessments, and visits for participants (including in-house stay or out-patient follow-up period where applicable). A schematic diagram is highly recommended | | | | |
| SPIRIT | 127/143; 88.81 (82.47 to 93.47) | 0.64 (0.46 to 0.78) | 109/143; 76.22 (68.39 to 82.94) | 0.72 (0.6 to 0.81) |
| [41/N] Plans for recruitment/screening slots for sequential cohorts of participants | | | |  |
| SPIRIT | 112/140; 80 (72.41 to 86.28) | 0.67 (0.54 to 0.77) | 96/140; 68.57 (60.19 to 76.15) | 0.7 (0.6 to 0.78) |
| [42.43/N] Any pre-planned allocation rule or algorithm to update randomisation with timing and frequency of updates | | | | |
| SPIRIT | 130/140; 92.86 (87.26 to 96.52) | 0.76 (0.59 to 0.88) | 113/140; 80.71 (73.19 to 86.89) | 0.79 (0.71 to 0.87) |
| CONSORT | 120/141; 85.11 (78.14 to 90.54) | 0.64 (0.48 to 0.78) | 106/141; 75.18 (67.21 to 82.06) | 0.74 (0.63 to 0.82) |
| [44/N] Any changes to the allocation rule after trial adaptation decisions | | | |  |
| CONSORT | 126/140; 90 (83.79 to 94.42) | 0.68 (0.51 to 0.82) | 104/140; 74.29 (66.22 to 81.29) | 0.72 (0.63 to 0.8) |
| [45/M] Specify if the data management plans in the initial dose-finding component are different from subsequent stages (e.g., expansion cohort(s) or Phase II) of the trial | | | | |
| SPIRIT | 122/139; 87.77 (81.14 to 92.71) | 0.8 (0.7 to 0.88) | 102/139; 73.38 (65.22 to 80.51) | 0.78 (0.69 to 0.84) |
| [46.47/M*] For the proposed adaptive design features, statistical methods used to estimate key endpoints (e.g., safety, dose(s), treatment effects) | | | | |
| SPIRIT | 131/143; 91.61 (85.8 to 95.59) | 0.47 (0.23 to 0.71) | 105/143; 73.43 (65.4 to 80.46) | 0.63 (0.52 to 0.72) |
| CONSORT | 131/143; 91.61 (85.8 to 95.59) | 0.5 (0.24 to 0.72) | 102/143; 71.33 (63.18 to 78.58) | 0.64 (0.53 to 0.74) |
| [48.49/M] Statistical methods for additional analyses (e.g., subgroup and adjusted analyses, PK/PD, biomarker correlative analyses) | | | | |
| SPIRIT | 125/142; 88.03 (81.52 to 92.87) | 0.74 (0.62 to 0.84) | 102/142; 71.83 (63.67 to 79.05) | 0.69 (0.58 to 0.78) |
| CONSORT | 124/142; 87.32 (80.71 to 92.31) | 0.72 (0.59 to 0.83) | 101/142; 71.13 (62.93 to 78.42) | 0.67 (0.57 to 0.76) |
| [50.51/M] Clearly-defined analysis population (e.g., evaluable population for dose determination, safety and key outcomes) | | | | |
| SPIRIT | 128/143; 89.51 (83.29 to 94.01) | 0.48 (0.22 to 0.72) | 101/143; 70.63 (62.44 to 77.94) | 0.58 (0.45 to 0.68) |
| CONSORT | 131/143; 91.61 (85.8 to 95.59) | 0.5 (0.25 to 0.75) | 102/143; 71.33 (63.18 to 78.58) | 0.61 (0.5 to 0.7) |
| [52.53/N] Pre-specify handling strategies of events occurring after treatment initiation (e.g., how dosing delays will be handled) that affect either the interpretation or the existence of the measurements associated with the clinical question of interest | | | | |
| SPIRIT | 130/143; 90.91 (84.96 to 95.07) | 0.73 (0.57 to 0.87) | 110/143; 76.92 (69.15 to 83.55) | 0.74 (0.64 to 0.81) |
| CONSORT | 123/143; 86.01 (79.23 to 91.24) | 0.71 (0.56 to 0.81) | 108/143; 75.52 (67.64 to 82.32) | 0.74 (0.64 to 0.81) |
| [54.55/N] Statistical software and packages used for design (e.g., simulation) and to be used for planned analyses | | | | |
| SPIRIT | 125/143; 87.41 (80.84 to 92.37) | 0.8 (0.7 to 0.88) | 104/143; 72.73 (64.65 to 79.83) | 0.75 (0.67 to 0.82) |
| CONSORT | 120/143; 83.92 (76.85 to 89.52) | 0.72 (0.61 to 0.82) | 101/143; 70.63 (62.44 to 77.94) | 0.71 (0.62 to 0.8) |
| [56.57/M] Any decision-making group or safety review committee, alternatively, an explanation of why such a committee is not needed | | | | |
| SPIRIT | 126/141; 89.36 (83.06 to 93.92) | 0.65 (0.46 to 0.79) | 100/141; 70.92 (62.68 to 78.26) | 0.69 (0.57 to 0.77) |
| CONSORT | 123/141; 87.23 (80.58 to 92.26) | 0.65 (0.52 to 0.79) | 106/141; 75.18 (67.21 to 82.06) | 0.74 (0.64 to 0.82) |
| [58.59/M] Description of the plans for any interim data review (including data to be used for decision-making) and interim statistical analyses (e.g., safety/toxicity, dose [de-]escalation decisions) and stopping guidelines | | | | |
| SPIRIT | 131/142; 92.25 (86.56 to 96.07) | 0.66 (0.42 to 0.83) | 104/142; 73.24 (65.17 to 80.32) | 0.7 (0.6 to 0.8) |
| CONSORT | 121/142; 85.21 (78.29 to 90.61) | 0.55 (0.37 to 0.71) | 104/142; 73.24 (65.17 to 80.32) | 0.73 (0.64 to 0.81) |
| [60/M] For each group, the number of participants who were assigned to each dose level at each interim analysis (e.g., for dosing decisions), received intended treatment, and were analysed for the primary outcome and, if applicable, any other outcomes used to inform pre-planned adaptations | | | | |
| CONSORT | 129/142; 90.85 (84.85 to 95.03) | 0.57 (0.32 to 0.77) | 108/142; 76.06 (68.18 to 82.81) | 0.68 (0.56 to 0.79) |
| [61/N] Trial adaptation decisions made in light of the pre-planned decision-making criteria and observed accrued data | | | | |
| CONSORT | 133/142; 93.66 (88.31 to 97.06) | 0.74 (0.55 to 0.88) | 104/142; 73.24 (65.17 to 80.32) | 0.71 (0.62 to 0.8) |
| [62/M*] Baseline demographic and clinical characteristics across each dose level within each group, where appropriate | | | | |
| CONSORT | 122/140; 87.14 (80.44 to 92.2) | 0.61 (0.44 to 0.77) | 92/140; 65.71 (57.23 to 73.52) | 0.62 (0.52 to 0.72) |
| [63/M] For each group, the number of participants (denominator) included in each (interim/final) analysis across each dose level, and whether the analysis was by original assigned interventions | | | | |
| CONSORT | 126/141; 89.36 (83.06 to 93.92) | 0.44 (0.19 to 0.68) | 93/141; 65.96 (57.51 to 73.72) | 0.51 (0.4 to 0.63) |
| [64/M] For each primary and secondary outcome, results for each dose level within each group, and the estimated effect size and its precision if applicable | | | | |
| CONSORT | 124/142; 87.32 (80.71 to 92.31) | 0.38 (0.17 to 0.61) | 95/142; 66.9 (58.52 to 74.56) | 0.52 (0.4 to 0.63) |
| [65/N] Report interim results used to inform interim decision-making such as dose escalation, de-escalation or staying at the same dose | | | | |
| CONSORT | 120/142; 84.51 (77.49 to 90.03) | 0.51 (0.33 to 0.66) | 100/142; 70.42 (62.19 to 77.78) | 0.66 (0.56 to 0.75) |
| [66/M] Plans for collecting, assessing, reporting, and managing solicited and spontaneously reported adverse events and other unintended effects of trial interventions (e.g., prior to any planned next dosing) or trial conduct | | | | |
| SPIRIT | 128/141; 90.78 (84.75 to 95) | 0.27 (0 to 0.66) | 96/141; 68.09 (59.71 to 75.68) | 0.48 (0.36 to 0.63) |
| [67/M] All important toxicities and adverse events reported by dose level in each group (for specific guidance see CONSORT for harms) | | | | |
| CONSORT | 134/141; 95.04 (90.04 to 97.98) | 0.36 (0 to 0.92) | 102/141; 72.34 (64.18 to 79.53) | 0.49 (0.36 to 0.63) |
| [68.69/M*] Specify if and when results (e.g., safety/response outcomes) can be shared externally/were reported whilst the trial is still ongoing | | | | |
| SPIRIT | 119/137; 86.86 (80.03 to 92.02) | 0.72 (0.6 to 0.83) | 95/137; 69.34 (60.9 to 76.93) | 0.68 (0.57 to 0.76) |
| CONSORT | 117/137; 85.4 (78.36 to 90.85) | 0.72 (0.61 to 0.82) | 93/137; 67.88 (59.37 to 75.6) | 0.69 (0.6 to 0.78) |
| [70/M] Where the full trial protocol or the redacted version, with amendments (if any), can be accessed | | | | |
| CONSORT | 135/143; 94.41 (89.27 to 97.55) | 0.89 (0.79 to 0.95) | 104/143; 72.73 (64.65 to 79.83) | 0.78 (0.7 to 0.84) |
| [71/N] Where other relevant trial documents (Oversight Committee, Safety Review Charter, quality aspects of investigational medicinal product, investigators brochure, simulation report, this list is non-exhaustive) can be accessed | | | | |
| SPIRIT | 133/143; 93.01 (87.52 to 96.6) | 0.87 (0.76 to 0.93) | 103/143; 72.03 (63.91 to 79.21) | 0.74 (0.66 to 0.81) |
| [72/N] Where the full statistical analysis plan and other relevant trial documents (Oversight Committee, Safety Review/Data Monitoring Committee Charter, quality aspects of investigational medicinal product, investigators brochure, simulation report, this list is non-exhaustive) can be accessed | | | | |
| CONSORT | 126/143; 88.11 (81.65 to 92.92) | 0.78 (0.66 to 0.86) | 104/143; 72.73 (64.65 to 79.83) | 0.76 (0.69 to 0.84) |
| [73/N*] Dose transition pathways or dose decision paths (using, for example, a flow diagram or table) projecting in advance how a proposed dose-finding design will recommend doses based on participants' key outcomes (e.g., what the next dose would be if x out of y participants experience significant adverse events) | | | | |
| SPIRIT | 118/138; 85.51 (78.51 to 90.92) | 0.77 (0.67 to 0.86) | 97/138; 70.29 (61.92 to 77.76) | 0.72 (0.64 to 0.81) |
| [74.75/N*] Involvement of patients, service users, their carers, members of public or patient advocates in any aspect of the trial. Or reason why their involvement is/was not necessary | | | | |
| SPIRIT | 117/136; 86.03 (79.05 to 91.37) | 0.78 (0.67 to 0.86) | 96/136; 70.59 (62.17 to 78.09) | 0.76 (0.67 to 0.83) |
| CONSORT | 114/135; 84.44 (77.21 to 90.11) | 0.76 (0.64 to 0.85) | 92/135; 68.15 (59.58 to 75.9) | 0.77 (0.69 to 0.84) |
| [76.77/N*] Lay summary of the trial synopsis/results or where it can be accessed | | | |  |
| SPIRIT | 120/141; 85.11 (78.14 to 90.54) | 0.74 (0.62 to 0.83) | 102/141; 72.34 (64.18 to 79.53) | 0.74 (0.65 to 0.82) |
| CONSORT | 123/142; 86.62 (79.9 to 91.75) | 0.77 (0.66 to 0.86) | 104/142; 73.24 (65.17 to 80.32) | 0.75 (0.66 to 0.83) |
| [78/M] Identification as a dose-finding trial | | |  |  |
| CONSORT | 132/143; 92.31 (86.65 to 96.1) | 0.76 (0.58 to 0.87) | 104/143; 72.73 (64.65 to 79.83) | 0.71 (0.61 to 0.8) |
| [79/N] Dose decisions/adaptations were made in light of pre-planned decision-making criteria and observed accrued data | | | | |
| CONSORT | 127/142; 89.44 (83.18 to 93.97) | 0.79 (0.66 to 0.88) | 103/142; 72.54 (64.42 to 79.68) | 0.76 (0.67 to 0.83) |


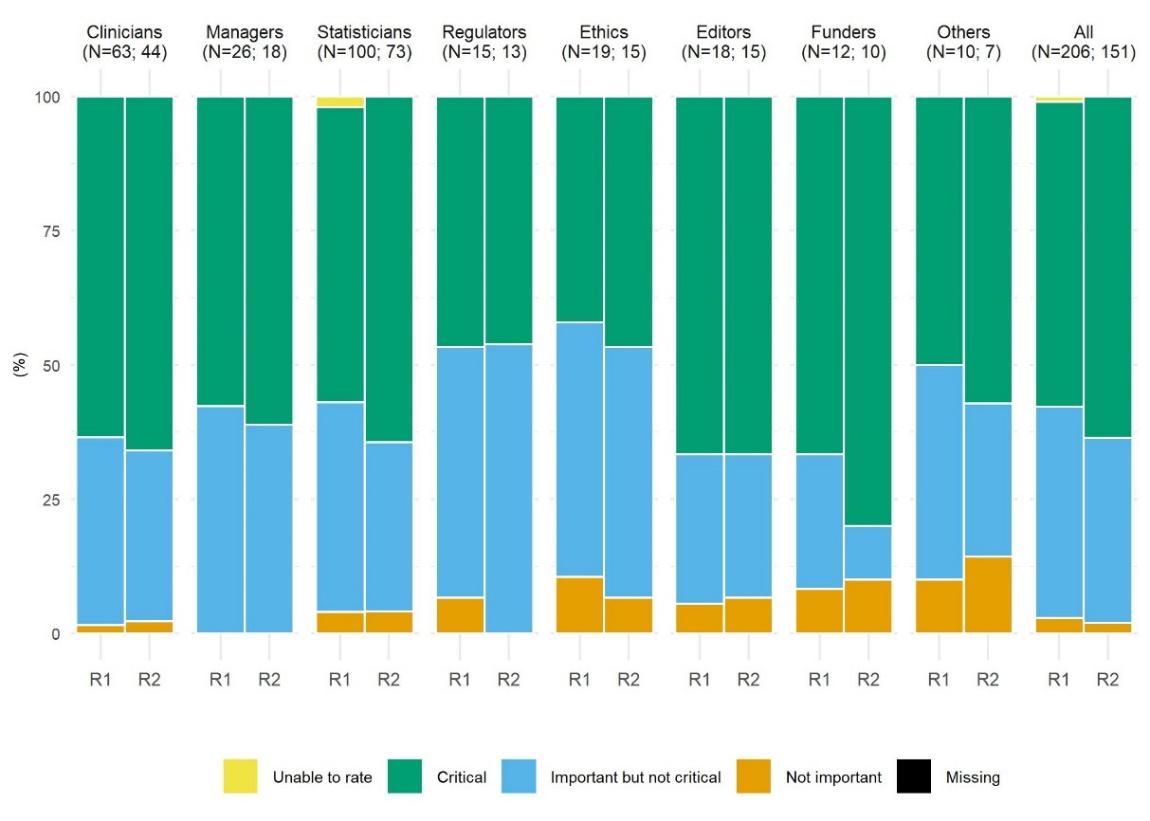


*Figure A9-3 Stacked bar plots of candidate item [17 SPIRIT-DEFINE] “Planned dosing regimens presented as a diagram or table, where applicable” by stakeholders. Note that multiple roles were allowed.*


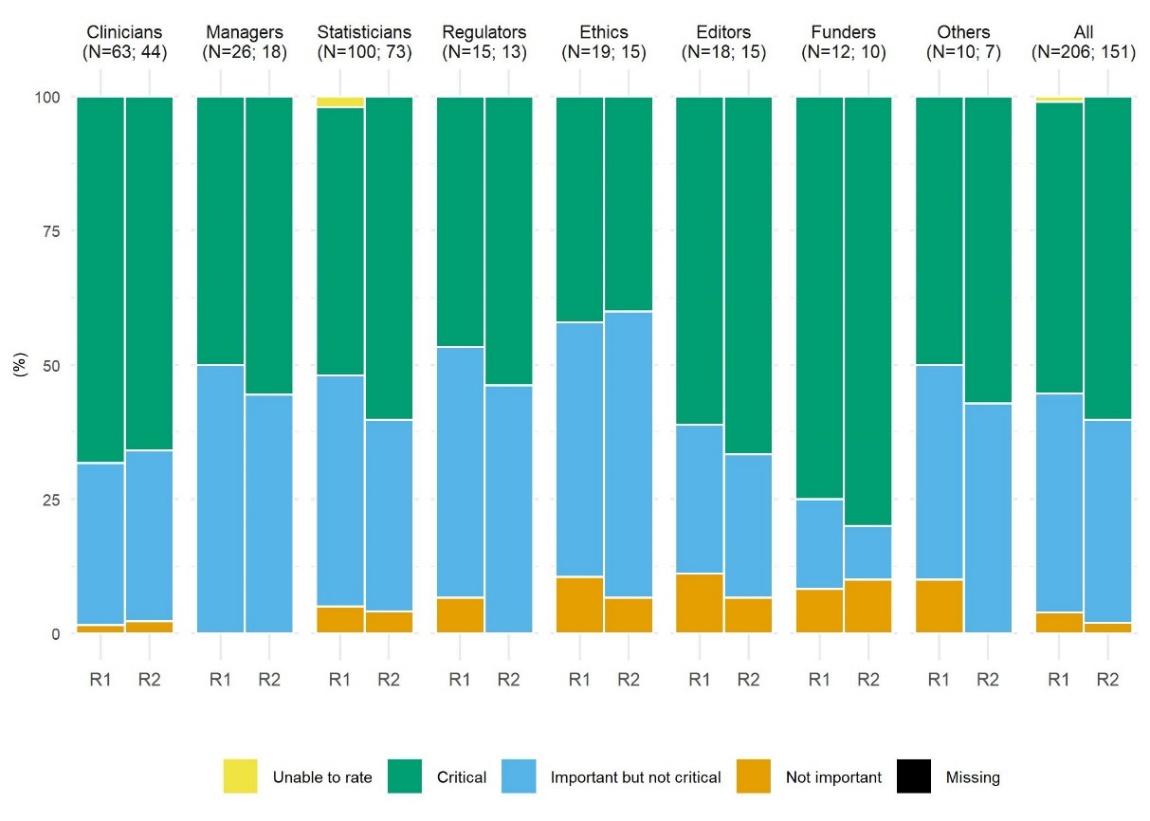


*Figure A9-4 Stacked bar plots of candidate item [18 CONSORT-DEFINE] “Planned and delivered dosing regimens presented as a diagram or table, where applicable” by stakeholders. Note that multiple roles were allowed.*


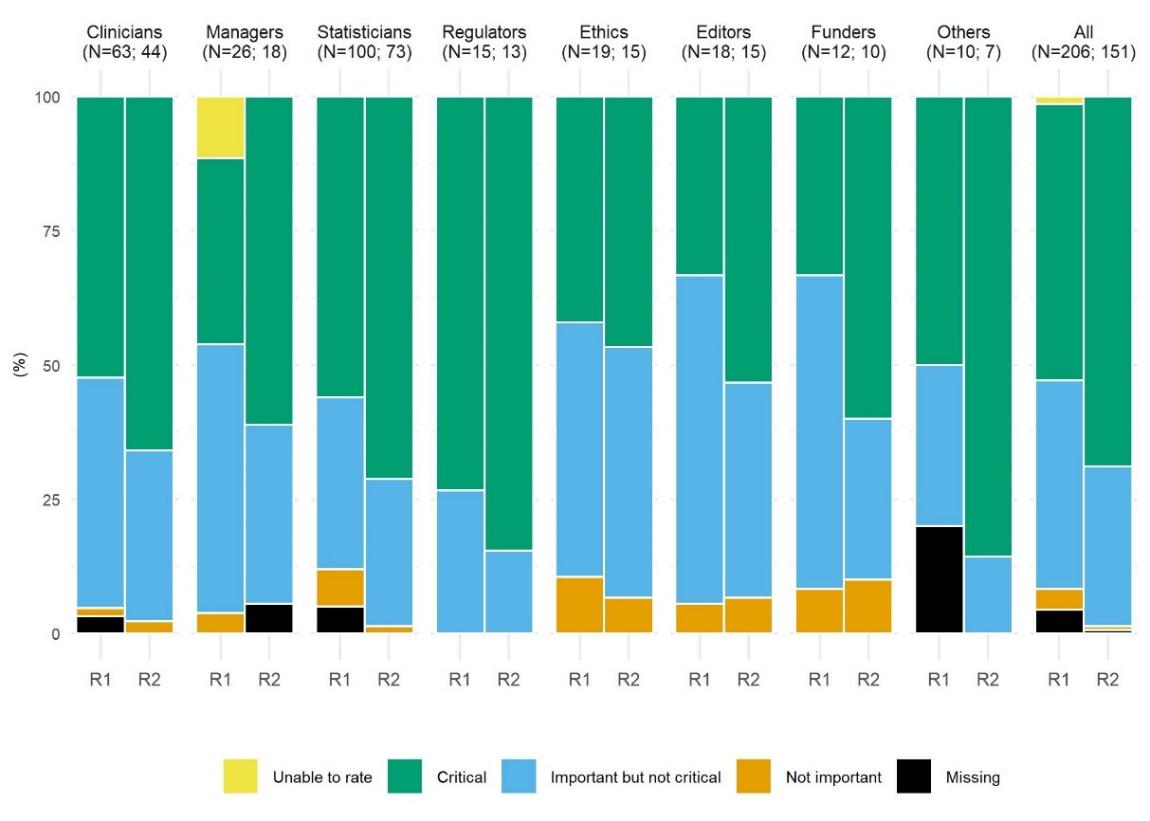


*Figure A9-5 Stacked bar plots of candidate item [48 SPIRIT-DEFINE] “Statistical methods for additional analyses (e.g., subgroup and adjusted analyses, PK/PD, biomarker correlative analyses)” by stakeholders. Note that multiple roles were allowed.*


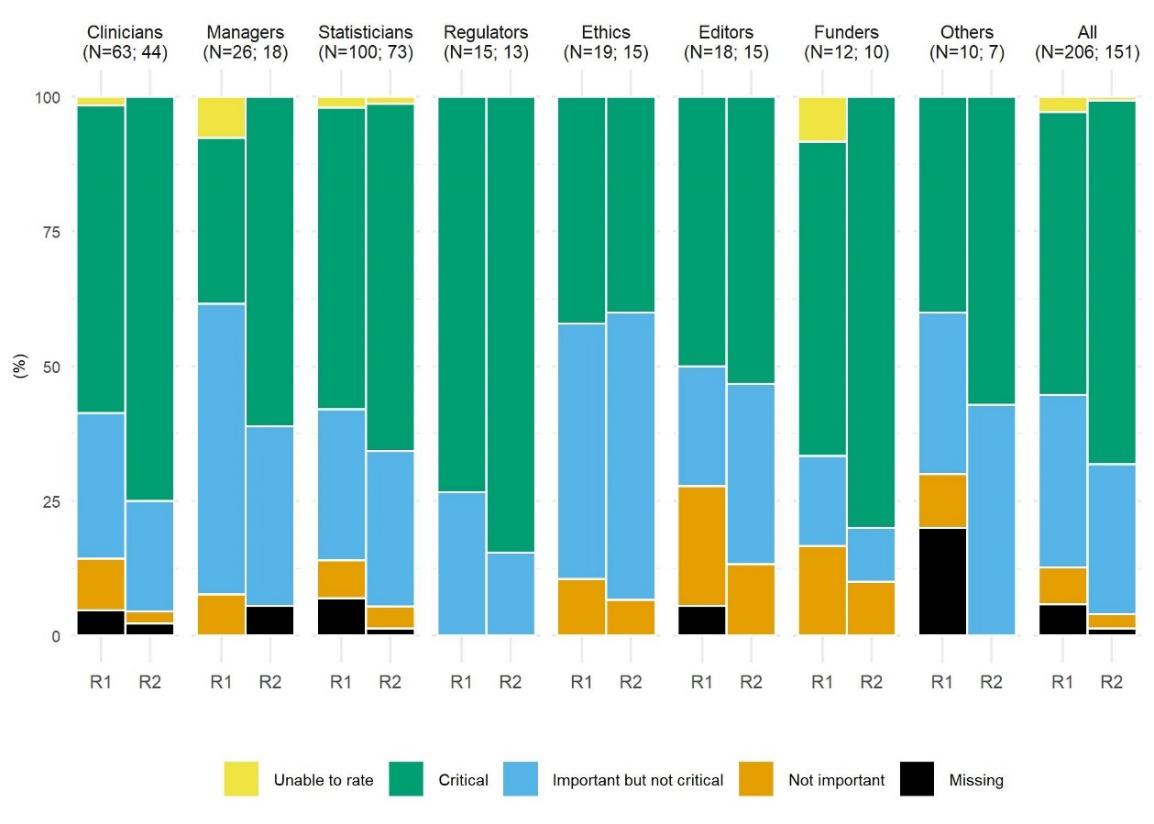


*Figure A9-6 Stacked bar plots of candidate item [69 CONSORT-DEFINE] “Specify if and when results (e.g., safety/response outcomes) were reported whilst the trial was still ongoing” by stakeholders. Note that multiple roles were allowed.*


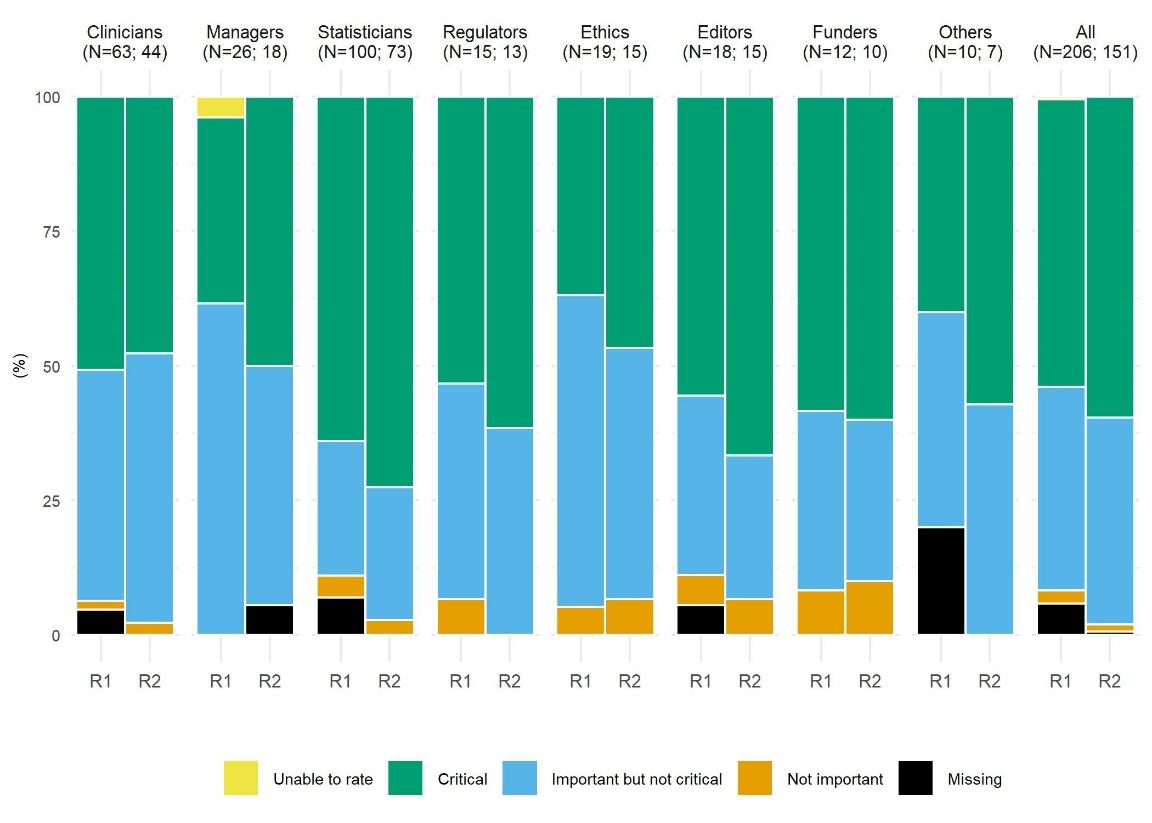


*Figure A9-7 Stacked bar plots of candidate item [70 SPIRIT-DEFINE] “Where the full trial protocol or the redacted version, with amendments (if any), can be accessed” by stakeholders. Note that multiple roles were allowed.*


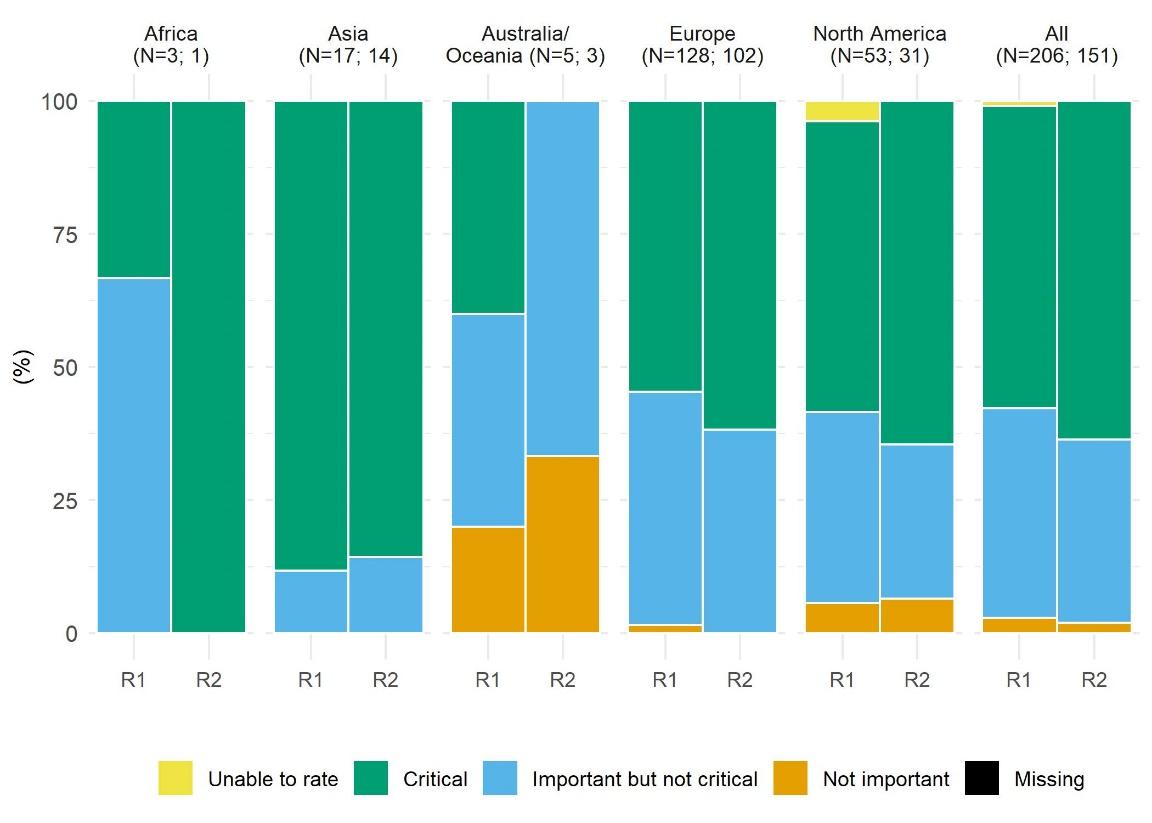


*Figure A9-8 Stacked bar plots of candidate item [17 SPIRIT-DEFINE] “Planned dosing regimens presented as a diagram or table, where applicable” by Continent.*


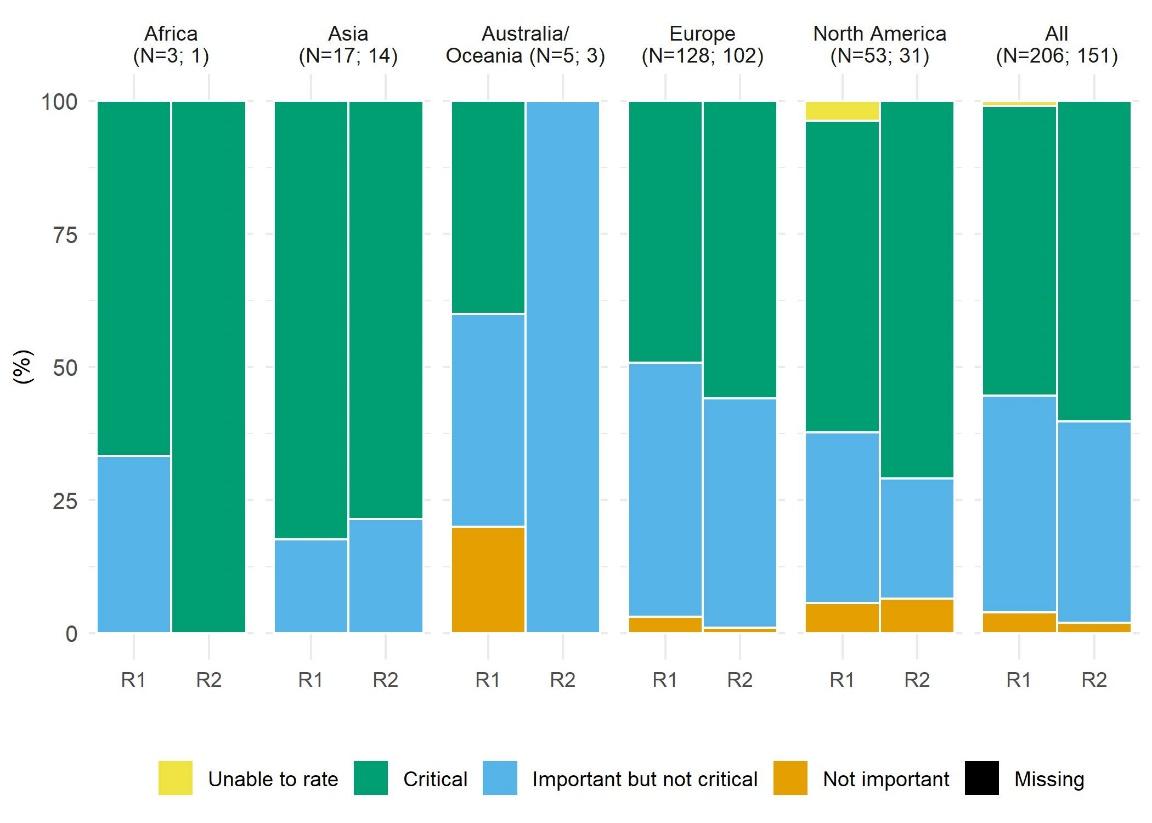


*Figure A9-9 Stacked bar plots of candidate item [18 CONSORT-DEFINE] “Planned and delivered dosing regimens presented as a diagram or table, where applicable” by Continent.*


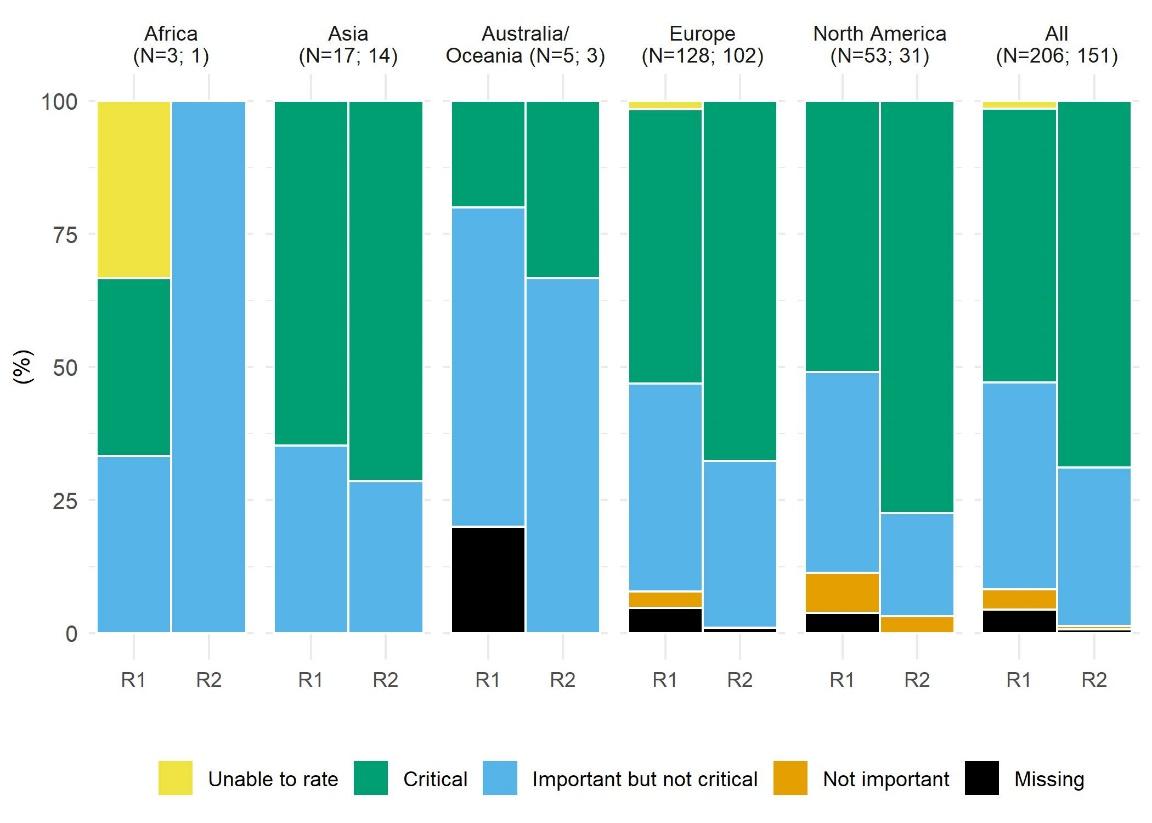


*Figure A9-10 Stacked bar plots of candidate item [48 SPIRIT-DEFINE] “Statistical methods for additional analyses (e.g., subgroup and adjusted analyses, PK/PD, biomarker correlative analyses)” by Continent.*


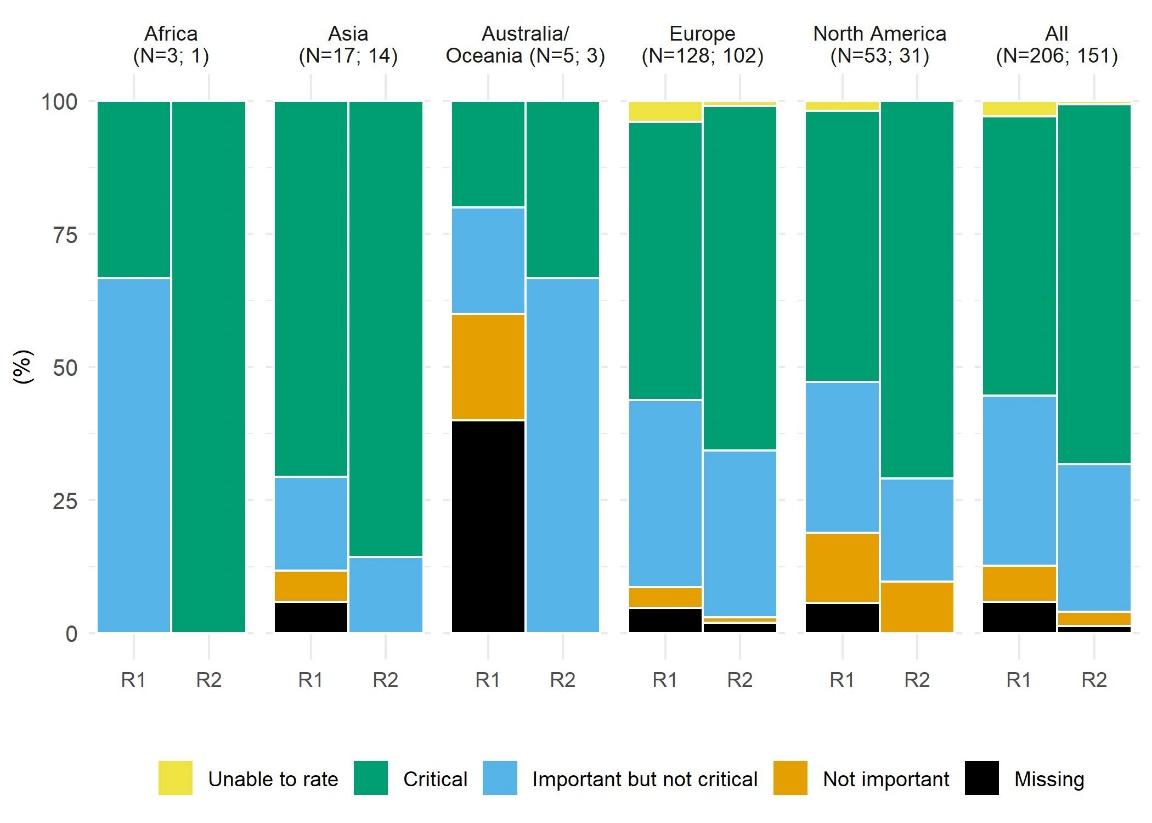


*Figure A9-11 Stacked bar plots of candidate item [69 CONSORT-DEFINE] “Specify if and when results (e.g., safety/response outcomes) were reported whilst the trial was still ongoing” by Continent.*


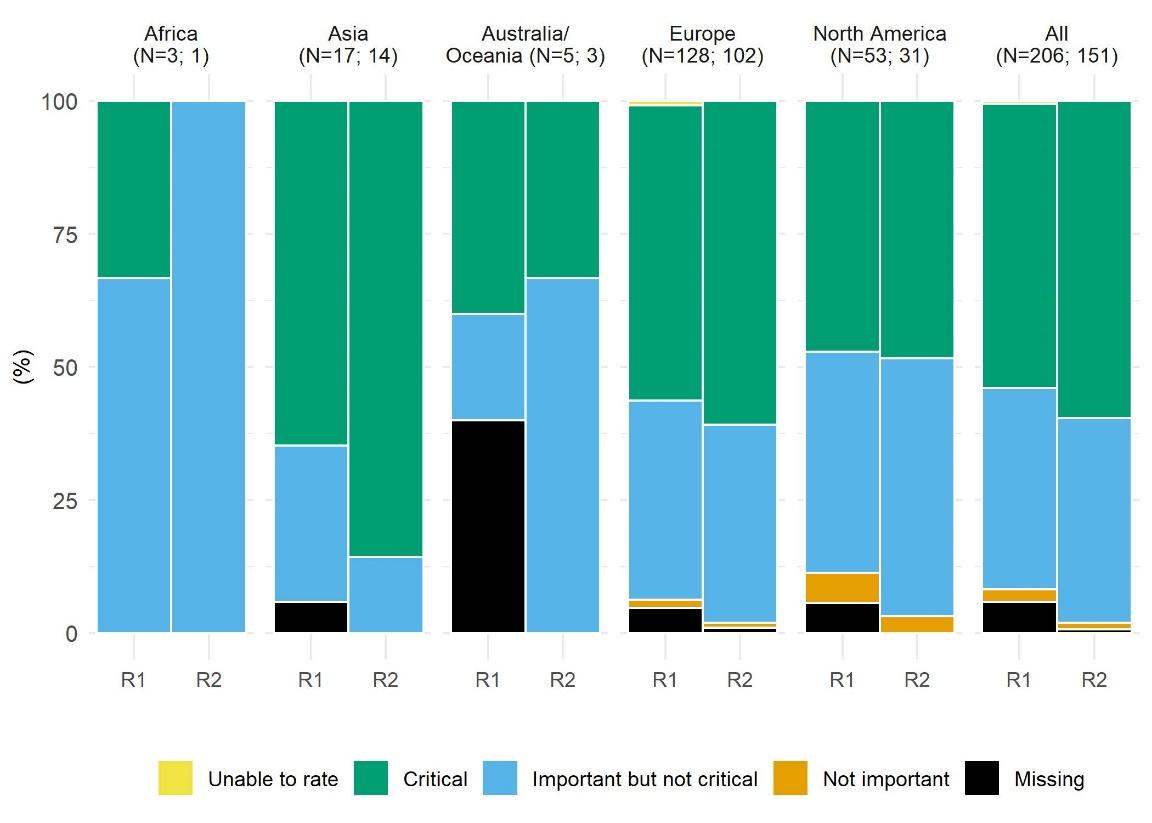


*Figure A9-12 Stacked bar plots of candidate item [70 SPIRIT-DEFINE] “Where the full trial protocol or the redacted version, with amendments (if any), can be accessed” by Continent.*


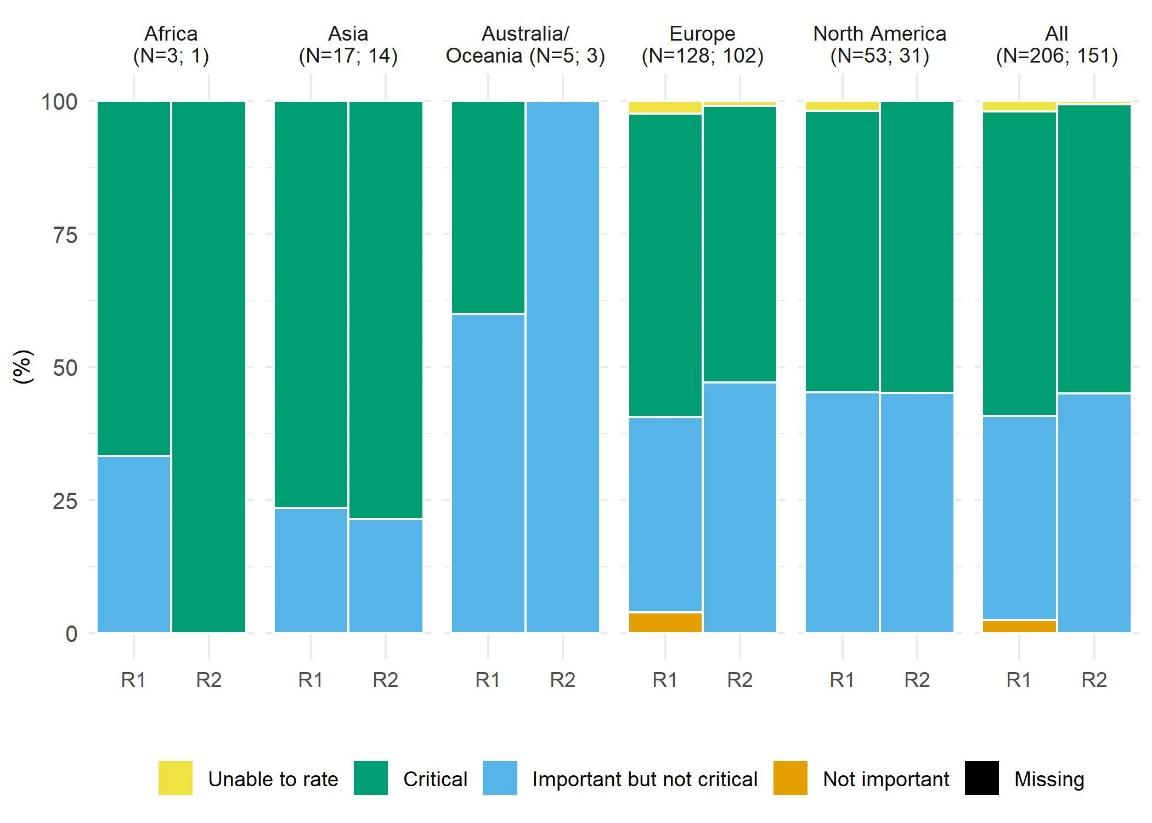


*Figure A9-13 Stacked bar plots of candidate item [5 SPIRIT-DEFINE] “Summary of findings from existing correlative biomarker, correlative and associated studies to support planned biomarker sub-study (if applicable)” by Continent.*
