# Supplementary material for: Development of consensus-driven SPIRIT and CONSORT extensions for early phase dose-finding trials: the DEFINE study
Source: BMC Med. 2023 Jul 5;21:246. doi: 10.1186/s12916-023-02937-0 (PMC10324137; doi:10.1186/s12916-023-02937-0)
Supplement: Supplementary file 10 — Additional file 10. List of candidate items to be discussed at the DEFINE Consensus meeting. [file 12916_2023_2937_MOESM10_ESM.docx]

# List of candidate items to be discussed at the DEFINE Consensus meeting

The table below provides the list of candidate items that did not meet the inclusion threshold and would be discussed at the DEFINE Consensus meeting. [x] denotes the candidate item number as displayed in the Delphi survey.

| **SPIRIT-DEFINE** | **CONSORT-DEFINE** |
| --- | --- |
| [5] Summary of findings from existing correlative biomarker, correlative and associated studies to support planned biomarker sub-study (if applicable)  [17] Planned dosing regimens presented as a diagram or table, where applicable  [41] Plans for recruitment/screening slots for sequential cohorts of participants  [45] Specify if the data management plans in the initial dose-finding component are different from subsequent stages (e.g., expansion cohort(s) or Phase II) of the trial  [48] Statistical methods for additional analyses (e.g., subgroup and adjusted analyses, PK/PD, biomarker correlative analyses)  [54] Statistical software and packages used for design (e.g., simulation) and to be used for planned analyses  [71] Where other relevant trial documents (Oversight Committee, Safety Review Charter, quality aspects of investigational medicinal product, investigators brochure, simulation report, this list is non-exhaustive) can be accessed  [73N] Dose transition pathways or dose decision paths (using, for example, a flow diagram or table) projecting in advance how a proposed dose-finding design will recommend doses based on participants’ key outcomes (e.g., what the next dose would be if x out of y participants experience significant adverse events)  [74] Involvement of patients, service users, their carers, members of public or patient advocates in any aspect of the trial and/or reason why their involvement is not necessary  [76] Lay summary of the trial synopsis or where it can be accessed | [6] Summary of findings from existing correlative biomarker, correlative and associated studies to support planned biomarker sub-study (if applicable)  [18] Planned and delivered dosing regimens presented as a diagram or table, where applicable  [55] Statistical software and packages used  [69] Specify if and when results (e.g., safety/response outcomes) were reported whilst the trial was still ongoing  [70] Where the full trial protocol or the redacted version, with amendments (if any), can be accessed  [72] Where the full statistical analysis plan and other relevant trial documents (Oversight Committee, Safety Review/Data Monitoring Committee Charter, quality aspects of investigational medicinal product, investigators brochure, simulation report, this list is non-exhaustive) can be accessed  [75] Involvement of patients, service users, their carers, members of public or patient advocates in any aspect of the trial. Or reason why their involvement was not necessary  [77] Lay summary of the trial results or where it can be accessed  [Added at R2] Access (or link to) to code/functions used for simulation studies  CONSORT-DEFINE for Abstract: [79] Dose decisions/adaptations were made in light of pre-planned decision-making criteria and observed accrued data |
