# Supplementary material for: Development of consensus-driven SPIRIT and CONSORT extensions for early phase dose-finding trials: the DEFINE study
Source: BMC Med. 2023 Jul 5;21:246. doi: 10.1186/s12916-023-02937-0 (PMC10324137; doi:10.1186/s12916-023-02937-0)
Supplement: Supplementary file 11 — Additional file 11. Qualitative analysis. Table A11-1; Figure A11-1. Table A11-1. Themes and codes identified in the general comments received in Delphi survey. Figure A11-1. Tree diagram of the comments received per theme. [file 12916_2023_2937_MOESM11_ESM.docx]

# Qualitative Analysis

An inductive thematic analysis using a semantic approach was performed on all free-text comments (Clarke and Braun, 2022). Both rounds of the Delphi survey received general comments from participants about the entire survey. Twenty-four codes were generated for the 30 general comments received for the Delphi survey. These codes were organised into six higher-level themes: content consideration, Delphi process feedback, guideline structure, participant characteristics, patient and public involvement, and unrelated content (Table A11-1). “Guideline Structure” was the most prominent theme (referred to 26 times) in the general comments, whilst comments about the Delphi process were less frequent (referred to 3 times) (Figure A11-1). Three (10%) participants who gave general comments expressed that several items were oncology-focused and could not be used for non-oncology or healthy volunteers’ trials. Therefore, we modified some items to improve their applicability.

**Table A11-1: Themes and codes identified in the general comments received in Delphi survey**

| Themes | Sub-themes | Descriptive summary | Exemplar quote |
| --- | --- | --- | --- |
| Content Consideration | Adverse events | Participants’ free text comments on the whole survey included a number of suggestions relating to the content of the checklists. Specifically, participants commented on a need for checklists to include content relating to handling and reporting of adverse events, success criteria, and results per dose level. Inclusion of decision-making criteria around dose transitions and other items to include in the checklists were suggested in the general comments. Concerns relating to the use of language biased toward oncology-focused trials were also captured in general free-text comments. | “...there is inconsistency in reporting of serious adverse events - some manuscripts report the number of patients overall who had any adverse events, some report the number of adverse events but without context...”  “Unevaluable patients often occur when making dose-transition decisions. How to deal with that may also be standard and included in the protocol.”  “Language still seems too heavily oriented towards Oncology studies. If you want this to be Oncology specific, then say so in the title (e.g., there seems to be no option in 38/39 for a simple parallel group fixed sample size design, people outside Oncology might not even know what you are talking about...” |
|  | Bias |  |  |
|  | Changing-evolving methodological approach to DF trials |  |  |
|  | Characteristics of early phase trials |  |  |
|  | Decision-making dosing |  |  |
|  | Importance of language and terminology |  |  |
|  | Oncology focus |  |  |
|  | Selecting content for inclusion – various |  |  |
| Feedback on Delphi process | Feedback on Delphi process | There were comments received on the Delphi process, including on the usability and design of the survey and its iterative nature, i.e., reviewing the survey after a set period of time. | “Really useful to see others' scores and to reflect upon this.”  “Also beneficial to review this after a period of time, as this was quite a long list of criteria to score and I had simply made mistakes the first time around on some criteria. I appreciate you could save it and come back to it, but I found it helpful to review it in one sitting.” |
|  | Iterations |  |  |
| Guideline Structure | Availability of information | This theme describes comments on overarching concepts that may be used to inform guideline development, such as how the availability of information in the public domain should inform the content/structure of guidelines and how the guidelines should be structured to meet end user needs to ensure relevance and impact. It also encompasses comments on the importance of guideline development for dose-finding trial reports and protocols and any comments on the extension’s relationship to the original SPIRIT and CONSORT statements. Participants’ views of what should be covered in the scope of the checklists and information that participants’ believed should be standardised in dose-finding protocols and studies were also captured in this theme. | “The whole approach seems to be completely focused on oncology dose escalation studies. That's fine, but then it should be a guidance for just those studies. Or, to keep the current scope, consider consulting a group of people with experience with non-oncology dose finding trials that have very different considerations and might need more guidance on how to report results.”  “I found the SPIRIT and CONSORT dimension confusing. In cases where there were equivalent SPIRIT and CONSORT items it was not clear to me why both should be assessed.”  “... making dose-transition decisions. How to deal with that may also be standard and included in the protocol.” |
|  | End users |  |  |
|  | Importance of guideline development |  |  |
|  | Relationship between SPIRIT and CONSORT |  |  |
|  | Remit-scope of guideline |  |  |
|  | Standardisation |  |  |
| Participant characteristics | Characteristics of participants covered by guideline | Comments stated that trial participant factors should be considered when developing the content of the early phase dose-finding trial guidelines. Characteristics of healthy volunteers, placebo control groups, and unevaluable patients were suggested by survey participants for consideration when developing the DEFINE checklists. Participants felt that characteristics of trials with these populations were not taken into consideration in the checklist. | ““I am not sure to what extent two distinctions that may be important are being made between a) first in man studies and other phase one studies b) studies in patients and studies in healthy volunteers. Different items may be appropriate.”  “Inevaluable patients often occur when making dose-transition decisions. How to deal with that may also be standard and included in the protocol.” |
|  | Handling of unevaluable participants |  |  |
|  | Healthy volunteers |  |  |
|  | Patient trials |  |  |
|  | Placebo controls |  |  |
| Patient and public involvement | Patient engagement | Some comments described PPI considerations, the reporting of PPI in EPDF protocols and trial reports, and the degree to which the proposed checklists are patient-centred. | “I'd ask the faculty to consider how many participants in this process are bringing a specific participant perspective vs. someone who does/facilitates clinical trials. While reporting on PPI and summaries of results may seem like a burden or not as important to people doing trials (I'm generalizing here, and recognize this), this also represents a very large step forward from the perspective of patient partners on trials teams or participants in trials, where these items often feel like an after thought.”  “The PPI is too broad in the current questionnaire. As we move towards better engagement with people who are 'targets' for interventions, inclusion at different stages of the trial development is critical. This can influence future recruitment and ultimately internal validity as well as uptake after full trials.” |
|  | Patient-centredness |  |  |
| Unrelated content | | Comments of no relevance to the Delphi process or its contents, not included in the analysis | “Thank you for the opportunity to participate in the survey.” |

**Figure A11-1: Tree diagram of the comments received per theme
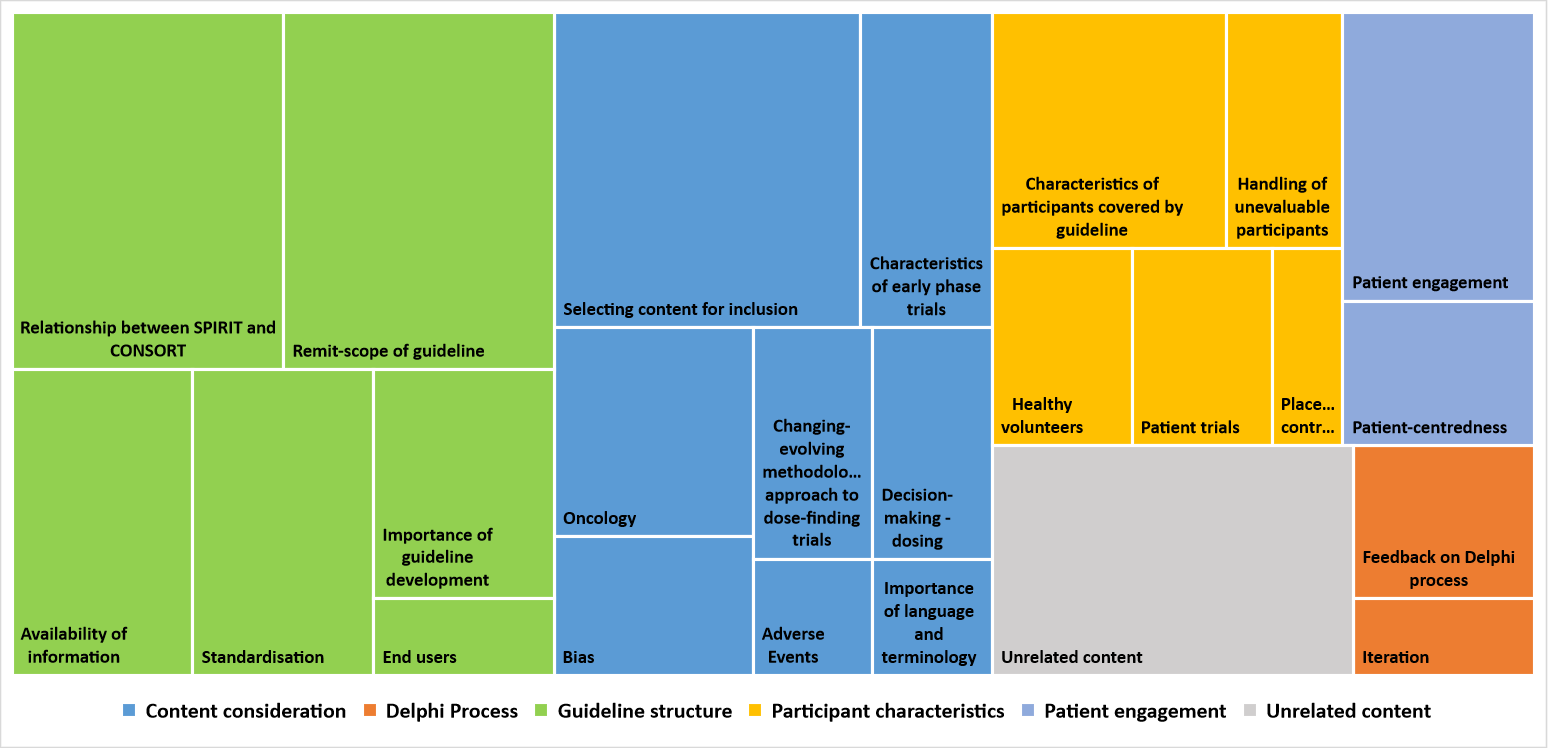
**
